# Identification of molecular patterns and prognostic models of epithelial–mesenchymal transition- and immune-combined index in the gastric cancer

**DOI:** 10.3389/fphar.2022.958070

**Published:** 2022-08-09

**Authors:** Xiuyuan Zhang, Yiming Li, Pengbo Hu, Liang Xu, Hong Qiu

**Affiliations:** Department of Oncology, Tongji Hospital, Tongji Medical College, Huazhong University of Science and Technology, Wuhan, China

**Keywords:** epithelial–mesenchymal transition, gastric cancer, tumor microenvironment, immunotherapy, biomarker

## Abstract

**Background:** Epithelial–mesenchymal transition (EMT) and the immune microenvironment play important roles in the progression of gastric cancer (GC), but the joint role of both in GC is not clear.

**Methods:** We identified EMT- and immune-related genes (EIRGs), and the molecular subtypes of EIRGs were identified by unsupervised cluster analysis. Then, we constructed an accurate EIRG_score model by using differential genes of molecular subtypes. The correlation of EIRG_score with prognosis, immune infiltration, gene mutation, chemotherapeutic drug sensitivity, and immunotherapy response was comprehensively analyzed. In addition, we investigated the biological function of EIRG_score via *in vitro* experiments.

**Results:** A total of 808 GC patients were classified into two molecular subtypes, which were enriched in EMT and immune-related biological pathways and significantly correlated with prognosis and immune infiltration. The constructed EIRG_score had an important role in predicting prognosis and immunotherapeutic response. The higher EIRG_score was associated with worse prognosis, higher abundance of immunosuppressive cell infiltration, lower immune checkpoint genes expression, lower tumor mutation burden, microsatellite instability-high, lower chemotherapeutic drug sensitivity, and poorer immunotherapeutic response.

**Conclusion:** EIRG_score may be used as a biomarker to assess prognosis and guide precise treatment.

## Introduction

Gastric cancer (GC) is one of the most common digestive tumors, ranking fifth in incidence and mortality rates worldwide (Sung et al., 2021). The 5-year survival rate of GC is only approximately 20% (Etemadi et al., 2020). The current treatment for GC is mainly radical surgery, and the survival rate of early GC is up to 90% after surgical resection, but the treatment for middle and advanced GC is not optimistic, and conventional chemotherapy does not achieve the desired effect (Thrumurthy et al., 2015; Li et al., 2021). At present, immunotherapy has achieved a series of promising results in the treatment of GC (Coutzac et al., 2019). However, immunotherapy needs to identify specific populations to be more effective; therefore, we urgently need new biomarkers that have a role in identification.

The tumor microenvironment (TME) is a heterogeneous structure composed of tumor cells and immune cells, stromal cells, and so on. Cells in the TME interact in a paracrine manner with other cell types, which enables tumor cells to escape host immune surveillance (Sadeghi Rad et al., 2021). The GC microenvironment is mainly composed of stromal and immune cells with immune escape characteristics, such as cancer-associated fibroblasts (CAFs), tumor-associated macrophages (TAMs), and T regulatory cells (Tregs) (Seeneevassen et al., 2021); therefore, it is considered to be an immunosuppressive tumor.

Epithelial–mesenchymal transition (EMT) is a process by which epithelial cells acquire mesenchymal characteristics that promote tumor invasion metastasis and drug resistance (Pastushenko and Blanpain, 2019). Previous studies have identified that EMT can affect TME. Epithelial tumors are infiltrated with large numbers of cytotoxic CD8^+^ T cells, but tumors with mesenchymal function contain Tregs cells and TAMs and can polarize into M2 subtypes (Dongre et al., 2017). EMT can also decrease the level of MHC class I on the cell surface and escape the killing function of T cells (Garcia-Lora et al., 2003) and can also induce cancer cells to express PD-L1, causing immune escape (Noman et al., 2017). In addition, TME components such as CAFs and TAMs can secrete growth factors and cytokines such as transforming growth factor-β (TGF-β) and interleukin-6 (IL-6), which can promote EMT (Dongre and Weinberg, 2019). Thus, EMT and TME interactions affect tumor progression.

In this study, we focused on the interaction between EMT and immunity. First, we obtained EMT- and immune-related genes (EIRGs) and classified GC patients into two molecular subtypes according to EIRGs. Then, patients were classified into two genetic subtypes based on differentially expressed genes (DEGs) identified by molecular subtypes. We further established the EIRG_score to predict overall survival (OS) and explored the immune status of GC to predict the response to immunotherapy.

## Materials and methods

### Data collection

We downloaded transcriptome data and clinical information of GC patients through the Cancer Genome Atlas (TCGA) database and the Gene Expression Omnibus (GEO) database. RNA sequencing data in the form of fragments per kilobase million (FPKM) and somatic mutation data in the form of MAF were downloaded via TCGA-STAD (n = 407), and the FPKM form was converted to transcripts per kilobase million form. We collected GSE84437 (n = 433) from the GEO database and combined and normalized the two datasets using the “ComBat” function of the “affy” and “sva” packages of R.

### Clustering analysis of EIRGs

In total, 1184 EMT-associated genes and 1959 immune-related genes were obtained from previous studies and the ImmPort database (https://www.immport.org/) (Gao et al., 2021). We intersected the EMT- and immune-related genes using a Venn diagram and subsequently performed differential expression analysis (FC > 1, *p* < 0.05) using the “limma” package to obtain 82 DEGs as EIRGs. The EIRGs were subjected to unsupervised clustering analysis by the “ConsensusClusterPlus” package. Principal component analysis (PCA) was then performed using the “stat” package to investigate the variability of different molecular subtypes. Detailed data are available in Supplementary Table S2.

### Gene set variation analysis

We downloaded the “h.all.v7.4. symbols” geneset from the GSEA-MSigDB database (http://www.gsea-msigdb.org/) and performed Gene set variation analysis (GSVA) to explore the biological role of different clusters using the “GSVA” package. The cutoff was logFC > 0.1 and adj.P.Val < 0.05, and GO and KEGG analyses were performed using the “clusterProfiler” package (Yu et al., 2012), with *p* < 0.05 as a filtering condition.

### Immune cell infiltration analysis

To investigate different molecular subtypes of TME, we performed immune cell infiltration analysis using ssGSEA and the “CIBERSORT” algorithm (Newman et al., 2015) to assess the relative abundance of M2 macrophages, T, myeloid-derived suppressor cells (MDSCs) and other immune cells. To ensure the accuracy of the results, we only included results with *p* < 0.05.

### Gene enrichment analysis

GO and KEGG enrichment analysis can be used as a way to explore gene function. In this study, we divided the expression of KIF2C into high- and low-risk groups according to the median and then performed enrichment analysis using the “clusterprofiler” (Yu et al., 2012) in R.

### Differentially expressed gene analysis of molecular subtypes of EIRGs.

We obtained 5,503 DEGs by using the “limma” package for differential analysis of different molecular subtypes with a screening criterion of *p* < 0.001. We obtained 1,669 genes associated with prognosis by univariate Cox regression of DEGs with *p* < 0.05 as the screening criterion. Gene clustering analysis was then performed using the “ConsensusClusterPlus” package to obtain GeneCluster.

### Construction and validation of the EIRG_score model

We constructed a prognostic model consisting of 18 genes using Lasso regression and multivariate Cox regression of prognosis-related DEGs with the “glmnet” package of R. EIRG_score was calculated using the following equation: Risk score = (exp gen1 × coef gen1) + (exp gen2 × coef gen2) +... + (exp gen18 × coef gen18), where exp is the value of gene expression and coef is the estimated regression coefficient. Patients were classified into high- and low-risk groups by using the median of the risk score. Survival analysis was performed using the “survival” package and the “survminer” package. ROC curves at 1, 3, and 5 years were plotted using the “timeROC” package.

### Constructing and evaluating nomogram

The nomogram can be used for multiple indicators to predict disease progression (Iasonos et al., 2008), and we constructed the nomogram by integrating clinicopathological data and EIRG_score through the “rms” package to predict 1-, 3-, and 5-year survival rates. Calibration curves were used to assess the agreement of the nomogram with the actual situation.

### Assessing the relationship between EIRG_score and immunotherapy response

Tumor immune dysfunction and exclusion (TIDE) algorithm predicts the response of a single sample or subtype to immune checkpoint inhibitors (ICIs) (Jiang et al., 2018), and immunophenoscores (IPS) can predict immunotherapy response. TIDE scores can be obtained from http://tide.dfci.harvard.edu/, and immunotherapy cohort IPS data can be obtained from the TCIA database (http://tcia.at/). The correlation of EIRG_score with TIDE and IPS was plotted by the “ggpubr” package.

### *In vitro* experimental validation

All cell lines in this study were obtained from the Laboratory of Oncology, Tongji Hospital, Huazhong University of Science and Technology. GES-1, BGC-823, and SGC-7901 were cultured using RPMI-1640 complete medium. qRT-PCR was used to verify the mRNA expression levels of the cell lines, siRNA transfection was used to knock down AKR1B1, and Cell Counting Kit 8 (CCK8) and transwell assay were used to study proliferation and migration. The above-detailed procedures are shown in Supplementary Table S1. All experiments were performed with three biological replicates.

### Immunohistochemistry

To verify the protein level expression of AKR1B1, we collected 5 GC tissues and five normal tissues from our hospital for immunohistochemical analysis. Tumor sections were first baked, subsequently desliced in xylene, and hydrated in graded ethanol; after retrieval in heat-sensitive citrate antigen, tissue sections were incubated overnight at 4°C℃ with the primary antibody to AKR1B1 (YT0194, Immunoway, USA) and for 60 min at 25°C with horseradish peroxidase–conjugated antibody. Staining was performed by incubation with diaminobenzidine. At last, these treated tissue sections were observed under a microscope.

### Statistical analysis

All statistical analyses were performed using R software (version 4.1.0). The Wilcoxon test was used for comparison between the two groups. Survival curves for each subgroup were plotted using the Kaplan–Meier plotter. Correlation coefficients were calculated using Spearman’s analysis. *p* < 0.05 was considered to be statistically significant.

## Results

### Identification of molecular subtypes of EIRGs in GC

**FIGURE 1:**
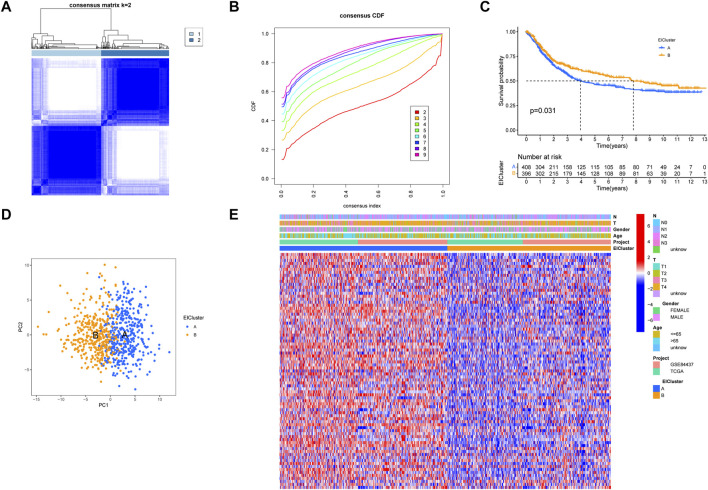
Construction of molecular subtypes of epithelial–mesenchymal transition- and immune-related genes (EIRGs) **(A)** consensus matrix heatmap defining two clusters (k = 2) and their correlation area. **(B)** cumulative distribution function graph. **(C)** survival curves of molecular subtypes. *p*-values are calculated using the log-rank test. **(D)** principal component analysis (PCA) between the two subtypes. **(E)** Clinicopathological characteristics and differences in expression levels of the two different subtypes.

After obtaining 1184 EMT- and 1959 immune-related genes, we obtained 199 intersecting genes associated with both immunity and EMT using a Venn diagram and subsequently performed differential expression analysis on the 199 intersecting genes to obtain 82 EIRGs (Supplementary Figure S1). To deeply investigate the expression characteristics of EIRGs in GC, we performed an unsupervised cluster analysis on GC patients (n = 808) based on EIRGs. When k = 2, the boundaries of the consistency matrix were clear (Figure 1A), and combined with the results of the cumulative distribution function (CDF) (Figure 1B), we took k = 2 as the optimal number of clusters and divided the cohort into two subtypes (EIcluster), namely, group A (n = 409) and group B (n = 399). By KM survival analysis (Figure 1C), we found that group A had a longer OS than group B (*p* < 0.05). To verify the stratification effect, we performed PCA analysis, and the results indicated a significant difference between the two subtypes and a good stratification effect (Figure 1D). Figure 1E shows the expression of EIRGs in the subtypes and the relationship with clinicopathological features.

### GSVA analysis of different molecular subtypes of EIRGs and immune infiltration analysis

**FIGURE 2:**
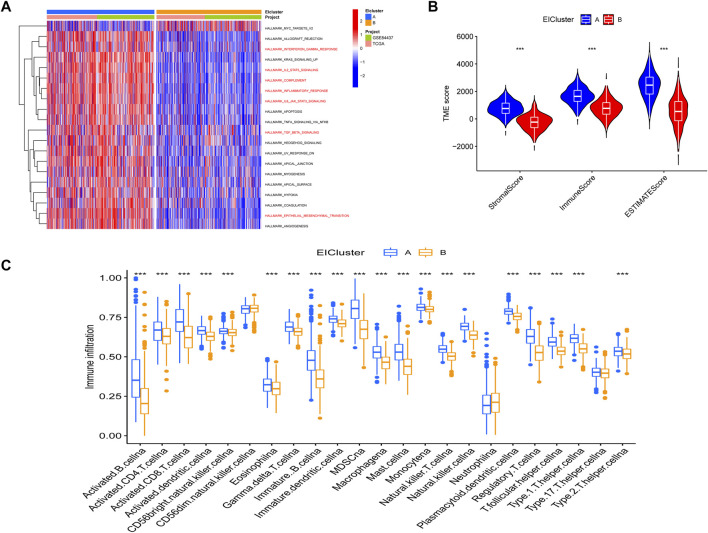
**(A)** Gene set variation analysis (GSVA) between two different subtypes, where red and blue represent activating and inhibiting pathways, respectively **(B)** correlation of molecular subtypes with tumor microenvironment score. **(C)** immune cell infiltration analysis of molecular subtypes. *p < 0.05, **p < 0.01, ***p < 0.001. *p-*values are calculated using the Wilcoxon test.

Through a GSVA analysis study, we found that subtype A was mainly enriched in EMT, interferon-gamma response, IL6 JAK STAT3 signaling, TNFA signaling via NFKB, and other biological activities (Figure 2A). To investigate the role of molecular subtypes in the immune microenvironment of GC, we evaluated the TME score of molecular subtypes by an estimate algorithm. A higher TME score means more abundant immune cells or stromal cells in TME, and the results showed that subtype A had a higher TME score than subtype B (Figure 2B). We then performed immune cell infiltration analysis of the subtypes by ssGSEA, and the results showed that subtype A had more abundant immune cell infiltration than subtype B, including MDSCs, Tregs, and macrophages (Figure 2C).

### Clustering analysis of genes related to molecular subtypes of EIRGs

**FIGURE 3:**
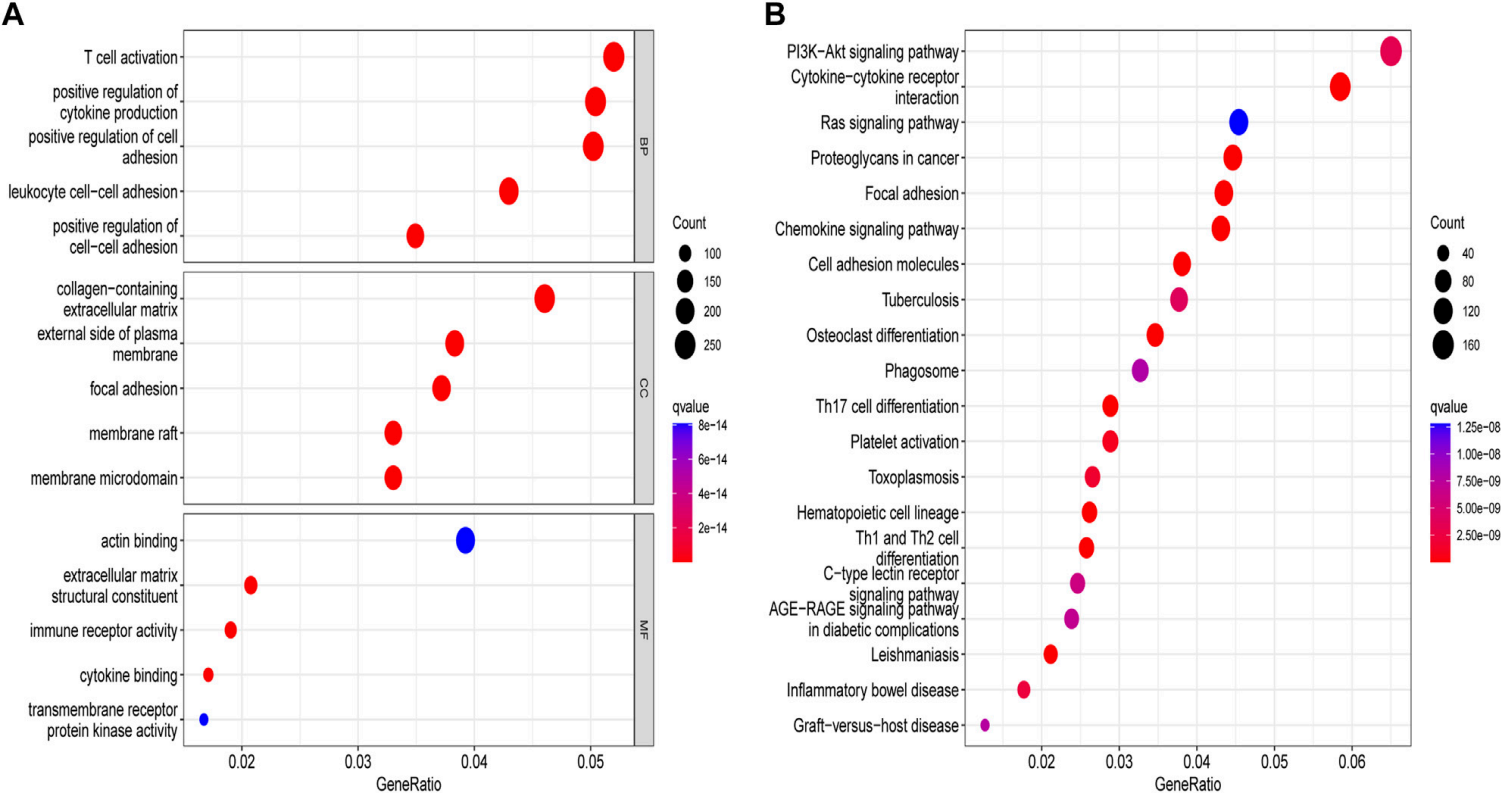
Enrichment analysis of molecular subtypes of DEGs. **(A)** gene ontology analysis **(B)** Kyoto encyclopedia of genes and genomes analysis.

**FIGURE 4:**
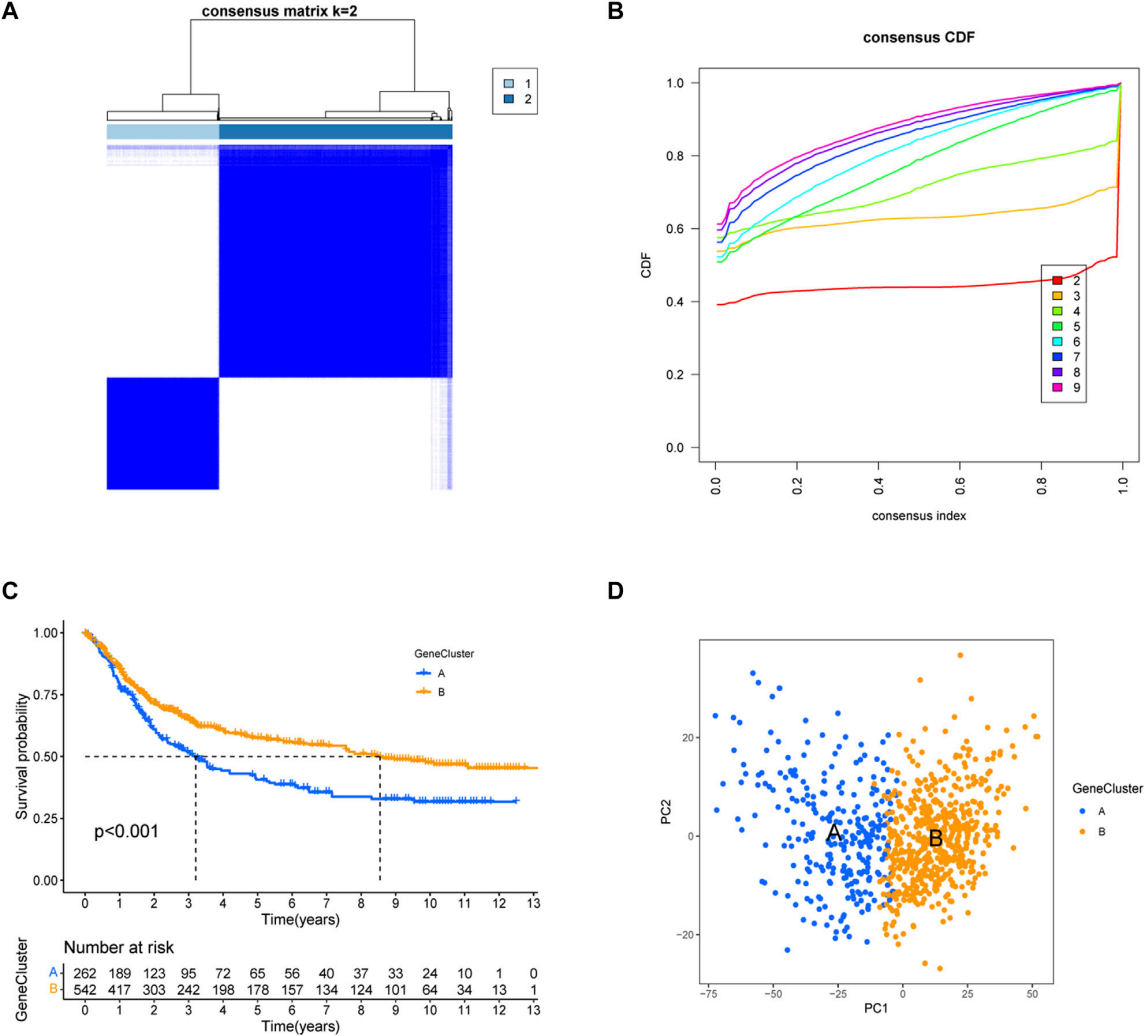
Construction of gene subtypes of EIRGs. **(A)** consensus matrix heatmap defining two clusters (k = 2) and their correlation area. **(B)** distribution function graph. **(C)** Survival curves of molecular subtypes. **(D)** PCA between the two subtypes.

**FIGURE 5:**
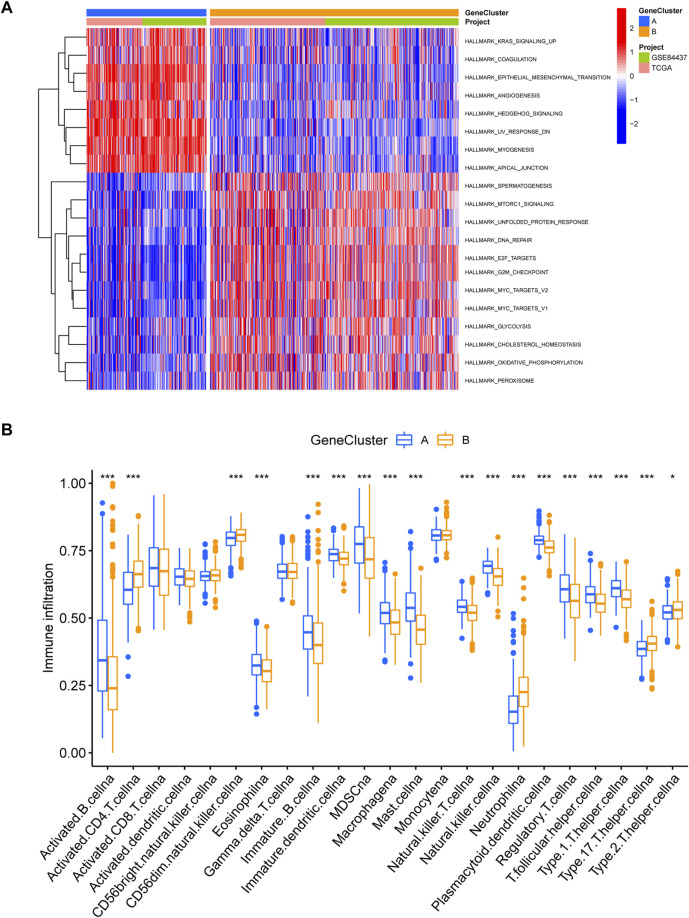
Immune infiltration analysis of gene subtypes **(A)** GSVA analysis of gene subtypes, where red and blue represent activation and suppression pathways, respectively. **(B)** immune cell infiltration analysis of gene subtypes. *p < 0.05, **p < 0.01, ***p < 0.001. The *p*-values are calculated using the Wilcoxon test.

We obtained 5,503 differential genes by using the limma package and enriched the DEGs via GO and KEGG analyses. GO analysis indicated that EIRGs are involved in the regulation of immune functions (Figure 3A), and KEGG analysis indicated that EIRGs are implicated in tumor- and immune-related pathways; as seen, EIRG has a vital function in tumor progression and immune regulation (Figure 3B). We subjected the DEGs to unsupervised cluster analysis, and the boundaries of the consistency matrix were clear when k = 2 (Figure 4A). Combined with the CDF results, we divided the cohort into two gene subtypes (GeneCluster) (Figure 4B). KM survival curves showed that group A had worse OS than group B (*p* < 0.001) (Figure 4C), and PCA results also showed significant differences between the two subtypes (Figure 4D). We then performed GSVA and immune infiltration analysis on the subtypes and found that group A was enriched in EMT and KRAS signaling, whereas group B was enriched in MTORC1 signaling, oxidative phosphorylation, and other biological activities (Figure 5A); the two subtypes were significantly correlated with immune infiltration, and the proportion of immune cells was greater in subtype A than in subtype B (Figure 5B).

### Construction and validation of the prognostic EIRG_score

**FIGURE 6:**
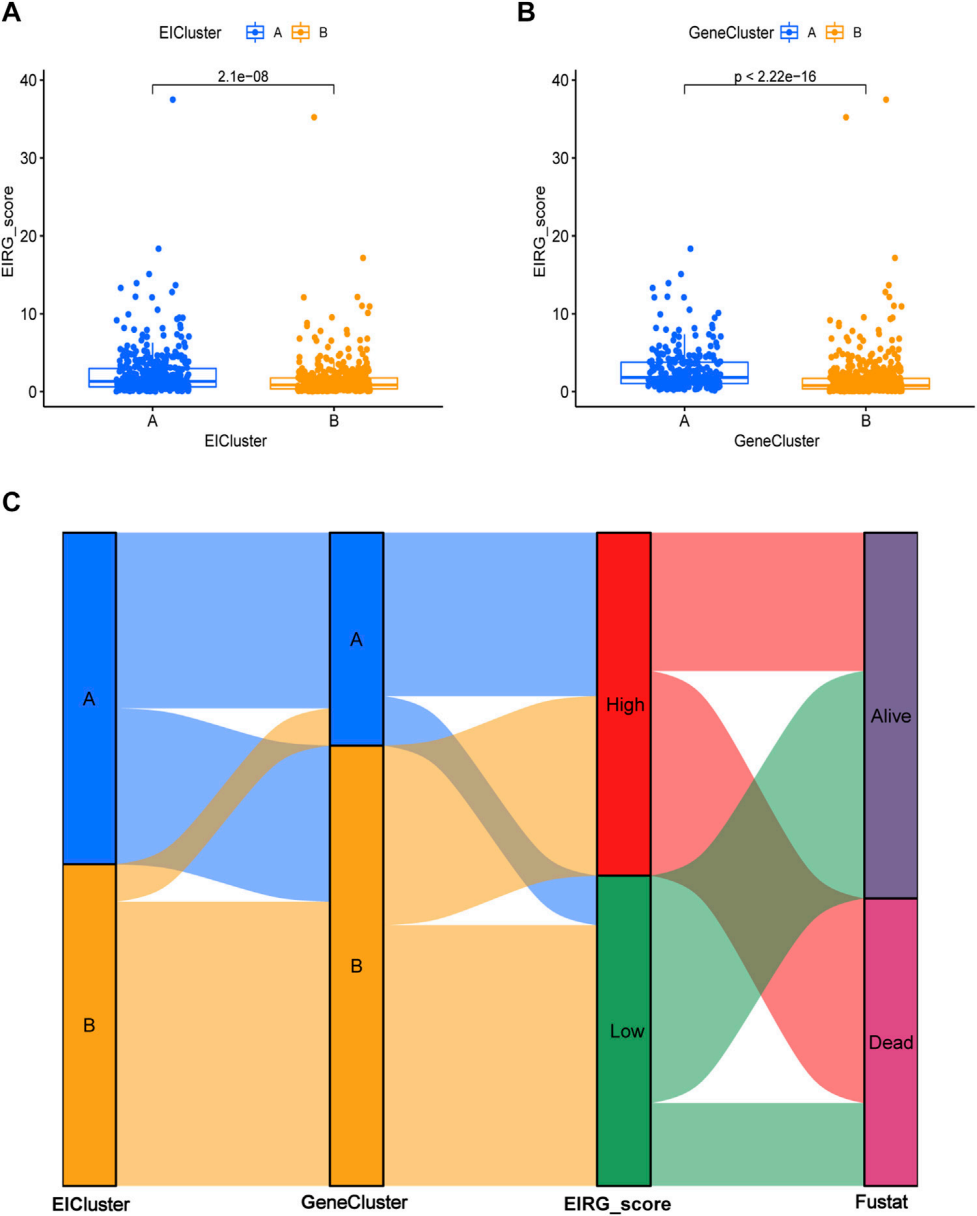
Distribution of EIRG_score. **(A)** differences in EIRG_score between different molecular subtypes. **(B)** differences in EIRG_score between different gene subtypes. **(C)** alluvial diagram of the distribution of different EIRG_score and survival outcome subtypes.

The EIRG_score was constructed by molecular subtyping of prognostic DEGs, and 30 genes were identified using Lasso regression analysis (Supplementary Figure S2) We then performed a multivariate Cox regression analysis on the 30 prognosis-related genes and finally obtained 18 hub genes for the construction of the EIRG_score model. The formula for the EIRG_score is as follows: The EIRG_score = (0.1921 × AKR1B1 exp.) + (−0.5852 × TRIM69exp.) + (0.1580 × FSTL3 exp.) + (0.3813 × PRDM6 exp.) + (0.6983 × SLC39A4 exp.) + (0.2299 × SENP7 exp.) + (0.2981 × DDIT4 exp.) + (0.5647 × MAN2A1 exp.) + (0.4436 × GLP2R exp.) + (0.2084 × EDN1 exp.) + (−0.3576 × EAF2 exp.) + (−0.3131 × FDX1 exp.) + (0.3803 × CNGA3 exp.) + (−0.4340 × ADAT3 exp.) + (−0.6221 × SH3BP2 exp.) + (0.8502 × S100Z exp.) + (−0.3144 × TBX3 exp.) + (−0.2143 × FRMD3 exp.). We divided the patients into training cohort (n = 402) and test cohort (n = 402) by using the “caret” package. We divided the patients into high- and low-risk groups using the median of EIRG_score in the training cohort. Detailed clinical data are available in Supplementary Table S3. The distribution of EIRG_score with EIRG molecular subtypes and GeneCluster is shown in Figure 6.

**FIGURE 7:**
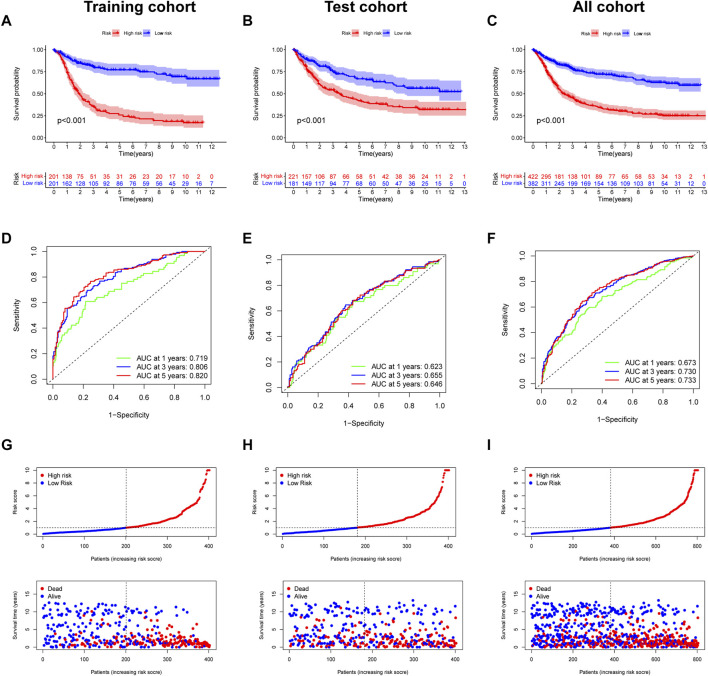
Prognostic assessment of EIRG_score. **(A)** Kaplan–Meier (KM) survival analysis of training cohort, **(B)** test cohort, **(C)** all cohorts. **(C)** receiver operating characteristic (ROC) curve analysis of training cohort, **(D)** test cohort, and **(E)** all cohorts. **(F)** Risk score distribution and survival scatter plot of **(G)** training cohort, **(H)** test cohort, and **(I)** all cohorts.

The survival curve indicated that in all groups, the high-risk group had a worse prognosis than the low-risk group (*p* < 0.001) (Figures 7A–C). In addition, the predicted 1-, 3-, and 5-year survival AUC values for EIRG_score were 0.719, 0.806, and 0.820 in the training cohort and 0.673, 0.730, and 0.733 in the all cohort, respectively (Figures 7D–F). The risk curve of EIRG_score shows that the score is negatively correlated with prognosis.

We downloaded the GSE62254 database (n = 300) as an external validation and calculated the score using the formula of EIRG_score from the training cohort. The patients were divided into two groups of high and low risks according to the median, and the survival analysis showed that the prognosis of the high-risk group was worse than that of the low-risk group (Supplementary Figure S3A). Using ROC curve analysis, the AUC values of EIRG_score for predicting 1-, 3-, and 5-year survival were 0.627, 0.687, and 0.651, respectively (Supplementary Figure S3B). The results indicated that EIRG_score had a positive effect in predicting the survival of GC patients.

### Construction and validation of a nomogram

**FIGURE 8:**
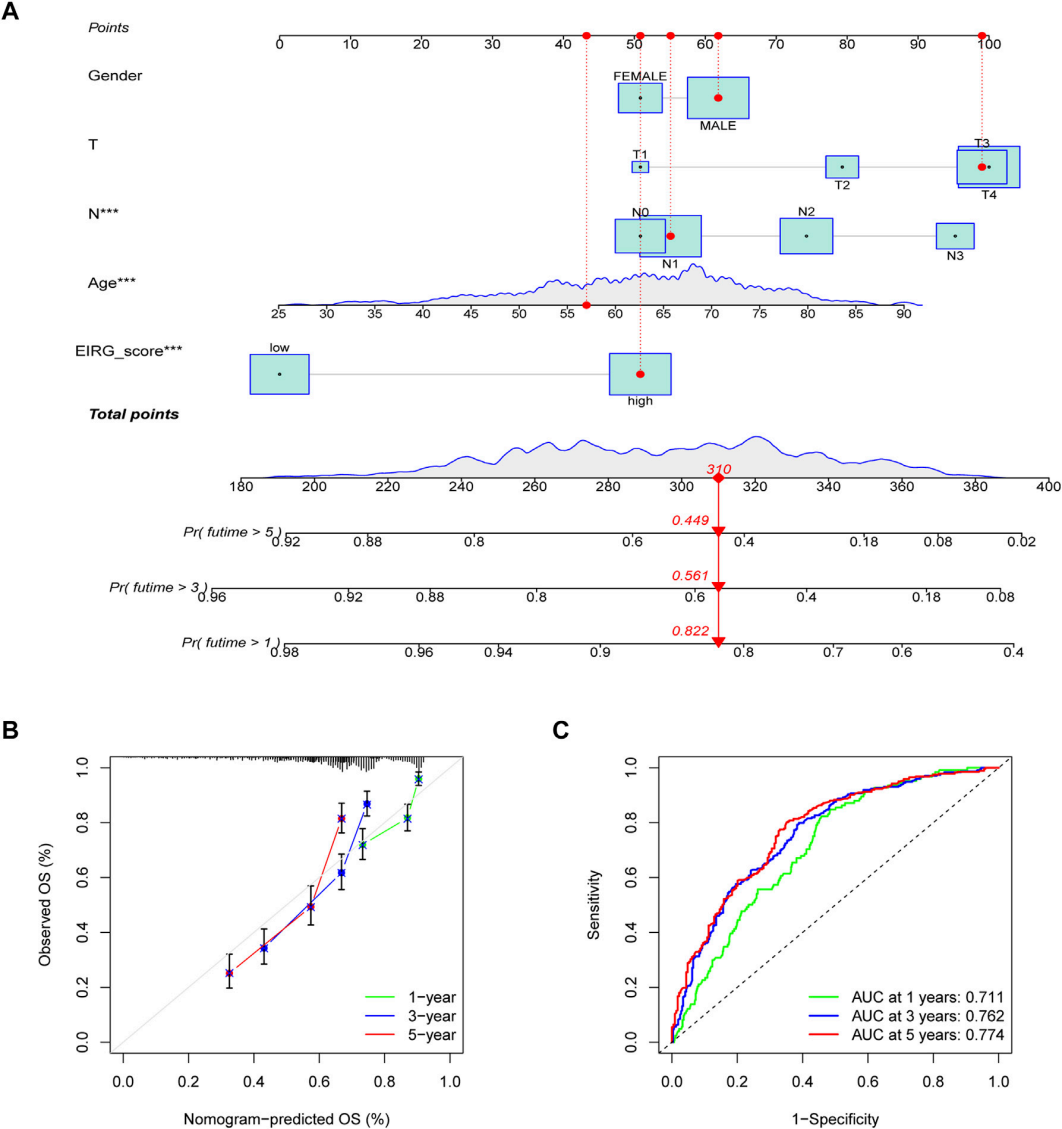
Construction and validation of nomogram. **(A)** nomogram for predicting 1-, 3-, and 5-year OS in patients with colorectal cancer in the training group. **(B)** calibration curves with a nomogram. **(C)** ROC curve analysis of the nomogram.

To more conveniently predict the prognosis of GC patients, we constructed a nomogram based on EIRG_score and clinicopathological characteristics (age, T-stage, N-stage, etc.) to predict the 1-, 3-, and 5-year OS rate of GC patients (Figure 8A). The calibration curves showed that the actual observed results were well consistent with the predicted results (Figure 8B). The ROC curve showed that the AUC values of the 1-, 3-, and 5-year OS of the nomogram were 0.711,0.762, and 0.774, respectively (Figure 8C), which indicated that the predictive efficacy of the nomogram was satisfactory.

### Immune infiltration analysis of EIRG_score

**FIGURE 9:**
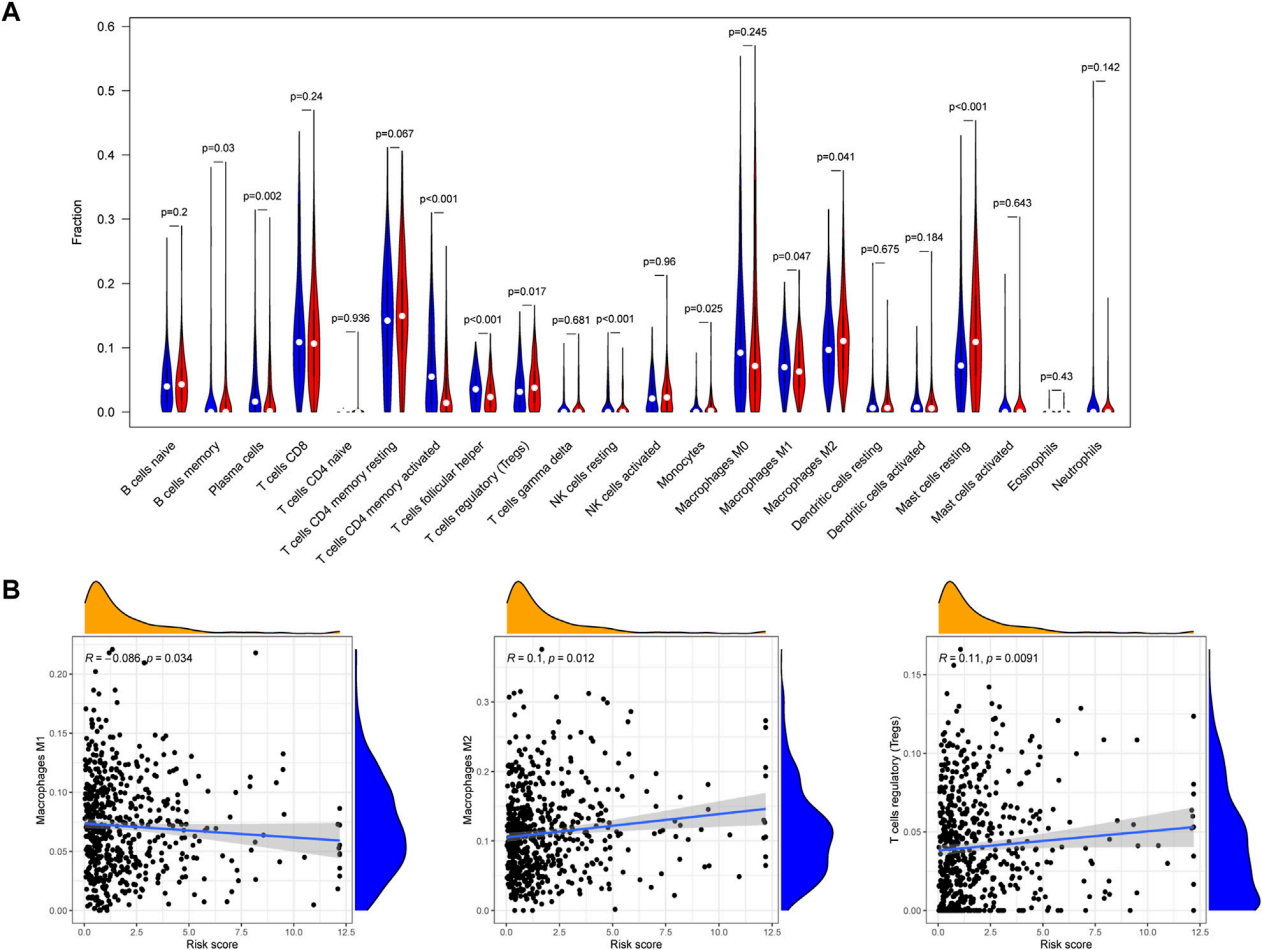
Immune infiltration analysis of EIRG_score. **(A)** abundance of 22 types of infiltrating immune cells in EIRG_score. Red represents the high EIRG_score group and blue represents the low EIRG_score group. **(B)** correlation analysis of EIRG_score with M1 macrophages, M2 macrophages, and Tregs. Correlation analysis using the Spearman correlation test.

We analyzed the relationship between EIRG_score and 22 immune cell infiltrations by the cibersort algorithm. The results showed that the high EIRG_score group had a higher abundance in Tregs, M2 macrophages, mast cells resting, and lower in M1 macrophages (Figure 9A). Then, we performed correlation analysis and EIRG_score was positively correlated with Tregs and M2 macrophages, which promote immunosuppression, and negatively correlated with M1 macrophages, which inhibit tumor progression (Figure 9B). It can be seen that the EIRG_score correlates with the immunosuppressive microenvironment.

### Correlation of EIRG_score with mutations

**FIGURE 10:**
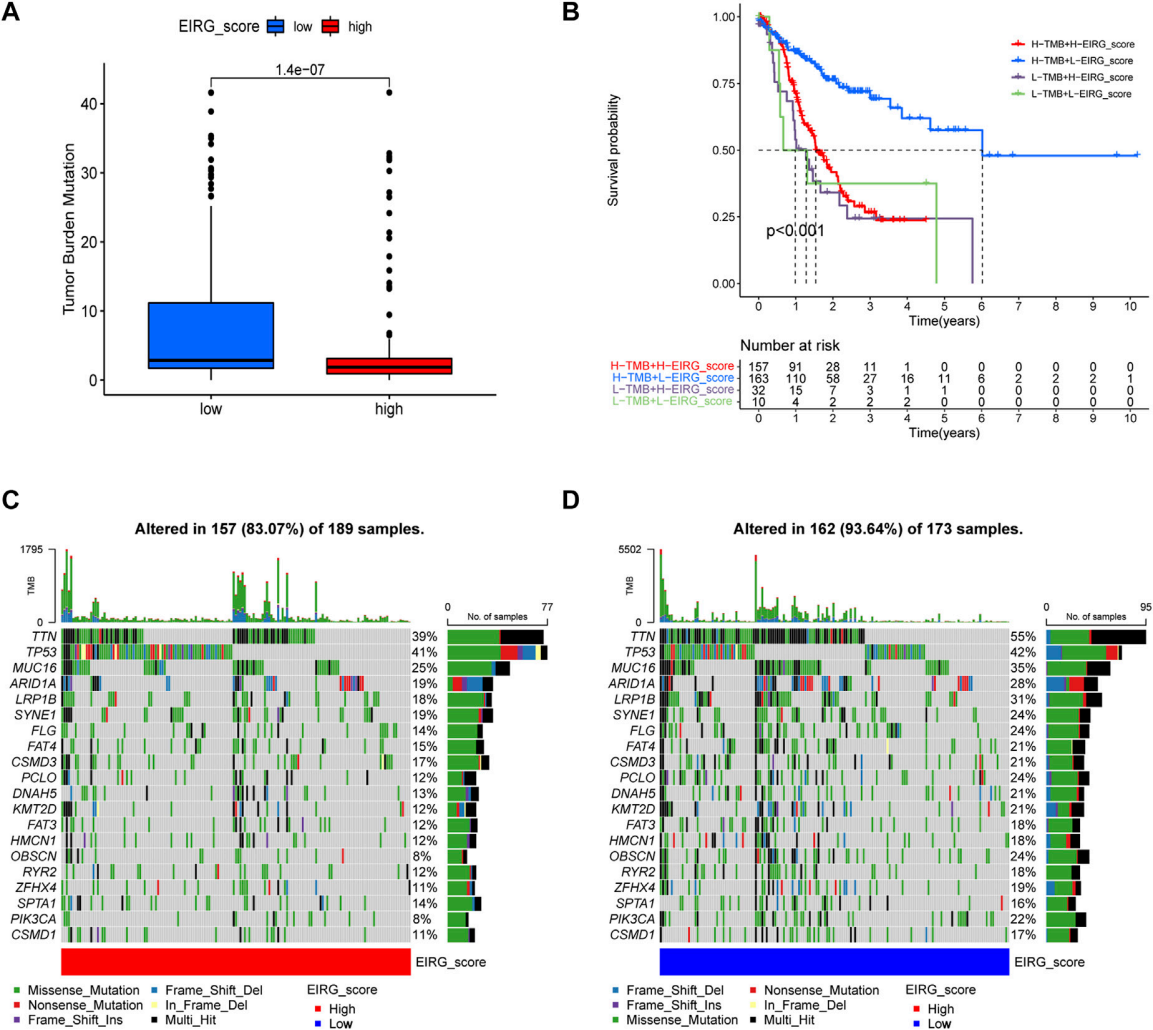
Mutation analysis of EIRG_score. **(A)** tumor mutational burden (TMB) of different EIRG_score groups. **(B)** KM survival analysis of EIRG_score and TMB. *p*-values are calculated using the log-rank test. Correlation of mutations between **(C)** high- and **(D)** low-risk group. Each column represents an individual patient. The numbers on the right indicate the mutation frequency of each regulator. The bars on the right show the proportion of each mutation type.

Tumor mutational burden (TMB) is considered to be a biomarker to predict a good response to immunotherapy. Therefore, we studied the correlation between EIRG_score and TMB, and we found that TMB was lower in the high score group than in the low score group (Figure 10A). As shown in Figure 10B, the low EIRG_score + high TMB group had the best prognosis (*p* < 0.001), suggesting that EIRG_score may be negatively correlated with immunotherapy response. Furthermore, we performed somatic mutation analysis for the high and low score groups by the “maftools” package, and we found that the mutation frequency in the high EIRG_score group (83.07%) was lower than that in the low EIRG_score group (93.64%), and the top three mutated genes in both groups were TTN, TP53, and MUC16(Figures 10C–D).

### EIRG_score predicts immunotherapy response

**FIGURE 11:**
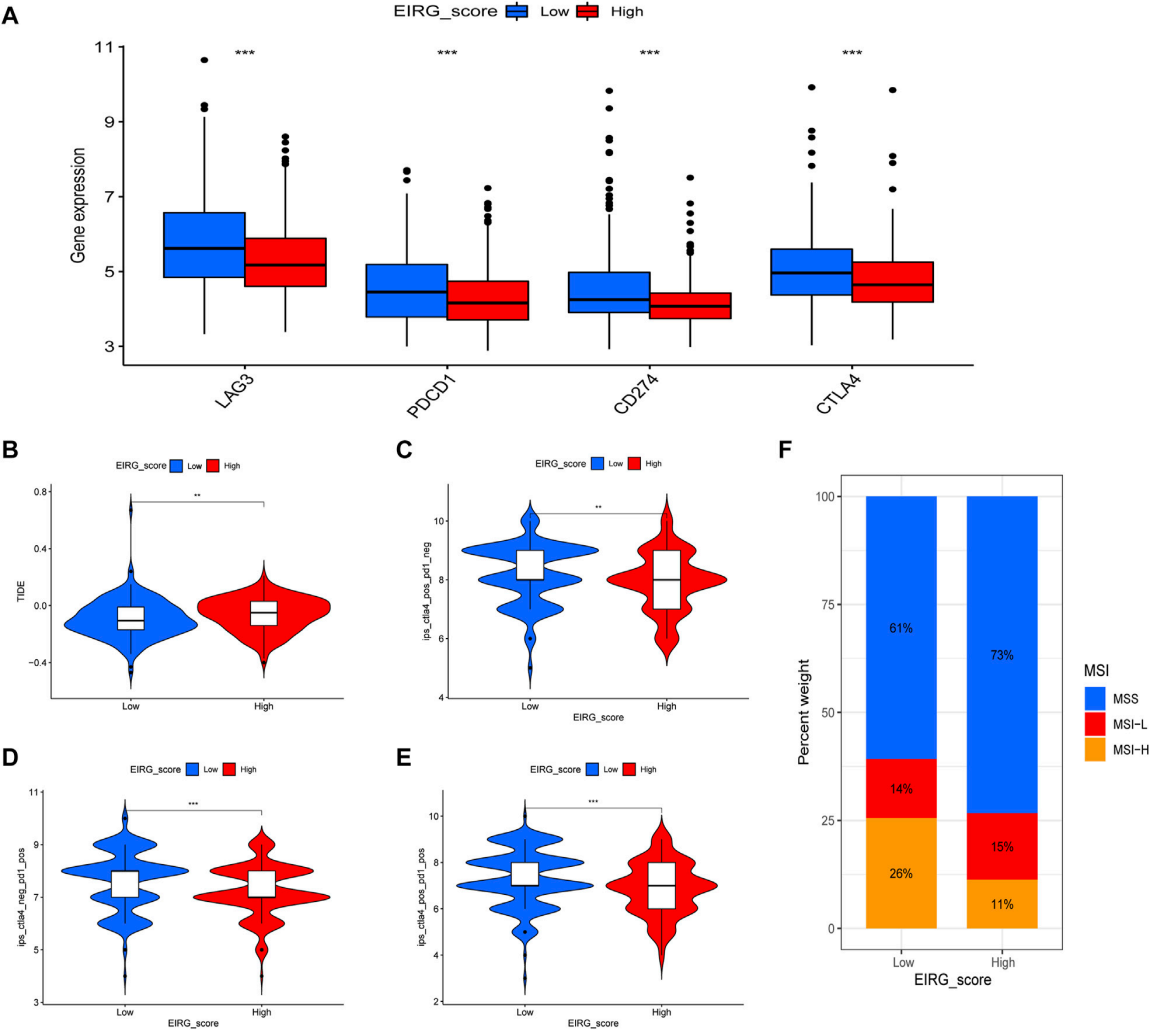
EIRG_score predicts immunotherapy response. **(A)** expression of immune checkpoint genes in high and low EIRG_score groups. **(B)** correlation of EIRG_score with tumor immune dysfunction and exclusion score. **(C–E)** correlation between EIRG_score and IPS. **(F)** correlation of EIRG_score with MSI. *p < 0.05, **p < 0.01, ***p < 0.001.

First, we analyzed the correlation between EIRG_score and immune checkpoint genes (CD274, CTLA4, LAG3, and PDCD1) and found that ICP genes expression was higher in the low EIRG_score group (Figure 11A). The results of the TIDE algorithm showed that the high EIRG_score group had a higher TIDE score, suggesting that the high EIRG_score may not respond well to ICB (Figure 11B). In addition, we included immunotherapy groups in the TCIA database for in-depth analysis, and the results showed that the low EIRG_score group had better treatment outcomes than the high EIRG_score group in the single anti-CALT4 treatment group, the single anti-PD1 treatment group, and the simultaneous anti-CALT4 and PD1 treatment group (Figures 11C–E). Moreover, the proportion of microsatellite instability-high (MSI-H) was lower in patients in the high EIRG_score group (11%) than in the low EIRG_score group (26%) (Figure 11F).

At last, we investigated the correlation between EIRG_score and chemotherapeutic drug sensitivity by using the “pRRophetic” package. It was found that the IC50 of chemotherapeutic drugs such as cyclopamine, gemcitabine, paclitaxel, and lenalidomide were higher in the high EIRG_score group than in the low EIRG_score group (Supplementary Figure S4), indicating that the high EIRG_score group may be resistant to these drugs.

### AKR1B1 affects GC cell proliferation and migration

**FIGURE 12:**
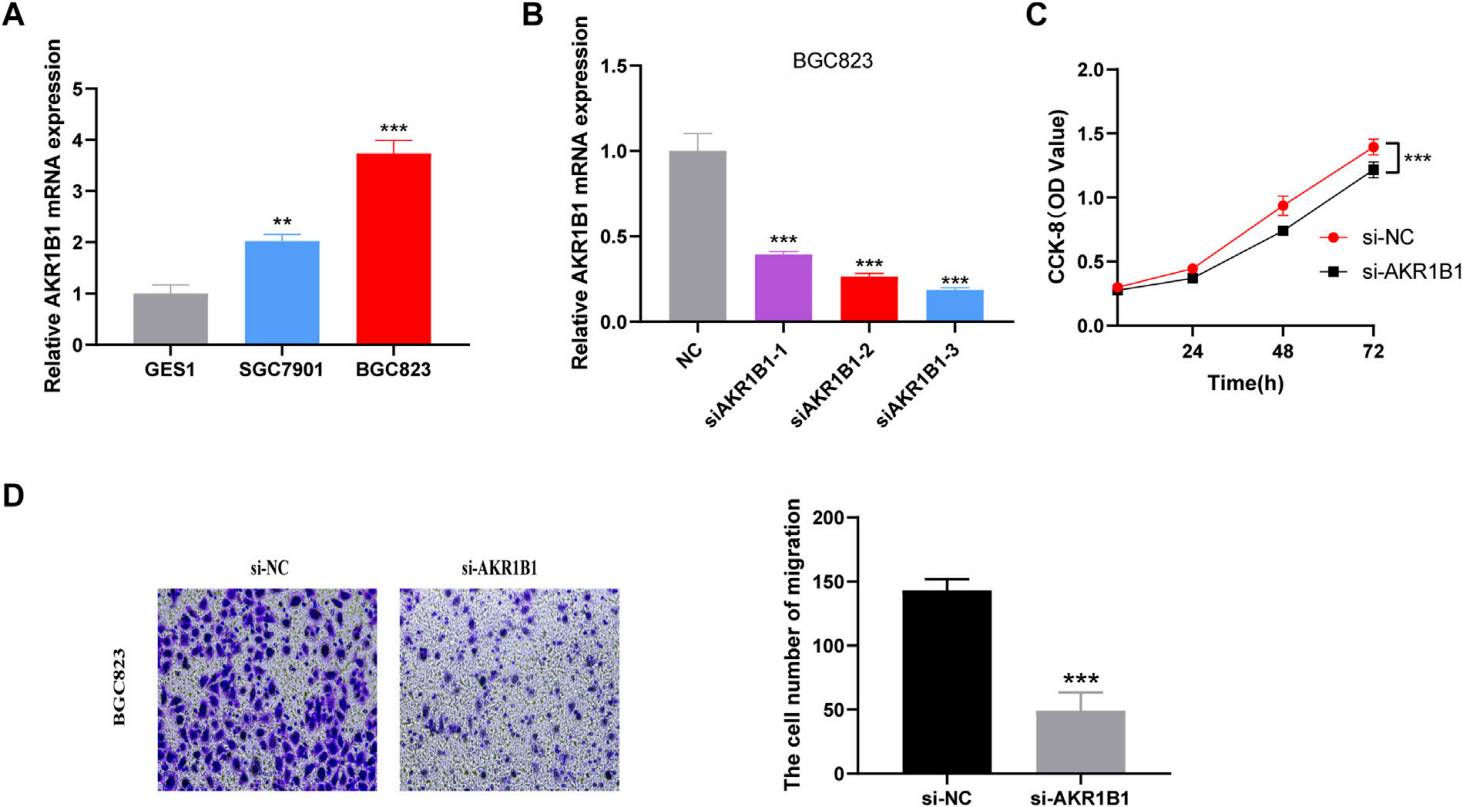
Validation of the biological function of AKR1B1 **(A)** quantitative polymerase chain reaction (qPCR) displayed upregulation of AKR1B1 in gastric cancer (GC) cells compared to normal cell line (GES1). Results represent mean ± SD; *n* = 3. ***p < 0.001; two-tailed *t*-test. **(B)** validation of knockdown efficiency by qPCR. Results represent mean ± SD. *n* = 3. ***p < 0.001; two-tailed *t*-test. **(C)** AKR1B1 siRNA displayed reduced proliferation of BGC823 cell. Results represent mean ± SD; *n* = 3; ***p < 0.001; two-tailed *t*-test. **(D)** AKR1B1 knockdown inhibited migration of BGC823 cells. Results represent mean ± SD; *n* = 3. **p < 0.01; ***p < 0.001; two-tailed *t*-test.

We explored the biological function of EIRG_score by *in vitro* experiments, and we selected AKR1B1, which has barely been studied in GC, for our study. The UALCAN database (Chandrashekar et al., 2017) and IHC results revealed that AKR1B1 was highly expressed in GC tissues and associated with poor prognosis (Supplementary Figure S5). qRT-PCR results showed that AKR1B1 was highly expressed in GC cell lines, which was consistent with the database results (Figure 12A). Then, we performed AKR1B1 knockdown by transfection of siRNA (Figure 12B), and we discovered through CCK-8 and transwell assays that knockdown of AKR1B1 significantly inhibited GC cell proliferation and migration (Figures 12C,D). It indicates that AKR1B1 plays a procancer role in GC.

## Discussion

Multiple factors are influencing GC development and progression, for example, EMT can promote GC invasion, metastasis, and resistance to chemotherapy. Moreover, the microenvironment of GC can affect tumor progression. Focusing on a single factor alone may not be sufficient to provide a comprehensive understanding of GC. We included the combination of EMT and the immune microenvironment in our study for the first time to explore the combined effects on GC prognosis and immunotherapy.

We collected 1184 EMT- and 1959 immune-related genes from databases and previous studies and identified DEGs through the TCGA database, obtaining 82 overlapping intersection genes as EIRGs. We classified GC patients into two molecular subtypes by EIRGs, and the prognosis of subtype A was worse compared with subtype B. Moreover, there were significant differences between the two subtypes in TME, with subtype A having a higher TME score than subtype B. EIRGs molecular subtypes are enriched in biological pathways such as EMT, IL6-JAK-STAT3 signaling, IL2-STAT5 signaling, and TGF-β signaling, and previous studies have shown that CAFs in GC cells enhance EMT by secreting IL-6 to activate the JAK2/STAT3 pathway in GC cells (Wu et al., 2017). MDSCs and Tregs are more abundant in subtype A than in subtype B. Tregs can suppress CD8^+^ T cell activation and also secrete IL-10 and TGF-β to inhibit tumor-specific T cell infiltration and function, thereby causing immunosuppression (Ahrends and Borst, 2018). MDSCs are immature immunosuppressive myeloid cells that can inhibit CD8^+^ T cell function through the expression of PD-L1 and CTLA-4 and can induce EMT (Oya et al., 2020), suggesting that subtype A has an immunosuppressive profile (cold tumors) and subtype B has an immune-activating profile (hot tumors).

We performed a differential analysis of the molecular subtypes of EIRG, resulting in two gene subtypes. Genotyping was significantly correlated with the prognosis and immune infiltration of GC. To better assess the prognosis and immunotherapeutic response of GC, we constructed an EIRG_score based on the differential genes of EIRGs molecular subtypes and explored its predictive ability. Compared with the low EIRG_score group, the high EIRG_score group had a worse prognosis, with subtype A, characterized by cold tumors, associated with a higher EIRG_score, and subtype B, characterized by hot tumors, associated with a lower EIRG_score.

Then, we performed an immune infiltration analysis of EIRG_score and found that Tregs in the high EIRG_score group had an increased abundance of M2 macrophage infiltration, whereas in the low EIRG_score group, M1 macrophage infiltration abundance was increased. The correlation results showed that EIRG_score was positively correlated with Tregs and M2 macrophages and negatively correlated with M1 macrophages. Macrophages are mainly divided into M1 and M2 types. M1 macrophages can kill tumors through both antibody-dependent cell–mediated cytotoxicity and direct-mediated cytotoxicity and therefore have tumor suppressive effects (Pan et al., 2020). By contrast, M2 macrophages can promote tumor proliferation, invasion, and angiogenesis and are associated with EMT, which can promote tumor metastasis and cause poor patient prognosis (Rihawi et al., 2021). This may explain the worse prognosis in the high EIRG_score group.

A significant part of immunotherapy is ICIs. However, the majority of patients receiving ICIs do not benefit from them (Sha et al., 2020). Therefore, we wanted to explore whether EIRG_score could be used as a biomarker to predict the efficacy of immunotherapy.

Because of PDL1 expression, TMB and MSI-H are considered to be biomarkers that can predict the efficacy of immunotherapy (Rizzo et al., 2021). Therefore, we explored the correlation between EIRG_score and ICP genes, TMB and MSI-H. We found that the low EIRG_score group had higher ICP gene expression levels, higher TMB, and higher MSI-H proportion than the high EIRG_score group. Moreover, we combined TMB and EIRG_score for prognostic analysis and found that TMB-high + low EIRG_score had the best prognosis. In addition, we calculated the TIDE score of GC using the TIDE algorithm, and the TIDE scores were higher in the high EIRG_score group, indicating that the high EIRG_score had a poorer response to immunotherapy; in IPS assessment, the IPS scores were higher in the low EIRG_score group in any treatment group, suggesting that the low EIRG_score responded better to immunotherapy. The above results suggest that EIRG_score can be used as a biomarker to identify and screen patients for immunotherapy, and the lower the EIRG_score value, the better the response of GC patients to immunotherapy.

At last, we examined the correlation between EIRG_score and chemotherapeutic drug sensitivity and showed that EIRG_score was positively correlated with the IC50 of several drugs, including paclitaxel. Paclitaxel is a first-line chemotherapy drug that exerts its anticancer effects by inhibiting cell cycle progression, and it was found that paclitaxel can inhibit Tregs, which can reverse immunosuppression (Zhu and Chen, 2019), suggesting that the low EIRG_score group may benefit from it. Previous studies have found that AKR1B1 plays a major role in tumor progression, and the mechanisms of action of AKR1B1 include participation in EMT and immune regulation. In addition, AKR1B1 has regulatory effects on the synthesis of reactive oxygen species and prostaglandins (Khayami et al., 2020). Moreover, AKR1B1 expression was higher in GC patients with poorer OS prognosis, suggesting that AKR1B1 is associated with poorer prognosis in GC (Xiong, 2021). In the present study, we found that AKR1B1 could promote GC cell proliferation and migration, which is consistent with the results of previous studies.

There are still some limitations in our study; on the one hand, it is only a retrospective study of data from public databases, and more prospective and multicenter clinical studies are needed to further confirm our results. On the other hand, more *in vivo* and *in vitro* experiments are needed to investigate the molecular mechanisms underlying the effects of EIRGs.

## Conclusion

For the first time, we included EMT- and immune-related genes jointly in our study, comprehensively analyzed the role of EIRGs in GC, and constructed the EIRG_score model, which can be used as a biomarker for predicting mutation, prognosis, and response to immunotherapy, providing a new thought for precise treatment of GC.

## Data Availability

The datasets presented in this study can be found in online repositories. The names of the repository/repositories and accession number(s) can be found in the article/Supplementary Material.
