# Supplementary material for: Identification of molecular patterns and prognostic models of epithelial–mesenchymal transition- and immune-combined index in the gastric cancer
Source: Front Pharmacol. 2022 Aug 9;13:958070. doi: 10.3389/fphar.2022.958070 (PMC9397546; doi:10.3389/fphar.2022.958070)
Supplement: Supplementary file 1 [file Table1.DOCX]

# Supplementary Table S1

## Cell Transfection

Tumor cells were seeded in six-well plates, and when cell density in each well reached 70%, siRNA transfection was performed by Lipofectamine 3000 reagent (Invitrogen, USA). AKR1B1 siRNA sequences and negative control (NC) were obtained from OBiO (Shanghai, China). Transfection was carried out with Lip3000(OBiO, China). The sequences of siRNAs were listed: *AKR1B1*-si1,5′-GGUGAAAGCUAUUGGCAUCTT-3′; *AKR1B1*-si2,5′-AGGAGAAGUUAAUCCAGUATT-3′; *AKR1B1*-si3,5′-GAACUGAGCAGCCAGGAUATT-3′.

and NC, 5′-UUCUCCGAACGUGUCACGUTT-3′.

## Real-Time Quantitative PCR

The RNA of the cells was extracted with TRIzol solution (TaKaRa, Japan), then the concentration of RNA in each group was determined. The cDNA was obtained according to the reverse transcription cDNA synthesis kit (Thermo, USA). The relative expression of genes was finally calculated according to the SYBR Green PCR kit (Thermo, USA) and the 2^−ΔΔCt^ method.

All primers were listed as follows: AKR1B1, Forward:CATGCAGAGGAACTTGGTGGTGAT, Reverse:TGTTGTAGCTGAGTAAGGTGGTCATATC.GAPDH,Forward:TGCACCACCAACTGCTTAGC,and Reverse:GCGCCCAATACGACCAAATC.

## CCK8 Viability Assay

Cells (2,000 cells/well) were seeded in 96-well plates, after overnight attachment, the medium was changed to 100 μl FBS-free medium with 10% CCK8 (MCE, USA) in each well and incubated for 2 h at 37°C, then the OD values at 450 nm were detected through microplate reader (BioTek, USA). These steps were repeated at 4, 24, 48, and 72 h.

## Transwell For Migration Assay

For transwell migration assay, 6 × 10^4^ cells were seeded on the upper transwell chambers in 200 μl serum-free culture medium, and 700 μl medium containing 10% FBS was added to the lower chambers. After 24 h incubation, the cells that migrated through membranes were fixed with methanol, stained with 1% crystal violet and counted under light microscope (200×).
