# Supplementary material for: Identification of molecular patterns and prognostic models of epithelial–mesenchymal transition- and immune-combined index in the gastric cancer
Source: Front Pharmacol. 2022 Aug 9;13:958070. doi: 10.3389/fphar.2022.958070 (PMC9397546; doi:10.3389/fphar.2022.958070)
Supplement: Supplementary file 2 [file Table2.DOCX]

**Table S2: EMT-related genes, immune-related genes and EMT-and immune-related genes.**

| EMT-related genes | Immune-related genes | DEGs | EIRGs |
| --- | --- | --- | --- |
| TGFB1 | CALB2 | MTHFD1L | ENG |
| SNAI1 | IL32 | NFE2L3 | ETS1 |
| ZEB1 | ST3GAL6 | RCC2 | ST6GAL1 |
| CDH1 | KRT1 | ECT2 | CXCL9 |
| TWIST1 | ADTRP | ZWILCH | LRP6 |
| CTNNB1 | PTPRB | RUNX1 | GREM1 |
| STAT3 | KCNE4 | UTP18 | PDGFB |
| SNAI2 | FCGR2B | CEP55 | TNFRSF11A |
| HIF1A | EVPL | SOX9 | CCL3 |
| AKT1 | XCL1 | ADPGK | OSM |
| MIR200C | NTM | DKC1 | TNFSF15 |
| ZEB2 | IFNA1 | CLSPN | STAT1 |
| SMAD3 | CD1C | TPX2 | NR5A2 |
| EGFR | IL12A | MARCKSL1 | MET |
| MIR200B | CD79B | NOP2 | RUNX3 |
| SMAD2 | VNN2 | IQGAP3 | LIF |
| MIR200A | RCN3 | MKI67 | HPGD |
| NOTCH1 | BTLA | E2F3 | CXCL12 |
| MIR21 | DOCK10 | DTL | HAVCR2 |
| ILK | CRYBB1 | STIL | SPARC |
| GSK3B | SLC18A2 | CDK1 | KIT |
| NFKB1 | CXCR1 | CENPO | FSCN1 |
| IL6 | MCOLN2 | KIF18B | GJB2 |
| HMGA2 | GPR35 | ANLN | EPCAM |
| CXCR4 | S100A12 | BID | ISG15 |
| TP53 | LYZ | KIF11 | AGTR1 |
| EGF | WAS | TEAD4 | CXCL8 |
| MTDH | FBXO6 | KIF23 | NOTCH4 |
| MAPK1 | CDH6 | ARHGAP11A | SERPINE1 |
| CD44 | SPI1 | OSBPL3 | AQP9 |
| BMI1 | PDIA3 | BUB1 | HSPA5 |
| PTEN | CLEC5A | TXNDC12 | LAMC2 |
| MTOR | FCGRT | MTHFD2 | NRP1 |
| VIM | CEACAM19 | HELLS | FN1 |
| RAC1 | SLA | KNOP1 | PTPN6 |
| MMP9 | IL10RA | KIF20A | MMP1 |
| MMP2 | NR5A2 | NUSAP1 | PRRX1 |
| GLI1 | ANTXR2 | RAD51AP1 | IRF8 |
| MET | EOMES | DBF4 | CCL21 |
| SMAD4 | FCRL1 | TIMELESS | GATA3 |
| KLF4 | GBP5 | FANCI | CXCR4 |
| WNT5A | NCF1B | ENC1 | MMP3 |
| MYC | LSAMP | KPNA2 | F2R |
| SMAD7 | GPR171 | PACC1 | IL11 |
| SIRT1 | LIMD2 | CBFB | LYN |
| CTGF | LRRC32 | BUB1B | JAK3 |
| CCR7 | EIF2AK2 | TRAF2 | BATF2 |
| PROM1 | ZDHHC20 | COLGALT1 | S100A8 |
| TGFB2 | STAT1 | PNO1 | PLXND1 |
| PIK3CA | IL18BP | PIGW | COL8A1 |
| MIR34A | HSPA1L | PRIM2 | VEGFA |
| RHOA | CDH11 | MELK | TGM2 |
| AR | ATP2A3 | SLC12A8 | C5AR1 |
| CD274 | CTHRC1 | CENPF | MMP14 |
| FOXM1 | TREML1 | EFTUD2 | HSP90AA1 |
| YAP1 | PLEKHM3 | FOXM1 | TNFSF11 |
| SRC | DACT1 | ATAD2 | CD36 |
| MIR30A | CD96 | SLC38A7 | CXCL13 |
| MIR205 | LY86 | RIPK2 | KCNN4 |
| MALAT1 | HLA-DRB6 | KIF14 | CDH11 |
| JAK2 | FUT7 | EML4 | NMI |
| WWTR1 | BMP7 | PTBP1 | LGALS9 |
| CDH2 | ACAP1 | UBE2T | IL23A |
| TNF | PGLYRP4 | NEK2 | CCL18 |
| CXCL12 | IL9 | POLR1B | ADAM12 |
| RELA | P2RY12 | WDR43 | PDGFRB |
| MAPK3 | MEF2B | CBX3 | CTHRC1 |
| MIR221 | FPR3 | CIP2A | PSME3 |
| MIR145 | LRP1 | HJURP | ACTA2 |
| CXCL8 | DDR2 | CENPL | CXCL5 |
| HGF | KLK7 | CNOT9 | CSF2 |
| FN1 | ADAM8 | SMYD5 | PKP3 |
| TWIST2 | CLMP | MYBL2 | POSTN |
| TP63 | TREM1 | EXO1 | CCL20 |
| NANOG | IFITM1 | TROAP | VCAN |
| VEGFA | GLT8D2 | DPH2 | LOXL2 |
| SP1 | IL20 | PRR11 | ITGB1 |
| S100A4 | GALM | DLGAP5 | MMP9 |
| PTK2 | ATP10A | QTRT2 | CTSL |
| MAPK7 | PTPRM | ZWINT | PROM1 |
| MUC1 | RDH12 | RAD51 | CXCL16 |
| HMGB1 | PSTPIP1 | FAM91A1 | EPS8 |
| EZH2 | RNF125 | CASP2 |  |
| ESR1 | PLEKHO2 | GNL2 |  |
| HOTAIR | TFEC | EIF2AK1 |  |
| CAV1 | VCAM1 | G3BP1 |  |
| SOX2 | CASS4 | CDCA8 |  |
| BMP7 | RELB | TOP2A |  |
| BMP4 | COTL1 | NUF2 |  |
| SHH | LRCH2 | SKA3 |  |
| PTGS2 | ZKSCAN8 | PUS1 |  |
| POU5F1 | ACVR2B | WDR89 |  |
| PRRX1 | TRABD2A | CLN6 |  |
| KRAS | CD200 | NDC80 |  |
| ID1 | KIF21B | TGIF1 |  |
| H19 | CISH | TRIP13 |  |
| FGFR1 | TNFSF12-TNFSF13 | ZNF267 |  |
| POSTN | TNFRSF17 | RFWD3 |  |
| FOXQ1 | USHBP1 | HMMR |  |
| MTA1 | PROCR | PRPF40A |  |
| GRHL2 | TNFSF12 | SART3 |  |
| TRPS1 | GYPC | APMAP |  |
| ADAM17 | SP110 | KIF4A |  |
| T | CNTFR | EZH2 |  |
| AURKA | PCOLCE | NUP85 |  |
| BMP2 | THPO | UTP25 |  |
| CCL18 | ABCD2 | DDX21 |  |
| CCL2 | TYMP | CDCA5 |  |
| ROCK1 | MMRN2 | NOL10 |  |
| MIR429 | PML | ASPM |  |
| ESRP1 | CD248 | SGO1 |  |
| PPARG | P2RX4 | UBE2C |  |
| NTRK2 | FIBIN | TPM3 |  |
| EPCAM | FBP1 | ALG3 |  |
| MIR93 | TNFRSF14 | CYRIB |  |
| MIR204 | CD1A | RFC3 |  |
| MIR203A | NRROS | PRC1 |  |
| MIR186 | ADAT1 | KIF2C |  |
| MIR155 | CCL28 | GTSE1 |  |
| MIR148A | TSPAN32 | LIMK1 |  |
| LOXL2 | TTC37 | ATIC |  |
| LGALS1 | N4BP2L1 | GTPBP4 |  |
| JUN | LAMP5 | PLK1 |  |
| ITGB1 | AMIGO3 | DNMT1 |  |
| HDAC1 | IFIT5 | ZNF281 |  |
| GREM1 | FAM92B | E2F1 |  |
| KDM1A | HSPA5 | GINS1 |  |
| AGER | MLPH | UHRF1 |  |
| NUMB | CXCL9 | CCNB2 |  |
| WNT1 | HLA-F | KIF18A |  |
| EZR | LGALS2 | ASF1B |  |
| TLR4 | NFKBIE | KNTC1 |  |
| TCF3 | ADAP2 | LSG1 |  |
| STK11 | ARHGAP31 | RAD51D |  |
| UCA1 | FCRL2 | PSMD14 |  |
| CCL21 | USP12 | NDC1 |  |
| CCL20 | PCDH12 | SHCBP1 |  |
| LEF1 | RGL1 | SPAG5 |  |
| PAX2 | IRF8 | NUDCD1 |  |
| DDR2 | TGM1 | RUVBL1 |  |
| YBX1 | IGSF10 | DCBLD1 |  |
| MCAM | SUSD3 | UCK2 |  |
| MIR26B | IL9R | DDX56 |  |
| MIR23A | SERPINF1 | RFC4 |  |
| MIR214 | HS3ST2 | CHEK1 |  |
| MIR19A | FLT3LG | CENPK |  |
| MIR150 | FBLN5 | CKAP2L |  |
| MIR141 | MPL | DDX31 |  |
| MIR124-1 | ZAP70 | CTHRC1 |  |
| MIR101-1 | ENO3 | LRFN4 |  |
| LOX | LAMTOR2 | DSCC1 |  |
| LCN2 | FYN | LRRC8D |  |
| IL17A | BIRC6 | DCLRE1B |  |
| IL1B | HLA-DPB2 | SPDL1 |  |
| IGF1R | GBP2 | HNRNPA2B1 |  |
| IGF1 | ANXA1 | NAT10 |  |
| HSF1 | SSTR3 | TRIM59 |  |
| HNF4A | IL11RA | TMPO |  |
| FOXA1 | DSP | TTYH3 |  |
| FOXC2 | SPRR2E | PKMYT1 |  |
| ERBB2 | ARHGAP22 | NOL11 |  |
| EGR1 | BCL6B | PCLAF |  |
| KLF8 | PCYOX1L | CDC45 |  |
| NES | RBM38 | PPAT |  |
| LINC-ROR | LILRA5 | CCNB1 |  |
| MIR300 | LYPD5 | INHBA |  |
| NUAK1 | PILRA | NUDT5 |  |
| DCLK1 | CCL11 | HNRNPM |  |
| CLDN1 | IL12RB2 | TNFRSF10B |  |
| RUNX2 | NAPSB | WDR75 |  |
| TET1 | SMAP2 | NCAPH |  |
| TP73 | LTB | DDX18 |  |
| THBD | FAM83A | DHX37 |  |
| TGM2 | NRP1 | SCAMP3 |  |
| TGFBR3 | FLT3 | DCAF13 |  |
| TGFBR2 | RAD54L2 | PDCD11 |  |
| TGFB3 | NLRC3 | SPC25 |  |
| STIM1 | FYB1 | GPATCH4 |  |
| SRF | XPNPEP2 | MTFR2 |  |
| BRCA1 | DDX58 | KIF20B |  |
| SPARC | ECSCR | UTP6 |  |
| SOX9 | CCR9 | FANCD2 |  |
| SOX4 | LAIR1 | MRPL17 |  |
| FSCN1 | COL6A2 | DEPDC1B |  |
| SIX1 | NFAM1 | SOX4 |  |
| SDC1 | IL20RA | MRPL3 |  |
| SATB1 | ERAP2 | CCT6A |  |
| BCL2L1 | JAK2 | UBAP2L |  |
| OVOL2 | TNFRSF13B | SRSF10 |  |
| MKL1 | SIT1 | SERPINH1 |  |
| NDRG2 | IFNG | ACTL6A |  |
| EIF5A2 | CD300E | MRM2 |  |
| PARD3 | NHLRC2 | FHOD1 |  |
| PRKCI | N4BP2 | TUBB |  |
| AXL | PVR | CKS2 |  |
| FBXW7 | EBI3 | BUD23 |  |
| TRIM33 | BTN3A2 | RRM2 |  |
| PAK1 | PTCRA | NCAPG |  |
| OSM | DNAH8 | GARS1 |  |
| ROR2 | C3 | C1orf112 |  |
| NOTCH2 | TRIM22 | HSP90AA1 |  |
| MSX2 | TMEM26 | URB2 |  |
| MMP14 | RHOH | METTL1 |  |
| MIR9-1 | CDK15 | PARPBP |  |
| MIR30C2 | ANKRD22 | PNPT1 |  |
| MIR30C1 | GGT5 | PGM2L1 |  |
| MIR29C | HEPH | LMNB1 |  |
| MIR29B1 | PLA1A | HSPD1 |  |
| MIR26A1 | CD33 | DCAF7 |  |
| MIR137 | SCML4 | ZNF367 |  |
| MIR130B | NOD2 | KAT2A |  |
| MIR10B | IFI44 | POLE2 |  |
| MIR106B | LOXL3 | TSN |  |
| MIR106A | IVL | SLC52A2 |  |
| MIRLET7A1 | CLECL1 | ESM1 |  |
| LEP | CCL8 | INTS7 |  |
| L1CAM | PLXNC1 | HMGB3 |  |
| RHOC | TNFRSF10C | SRSF1 |  |
| CYR61 | TMEM170B | PRRC2C |  |
| BIRC5 | LINC00426 | PTRH2 |  |
| HSPA5 | ADCY4 | CCNF |  |
| HOXA13 | NFYB | RRP12 |  |
| ANXA2 | MRGPRF | CSTF3 |  |
| PDCD4 | DACT3 | NOP58 |  |
| GATA3 | CALHM6 | TWNK |  |
| ALK | JUP | XPO1 |  |
| FLOT2 | MZB1 | XRCC2 |  |
| FOXO3 | PLXNA4 | ATP11A |  |
| FOXC1 | CHST2 | SLC30A7 |  |
| FGFR2 | KLRB1 | FEN1 |  |
| FGF2 | FNBP1 | INTS14 |  |
| AKT2 | EMILIN2 | NOP56 |  |
| EPAS1 | FABP3 | SLC5A6 |  |
| DNMT1 | LILRB1 | PLK4 |  |
| DAB2 | FGD2 | MCM2 |  |
| CUX1 | DOCK2 | BLM |  |
| CTNND1 | SLC1A7 | NUP62 |  |
| CTBP1 | ABI3BP | KIFC1 |  |
| MAPK14 | HYDIN | SINHCAF |  |
| NLRP3 | IL1RN | DONSON |  |
| CYTOR | RUBCNL | MASTL |  |
| AGR2 | PSMD1 | PAICS |  |
| NDRG1 | PARP14 | RECQL4 |  |
| CDKN1B | LINC00654 | MCM10 |  |
| CD24 | CSF3R | TMEM138 |  |
| TNFSF15 | WFDC12 | AURKA |  |
| GDF15 | CLIC5 | CENPA |  |
| TP53INP1 | TNFSF13 | ILF2 |  |
| TM4SF5 | CD300LB | MAD2L1 |  |
| WNT3A | IL24 | RCC1 |  |
| WISP2 | NTNG2 | TARDBP |  |
| NRP1 | NBEA | SRPK1 |  |
| ACTL6A | DCSTAMP | MCM4 |  |
| CUL4A | KIAA1755 | SLC39A10 |  |
| FZD7 | RASSF3 | ENTR1 |  |
| FOSL1 | TNFSF4 | BUB3 |  |
| ADAM12 | ARHGAP6 | CDCA2 |  |
| PDGFD | PSMC2 | PUS7 |  |
| VASH2 | CARD8 | TACC3 |  |
| TBL1XR1 | LAMC2 | EPCAM |  |
| TNFAIP8L2 | HAMP | TOMM34 |  |
| YWHAG | LAT | NIFK |  |
| YY1 | BIN2 | LIG1 |  |
| XBP1 | ATP8A1 | AVL9 |  |
| WT1 | CD163L1 | ABCE1 |  |
| VDR | CXCL2 | DDX55 |  |
| CCR2 | CD69 | WDHD1 |  |
| MIR655 | MGAT4A | NAA25 |  |
| NR2C2 | SLCO5A1 | QSOX2 |  |
| TIMP2 | GCSAML | CPSF3 |  |
| TGFBR1 | SPARCL1 | POC5 |  |
| TGFA | CD74 | PPM1G |  |
| TERT | PSMD3 | ATAD5 |  |
| MIR630 | ALDH1A1 | DSN1 |  |
| MIR590 | CD244 | CDC7 |  |
| TCF4 | FMOD | CAD |  |
| BSG | SLC15A3 | GDF15 |  |
| SPRR2A | STAC3 | SMG9 |  |
| SPP1 | PLPPR4 | FLAD1 |  |
| SKIL | DOCK8 | CTPS1 |  |
| SMURF2 | WDFY4 | AURKB |  |
| SFRP2 | RGS1 | PSMD12 |  |
| CXCL5 | SIGLEC11 | BYSL |  |
| CCL5 | CD1B | DNA2 |  |
| BDNF | UBD | TOPBP1 |  |
| BCL2 | PDE4B | ATP13A3 |  |
| RB1 | PBX4 | MEST |  |
| PTPN11 | PRLR | BRIX1 |  |
| MIR491 | ACSM5 | ORC6 |  |
| MIR485 | SULT2B1 | KNSTRN |  |
| CEMIP | NOVA2 | FANCA |  |
| SALL4 | IGLL1 | CKAP2 |  |
| TMPRSS4 | REST | FTSJ1 |  |
| MAPK8 | UTS2 | IPO9 |  |
| PRKCA | JAM2 | WDR46 |  |
| MEG3 | NECTIN3 | ARPC5 |  |
| TUG1 | GIMAP7 | NME1 |  |
| SEMA4C | GNGT2 | ALG8 |  |
| PIN1 | LAG3 | PALB2 |  |
| PGF | FERMT3 | KIF15 |  |
| SERPINF1 | ASRGL1 | FUBP1 |  |
| GOLM1 | EDAR | BMS1 |  |
| PAWR | PSAP | CGAS |  |
| MIR375 | TMEM150B | CENPI |  |
| MIR361 | PREX1 | HSP90AB1 |  |
| NODAL | SALL2 | CSE1L |  |
| ATM | STAB1 | GORAB |  |
| MUC4 | ICAM2 | ZBTB33 |  |
| MMP7 | HSP90AB1 | CKAP5 |  |
| FOXO4 | LYPD3 | PLOD3 |  |
| MAP3K4 | TGFBR1 | ENOPH1 |  |
| MAP3K3 | MS4A7 | LRRC8B |  |
| MDM2 | CLEC14A | BIRC5 |  |
| MIR96 | ADGRL4 | CMTM6 |  |
| MIR31 | SIGLEC17P | EBNA1BP2 |  |
| MIR222 | HTRA4 | CCNA2 |  |
| MIR218-1 | CLOCK | HASPIN |  |
| MIR181A1 | MMP2 | CST1 |  |
| MIR194-1 | P2RY13 | PSMD11 |  |
| MIR185 | EP300 | PROSER1 |  |
| MIR182 | PRKAR2B | CENPE |  |
| MIR181A2 | SERPING1 | DHX34 |  |
| MIR17 | POU2F2 | SLC20A1 |  |
| MIR143 | SPATA13 | DTX3L |  |
| MIR130A | LILRB5 | GEN1 |  |
| MIR128-1 | GIMAP8 | COA7 |  |
| MIR10A | FBN1 | MCM3 |  |
| JUNB | CCL7 | BRCA2 |  |
| ITGA5 | IL22RA2 | CCT5 |  |
| IGFBP7 | VEGFA | NKRF |  |
| HSPB1 | P2RY8 | PCNA |  |
| HSPA4 | TNNT2 | TIMM8A |  |
| HOXB7 | ICOS | CCDC43 |  |
| FOXA2 | HLA-B | TDP1 |  |
| HMOX1 | IFIH1 | TTI1 |  |
| HDGF | GATM | TTK |  |
| HAS2 | LAP3 | HEATR1 |  |
| SENP1 | SFRP2 | DNAJC2 |  |
| RGCC | PPM1H | PPIL1 |  |
| NEAT1 | RASGRF2 | SGO2 |  |
| GPC3 | GPR65 | ATP2A2 |  |
| USP22 | RNASE2 | TBC1D31 |  |
| FOXO1 | PFDN2 | EME1 |  |
| DKK1 | IFNK | UTP15 |  |
| EFEMP1 | VMO1 | ODF2 |  |
| VWCE | MINDY2 | FANCF |  |
| F2R | IFNW1 | C1orf131 |  |
| ELF5 | GZMH | CENPN |  |
| EPHA2 | FCGR1B | SNX10 |  |
| EDN1 | RGL4 | NCAPG2 |  |
| JAG1 | PLPP7 | LACTB |  |
| DLX2 | CRYBG2 | NCL |  |
| CTSL | HIST1H2AG | SF3B3 |  |
| PARP1 | LAMA4 | ADAT1 |  |
| CRKL | CD3D | CDC20 |  |
| FERMT2 | RFTN1 | DPP3 |  |
| TXNIP | CDH5 | PATL1 |  |
| SPRY2 | CREBRF | FIGNL1 |  |
| ADAM10 | DCBLD1 | YDJC |  |
| TRIM28 | TIMP2 | UGGT1 |  |
| NAMPT | FCGR3A | SKA1 |  |
| MIR1271 | IL17B | ADAR |  |
| HDAC6 | C1QTNF7 | BMP1 |  |
| ZBTB33 | HCP5 | SAPCD2 |  |
| CDC42 | TMEM229B | COL1A1 |  |
| HS3ST3B1 | AKAP5 | JPT2 |  |
| SETDB1 | HEPHL1 | GGCT |  |
| HDAC4 | PSMC3 | MSH2 |  |
| UBE3C | ITGAM | SUV39H2 |  |
| BAG3 | PALM2-AKAP2 | LMNB2 |  |
| MAGED1 | NMI | PSME3 |  |
| CD36 | IGF1 | DNAAF5 |  |
| ABCG2 | OASL | DDIAS |  |
| NTN1 | S100A7 | WDR62 |  |
| ADIPOQ | HCST | YTHDF1 |  |
| RNF8 | LCP2 | OTX1 |  |
| ACVR1 | LDLRAD4 | TYMS |  |
| CCNA2 | NBEAL1 | NUP37 |  |
| SQSTM1 | KCNMB1 | C1QTNF6 |  |
| SPHK1 | APOC1 | TRMT6 |  |
| HDAC3 | TM6SF1 | PTPN2 |  |
| WISP3 | PRKCQ | TIPIN |  |
| NRP2 | KCTD12 | ALG6 |  |
| RUNX3 | VPREB3 | MRTO4 |  |
| STC2 | CYBA | HMGXB3 |  |
| RUNX1 | IL20RB | DPY19L1 |  |
| TNFSF11 | HERPUD1 | NOL6 |  |
| BHLHE40 | CD180 | TCOF1 |  |
| LGR5 | TSHZ3 | DDX39A |  |
| HAVCR2 | HLA-DRB1 | TMEM33 |  |
| AFAP1L2 | FAM78A | VAV2 |  |
| DYRK2 | TPK1 | BGN |  |
| PITPNM3 | SYTL3 | SLX4IP |  |
| AXIN2 | KRT14 | SNRPA1 |  |
| AXIN1 | ERAP1 | POLR1C |  |
| NCOA3 | FRZB | CNOT11 |  |
| ACTN4 | PLXND1 | UTP14A |  |
| ESRP2 | ADAMTS9 | NAT9 |  |
| PEAK1 | TESC | GART |  |
| CA9 | DCHS1 | GTF2E1 |  |
| YWHAZ | HSPA2 | SRSF2 |  |
| KDM6A | IFNA2 | ZNF195 |  |
| TUFT1 | UBASH3A | C8orf76 |  |
| TPBG | PRL | HNRNPF |  |
| TP53BP2 | IL21R | POP1 |  |
| TLE1 | TRPC4AP | ZDHHC9 |  |
| TIMP1 | IL4R | CASP8 |  |
| TIAM1 | HIST1H2AE | TEDC2 |  |
| TGFB1I1 | COL18A1 | NIP7 |  |
| TEAD1 | KCNAB2 | SET |  |
| TCF21 | PDE3A | BRIP1 |  |
| MIR616 | MGAT5 | ORC1 |  |
| MIR612 | RAB20 | NCBP2 |  |
| MAP3K7 | CX3CL1 | NOLC1 |  |
| STAT5B | ITGAD | NUFIP2 |  |
| STAT5A | TBXAS1 | APOC1 |  |
| STAT1 | SIGLEC7 | RAD54L |  |
| BRAF | GAB3 | NSUN2 |  |
| SRI | PCED1B-AS1 | PBK |  |
| SPOCK1 | LILRA6 | UTP20 |  |
| SOX5 | COL14A1 | SLC25A32 |  |
| BMP6 | CD302 | GMPS |  |
| SKP2 | KCNJ8 | UBAP2 |  |
| SIM2 | HLA-DMA | BOP1 |  |
| SDC2 | CLEC12A | CDKN3 |  |
| CCL19 | COL6A3 | UBFD1 |  |
| CEACAM1 | CXorf21 | SF3B4 |  |
| RPS6KB1 | SULT1C2 | MCM6 |  |
| ALX4 | KLHDC10 | DHX9 |  |
| RNH1 | LRP6 | MTBP |  |
| CCND1 | PTPN22 | CSTF1 |  |
| KDM5A | ACVR1B | CDK5RAP1 |  |
| ACTA2 | IL4 | BLOC1S3 |  |
| RAF1 | CXCR2 | ISG20L2 |  |
| PTX3 | DBH | CCT3 |  |
| CIP2A | ADA2 | NONO |  |
| MTA3 | MRVI1 | DENR |  |
| MIR506 | TRIM61 | PLA2G7 |  |
| MIR497 | CD40LG | RBL1 |  |
| MIR494 | PLD4 | SEPTIN11 |  |
| MIR489 | PATL2 | TBRG4 |  |
| PTHLH | CTSG | TGS1 |  |
| PBXIP1 | GGT1 | DEPDC1 |  |
| PAK5 | RELN | JRKL |  |
| CTNNBIP1 | FCGR2A | CHEK2 |  |
| KLK6 | FCGR2C | WDR4 |  |
| PRSS8 | UBA7 | CPSF6 |  |
| PROP1 | ACTA2 | ZNF146 |  |
| MAP2K1 | LPL | EIF3B |  |
| ERBIN | IL22 | MFSD12 |  |
| BTBD7 | SYT11 | CENPU |  |
| PRKAA1 | DOCK4 | ATP13A1 |  |
| TRIM62 | P4HA3 | FAF2 |  |
| RNF111 | SDC2 | SDS |  |
| IL17RD | TNFRSF1B | HROB |  |
| PLAUR | ACSL5 | TFB2M |  |
| PKD1 | TSHR | KNL1 |  |
| CDK14 | HMGB1 | AGRN |  |
| PFN2 | GJB2 | LARP1 |  |
| SUFU | SCARF1 | NUP107 |  |
| IL23A | CCL19 | P3H1 |  |
| MAGEC2 | EIF2AK1 | SMARCD1 |  |
| HOOK1 | ZNF831 | PAFAH1B3 |  |
| PDCD1 | LCOR | PTBP3 |  |
| CRIM1 | BICC1 | UBXN4 |  |
| EGFL7 | KLRK1 | PAQR4 |  |
| PCBP1 | CTLA4 | WDFY1 |  |
| F11R | AQP1 | CHAF1A |  |
| SERPINE1 | FCER2 | CLDN1 |  |
| FURIN | FCRL3 | DDX10 |  |
| PEBP1 | DYSF | CKS1B |  |
| P2RY2 | ADGRF4 | VMA21 |  |
| NFKBIA | STXBP6 | RIF1 |  |
| NF1 | LINC00926 | MGME1 |  |
| NCL | SPAG4 | TTF2 |  |
| CEACAM6 | GIMAP5 | TTLL4 |  |
| MIR196B | MMP14 | PARP14 |  |
| MIR373 | TIMD4 | KBTBD2 |  |
| MIR135B | CCL21 | TMEM39A |  |
| ZFAS1 | RUFY4 | TREM2 |  |
| MMP3 | TMEM140 | RNF149 |  |
| MMP1 | DPP8 | STK3 |  |
| ASCL1 | SDSL | ATXN2L |  |
| MGAT3 | EVI2B | BORA |  |
| MDK | SCUBE3 | SRRT |  |
| MCL1 | GSDMA | ORC2 |  |
| MIR34C | SAMHD1 | KIF22 |  |
| MIR33A | ADAM12 | MFSD14B |  |
| MIR30D | KIR2DL4 | XPO6 |  |
| MIR301A | AQP9 | FUCA2 |  |
| MIR27A | CEP128 | HSP90B1 |  |
| MIR24-1 | ARID5A | SNRPD1 |  |
| MIR224 | ZNF80 | FAM136A |  |
| MIR223 | IL17RA | XPO5 |  |
| MIR218-2 | CD163 | ZNF485 |  |
| MIR206 | NFKB2 | XPOT |  |
| MIR20A | CARMIL2 | ILF3 |  |
| MIR199A1 | RASSF2 | HNRNPU |  |
| MIR193A | PSMD11 | POLR1A |  |
| MIR191 | RENBP | SMPD4 |  |
| MIR187 | INHBC | ERCC6L |  |
| MIR183 | SLC34A2 | GINS4 |  |
| MIR16-1 | TTBK2 | NCAPD2 |  |
| MIR15B | FCMR | CDC25C |  |
| MIR15A | WIPF1 | DARS2 |  |
| MIR153-1 | NXPE4 | DUSP12 |  |
| MIR146A | LILRB2 | PDIA3 |  |
| MIR135A1 | CD48 | SASS6 |  |
| MIR134 | GZMA | ZMIZ2 |  |
| MIR132 | FUCA1 | CDK2 |  |
| MIR124-3 | CXCL8 | ANKLE2 |  |
| MIR124-2 | SLC25A45 | RPN2 |  |
| MIR122 | ZEB2 | NASP |  |
| MIR101-2 | STAP1 | RMI2 |  |
| MIR100 | DMKN | MTERF3 |  |
| LMNA | PLPP3 | NPRL3 |  |
| LGALS3 | FBLN2 | XRN2 |  |
| LASP1 | MED13L | RSRC1 |  |
| LAMA5 | TNIK | PLBD2 |  |
| KIT | CD28 | PRPF4 |  |
| CD82 | MAN1A1 | STT3A |  |
| JARID2 | SP100 | NCAPD3 |  |
| ITGB4 | TNFRSF12A | RRP9 |  |
| ITGA3 | PSMD10 | HSPA5 |  |
| ITGA2 | PSMB10 | TOP1 |  |
| AQP5 | OTOA | IQANK1 |  |
| AQP3 | SDR9C7 | TCERG1 |  |
| IL11 | BMP2K | TAF1A |  |
| CXCR2 | CLIP3 | CHTF18 |  |
| MACC1 | NHSL2 | MYO1B |  |
| TNC | CD200R1 | TARBP1 |  |
| HES1 | GMPR | PPRC1 |  |
| PRMT1 | SIRPG | PIGU |  |
| HRAS | SOX5 | DDX11 |  |
| HOXB9 | TGFBRAP1 | NAA15 |  |
| HOXA10 | PSMD5 | PAK1IP1 |  |
| HMGB3 | ARHGAP18 | ANP32E |  |
| HIC1 | PTPRC | SMC2 |  |
| HDAC2 | PTGIS | MIS18BP1 |  |
| H2AFX | FAM171B | CDC123 |  |
| GSN | ACSS3 | TFAP4 |  |
| UHRF1 | ADAMTS10 | ARFGAP1 |  |
| CPS1-IT1 | CD300LF | RHBDF2 |  |
| HIPK2 | CCSER1 | CCDC86 |  |
| GRN | CD7 | GATC |  |
| GPR32 | C5orf56 | PLA2G15 |  |
| ANG | P2RX7 | STX6 |  |
| GLI2 | PLEK | NUP160 |  |
| GJB1 | RCAN2 | PTGES3 |  |
| NOX1 | FCGR1CP | HGH1 |  |
| LRIG1 | MMP25 | SSRP1 |  |
| SLC39A6 | GLRX | TBL2 |  |
| FZD2 | HAVCR2 | NUP43 |  |
| FUT4 | P2RY10 | BZW2 |  |
| BRD4 | TIFAB | ATP2C1 |  |
| SIRT3 | TNFSF8 | IPO4 |  |
| DICER1 | ETV7 | ADAM10 |  |
| SATB2 | TRIR | POLQ |  |
| BOP1 | HLA-DQB1 | STMN1 |  |
| EPB41L3 | GPR18 | F2R |  |
| KDM4B | CPVL | NELFCD |  |
| FGF9 | EDNRB | TYW1 |  |
| FGF1 | CCNT1 | UMPS |  |
| ETV4 | LHFPL6 | DESI2 |  |
| ESRRA | UBE2L6 | ATR |  |
| ESR2 | PARP9 | SLC35A2 |  |
| ERG | PDE1A | MORC2 |  |
| EPS8 | S1PR1 | SH3KBP1 |  |
| EPO | XKR8 | CDC6 |  |
| EPHB2 | SSC5D | TRIAP1 |  |
| GKN2 | GPC5 | MTDH |  |
| TET3 | SBSN | CCAR1 |  |
| AHR | P2RY11 | RAE1 |  |
| E2F1 | KCNH2 | E2F7 |  |
| HBEGF | GIPC3 | SLC37A3 |  |
| DLX4 | AZGP1 | PIK3AP1 |  |
| DAB2IP | PRAM1 | RAN |  |
| PAQR3 | RNASE7 | NR2C2AP |  |
| CTSZ | IFNGR2 | ILDR1 |  |
| GLIPR2 | EDA2R | MRGBP |  |
| SLC30A7 | SAMD9L | EARS2 |  |
| RBFOX3 | CNR2 | MZT1 |  |
| CSNK2B | NFATC2 | PODXL |  |
| CRYAB | SLAMF1 | KRT18 |  |
| LINC00261 | BFSP2 | OIP5 |  |
| CRK | FLT4 | EEF1AKNMT |  |
| CREB1 | CNTF | CDT1 |  |
| CLDN7 | PANX1 | FAM111B |  |
| CLDN4 | MILR1 | HSPE1 |  |
| CLU | IL4I1 | CXCL16 |  |
| UHRF2 | CEACAM4 | MPZL1 |  |
| EHMT2 | SELENBP1 | TMEM267 |  |
| PROKR1 | CD58 | RBM19 |  |
| WASF3 | HSPA1A | ATP6V1C1 |  |
| CFTR | SMIM1 | FBXO5 |  |
| KHDRBS1 | PSME2 | YRDC |  |
| TRIM16 | GRAP | POGK |  |
| CEBPB | ADORA2A | TMEM63A |  |
| CEBPA | TMEM200A | CLDN7 |  |
| HOXB13 | PVRIG | PDCD2L |  |
| TACC3 | IFIT2 | ZNF124 |  |
| CDX2 | IFNGR1 | MCM8 |  |
| FAM3C | ACTN1 | GNL3 |  |
| CDKN2A | KMO | VARS1 |  |
| CDK5 | ZMYND15 | MMS22L |  |
| NPTN-IT1 | NCF4 | SEH1L |  |
| SPRY4-IT1 | GLIPR2 | RPP25 |  |
| ZEB2-AS1 | BATF | POU2F1 |  |
| MIR1236 | MBNL3 | USP14 |  |
| MIR675 | TLR3 | LIF |  |
| FGF19 | PRTG | SLC29A2 |  |
| RBM8A | PLEK2 | POLE3 |  |
| MRC2 | RTP5 | TONSL |  |
| GAB2 | RAB37 | FANCL |  |
| CD151 | NLRP12 | IL17RB |  |
| HDAC9 | PABPC5 | TDG |  |
| SEMA3E | FN1 | RTKN |  |
| VGLL4 | GBP4 | PLXNA1 |  |
| CD63 | CRISPLD2 | SLC6A6 |  |
| MICAL2 | CCDC170 | TTC27 |  |
| ISG15 | MS4A2 | ARL6IP6 |  |
| PITPNM1 | RHOJ | GRIN2D |  |
| CXCL14 | SBNO1 | LIN9 |  |
| ONECUT2 | CTSW | LRRC59 |  |
| ROCK2 | WNT2 | U2SURP |  |
| RASAL2 | GBP1 | PRKDC |  |
| EIF2AK3 | ARHGAP45 | BRCA1 |  |
| MAP4K4 | CHAC1 | PPM1H |  |
| QKI | BHLHA15 | MLEC |  |
| HAND1 | HMSD | PAWR |  |
| CYP7B1 | ARHGAP25 | HPRT1 |  |
| MUC16 | EGFR | POLG2 |  |
| FOXP2 | TXK | SUZ12 |  |
| ZFYVE9 | APOC2 | STK4 |  |
| SLC9A3R1 | IL23A | RNASEH2A |  |
| KL | C15orf48 | AUNIP |  |
| LHX2 | PPP1R16B | THADA |  |
| IGSF8 | ICK | TCF3 |  |
| NREP | PIK3CG | DIAPH3 |  |
| CD14 | SPRR2D | EXOSC2 |  |
| NOG | ITGBL1 | FAM83H |  |
| LRRFIP1 | GPR25 | TTC13 |  |
| MARVELD3 | HIC1 | CCT2 |  |
| ARHGEF2 | CLIC2 | DTYMK |  |
| SMC3 | MNDA | INTS8 |  |
| LATS1 | FCRLA | MTPAP |  |
| NMI | CD68 | IVNS1ABP |  |
| AOC4P | NKG7 | SMC4 |  |
| PSTPIP1 | HSPA6 | CEMIP2 |  |
| SOCS3 | GPR84 | CCNJ |  |
| HS6ST2 | CCR4 | NVL |  |
| CCNG2 | JMY | NLN |  |
| CDKL2 | STRIP2 | COX19 |  |
| BTRC | UBR1 | SLC8B1 |  |
| CCND2 | ARHGEF15 | SSB |  |
| TIMELESS | MSR1 | DNAJC10 |  |
| NR1I2 | LAIR2 | DHX15 |  |
| KAT2B | DOK3 | MYO19 |  |
| INPP4B | HLA-DPA1 | POLR1G |  |
| SCEL | FCGR1A | OBI1 |  |
| TNFRSF11A | PADI2 | R3HDM1 |  |
| TNFSF10 | ST3GAL5 | TRMU |  |
| TNFSF12 | KLHL6 | SETDB1 |  |
| CBR1 | CALR | PES1 |  |
| EED | CLEC4M | CANX |  |
| ABCC3 | KLRC3 | ABHD11 |  |
| CBLB | NAALADL1 | STIP1 |  |
| EIF3I | ARL6IP5 | SMG7 |  |
| IRS2 | F13A1 | ARL6IP1 |  |
| SCIN | FCGBP | EDEM2 |  |
| IKBKG | SCARF2 | ACTR5 |  |
| PIK3R3 | KIT | SLC30A6 |  |
| AJUBA | ERN1 | DHX33 |  |
| PPM1D | TNFRSF11B | PTDSS1 |  |
| GEMIN2 | AGAP2 | C2orf15 |  |
| USMG5 | COL15A1 | CASK |  |
| SEMA7A | RECK | ETV4 |  |
| LOXL3 | AVPR1A | SNX8 |  |
| HINT2 | SHE | PARP1 |  |
| SPZ1 | SFMBT2 | NSD2 |  |
| TEAD2 | MYEF2 | CNPY2 |  |
| FOXN1 | TPSG1 | CD276 |  |
| CUL3 | CAMK1 | PHF14 |  |
| KAT8 | MAP4K1 | CAPRIN1 |  |
| SPOP | KIR3DL1 | SNRPG |  |
| MARVELD1 | GFI1 | POLR1F |  |
| ITCH | PTGER2 | CHAF1B |  |
| YPEL3 | CD2 | PAXIP1 |  |
| CAPZA1 | HECW2 | NRAS |  |
| CAPNS1 | ST8SIA4 | IRF3 |  |
| SMC1A | FGR | TMEM62 |  |
| USP9X | MYO7A | NOL8 |  |
| VANGL1 | VWF | RAB3IP |  |
| TRIM11 | VSTM4 | IKBIP |  |
| WNT5B | NLRC4 | MCM7 |  |
| IFT88 | PSMD6 | RANGAP1 |  |
| SETD7 | KRT16 | GABRD |  |
| PTP4A2 | LILRA1 | PFAS |  |
| MED28 | GHRL | RBM28 |  |
| ELL3 | CD247 | RACGAP1 |  |
| NUBPL | ADAM28 | HSPA14 |  |
| FBXO11 | LRRC15 | RPAP3 |  |
| MICALL2 | SYNDIG1 | RELT |  |
| LIN28A | CD80 | HUS1 |  |
| TRPM8 | ARHGEF6 | BRI3BP |  |
| DDR1 | PKP3 | MTIF2 |  |
| ZYX | TRPV3 | DNAJB11 |  |
| ZNF217 | TUBB6 | LYN |  |
| PCGF2 | IFNA21 | VMP1 |  |
| ZNF143 | MEI1 | RNF114 |  |
| MIR802 | LTA | TMTC4 |  |
| MIR454 | LIMS1 | CFAP157 |  |
| MZF1 | MEOX2 | SMC6 |  |
| ZFP36 | CPNE5 | GTF2H3 |  |
| WNT11 | FNIP2 | TGDS |  |
| WNT6 | MS4A4A | MARS2 |  |
| VTN | ETV3 | C5orf34 |  |
| VSNL1 | HMCN1 | PAM16 |  |
| VHL | TRAT1 | NFKBIE |  |
| VCP | IFIT3 | FAM111A |  |
| UCP2 | PDCD1 | CXorf38 |  |
| UCHL3 | GPR132 | THOC2 |  |
| TYRO3 | LOXL2 | TASOR2 |  |
| TYMS | PSMD13 | DUS4L |  |
| TXN | CCL3L1 | COL4A1 |  |
| TUFM | IL18RAP | SMS |  |
| C5AR1 | VPS37D | ZNF121 |  |
| PHLDA2 | SERPINA1 | BAZ1B |  |
| TSC2 | XBP1 | PTTG1 |  |
| TSC1 | LGALS9 | ZNF473 |  |
| MIR663A | VIM | B3GNTL1 |  |
| TRPC5 | STAT5A | EPHB4 |  |
| ACTG2 | CD93 | UHMK1 |  |
| TP53BP1 | CDH20 | RFC5 |  |
| TNXB | TAGAP | ARHGAP39 |  |
| TNS1 | PPP1R9A | WDR5B |  |
| TLN1 | PEAK1 | TAF1D |  |
| TLE4 | RIF1 | ZNF217 |  |
| TJP1 | INMT | TRAIP |  |
| TIMP3 | LST1 | TRMT1 |  |
| NR2F2 | FCRL5 | ARPC1B |  |
| TFCP2 | IL3RA | DPAGT1 |  |
| TDGF1 | GNLY | MND1 |  |
| MIR92B | TGM2 | CACYBP |  |
| MIR646 | LCN10 | PGAM5 |  |
| MIR639 | HLA-G | SLC12A7 |  |
| MIR638 | IL18 | ENTPD6 |  |
| TCF7 | IL7 | PSMD1 |  |
| MIR573 | PTPRJ | EPRS1 |  |
| TBX3 | SIGLEC12 | WDR12 |  |
| TBX2 | TTC21B | FUS |  |
| TAZ | CPXM1 | SPC24 |  |
| KLF5 | TLR7 | RRP1B |  |
| ST14 | P2RX1 | ADAMTS14 |  |
| SREBF1 | PNLIPRP3 | STARD7 |  |
| SOX3 | TSPAN11 | MTF2 |  |
| SON | LAD1 | NOC3L |  |
| SOD2 | FMNL3 | ETV6 |  |
| MIR487B | IL1B | NUP188 |  |
| STK33 | TNFAIP6 | HDGF |  |
| SLIT3 | CXCR3 | LSR |  |
| SLC2A1 | CLEC9A | MSTO1 |  |
| SKP1 | TNFRSF10A | NAA40 |  |
| SKI | ZFPM2 | MMP11 |  |
| ST8SIA1 | DPEP1 | TMEM44 |  |
| ST6GAL1 | PTPN6 | GCA |  |
| SIAH2 | PTH1R | TMEM201 |  |
| SIAH1 | RASSF5 | TRPM2 |  |
| SHC1 | CSF3 | CENPH |  |
| CPEB1 | JCAD | FASTKD3 |  |
| AKTIP | GZMM | GXYLT1 |  |
| SFTPC | FKBP11 | PSME4 |  |
| SOX17 | TMEM106A | RANBP1 |  |
| SEMA4A | CTSS | ARHGAP8 |  |
| ELSPBP1 | ISG20 | SNX5 |  |
| GOLPH3 | OLFML1 | TIGD1 |  |
| CX3CL1 | FAM49A | TOP1MT |  |
| CCL25 | PIK3CD-AS1 | PSMA1 |  |
| CCL22 | LYN | POC1A |  |
| CCL3 | PLCB4 | PASK |  |
| SERPINB4 | GJD3 | CCT8 |  |
| ATXN1 | GPR82 | CDCA7 |  |
| SALL1 | FAS | CHORDC1 |  |
| S100P | NT5E | SUPT16H |  |
| S100A9 | TESPA1 | ADAMTS12 |  |
| S100A8 | CELF2 | TARS2 |  |
| S100A6 | COL6A1 | CHD1L |  |
| S100A2 | CDH3 | CLK2 |  |
| ROS1 | CD160 | NHLRC3 |  |
| RORC | TGFB1 | FKBP9 |  |
| EXOC4 | RHOD | SLBP |  |
| BCL6 | C16orf54 | GMFB |  |
| RGS3 | CCR6 | MAP4K4 |  |
| RDX | TAOK1 | F11R |  |
| HAS2-AS1 | COL11A1 | WDR3 |  |
| RBP2 | CREB3L1 | HS2ST1 |  |
| SENP2 | KLRG1 | NR2C1 |  |
| NTN4 | FSTL1 | CSPP1 |  |
| RASA1 | OSMR | PRIM1 |  |
| PRUNE1 | SPRR1B | ZMYND19 |  |
| CXCL16 | FAM168A | LYAR |  |
| PTPRZ1 | ARRB1 | FOXN2 |  |
| NKX3-2 | COL5A2 | TGIF2 |  |
| PTPN14 | ADRM1 | CIT |  |
| PTPN6 | PPM1M | BRMS1 |  |
| SCUBE2 | CIITA | ZDHHC23 |  |
| EPB41L5 | EIF2AK3 | RPL7L1 |  |
| PTN | COL3A1 | NMI |  |
| STIM2 | BDKRB2 | NUP35 |  |
| MTUS1 | MITF | TICRR |  |
| MKL2 | NME8 | CEP135 |  |
| MIR522 | PSMD14 | TFDP1 |  |
| MIR517C | PSMC4 | NFXL1 |  |
| MIR520G | TNFSF14 | SPAST |  |
| MIR452 | ADAMTS12 | FCHO1 |  |
| RAB22A | TMC8 | PNN |  |
| CAMK1D | ISG15 | TMEM60 |  |
| TBX20 | THEMIS2 | ADAMTS2 |  |
| POGLUT1 | FAM25A | JADE3 |  |
| PROX1 | KCNA3 | TNFRSF10A |  |
| AJAP1 | IL13RA1 | TAF2 |  |
| PRKCQ | VAV1 | DENND4B |  |
| HDAC8 | SAMD3 | TSEN54 |  |
| PAG1 | VEGFB | LRRC58 |  |
| KDM3A | TAP2 | ZC3H3 |  |
| FOXJ2 | QPRT | CLDN4 |  |
| PRKCE | ANO6 | ABCF2 |  |
| PRKAA2 | ADRA2A | COL5A2 |  |
| FERMT1 | CYBB | ABCF1 |  |
| MIR449A | KRT78 | FBXO45 |  |
| LGR4 | TAPBPL | DDX27 |  |
| BRF2 | PRELP | ZFP64 |  |
| IMP3 | CD1E | FOXS1 |  |
| PTPA | CD34 | TAF4 |  |
| PPP1R8 | ACVR2A | NOP14 |  |
| GIPC2 | KCNN4 | RRN3 |  |
| POMC | MAP3K2 | PHF6 |  |
| ERRFI1 | RAPGEF2 | VCPIP1 |  |
| MBD3 | HAPLN3 | CDC25B |  |
| PLS3 | LAT2 | TMC6 |  |
| PLAGL2 | EFTUD2 | CARMIL1 |  |
| PLA2G4A | CSF2RA | RAD21 |  |
| PIK3R1 | MRPS21 | ZNF761 |  |
| SERPINI1 | KRT6A | RAD54B |  |
| ABCB1 | CD209 | RPRD1B |  |
| STK26 | SPRR1A | C4orf46 |  |
| WWOX | CD8A | RMI1 |  |
| UIMC1 | SPARC | TMEM87B |  |
| CYB5R1 | ENPP2 | YEATS2 |  |
| PDGFRB | RAB33A | POLR3K |  |
| ACKR4 | CSGALNACT2 | CHDH |  |
| PDGFB | TMEM79 | CENPM |  |
| LIMA1 | ACOXL | NUP155 |  |
| PRRX2 | ZNF185 | SLC12A9 |  |
| PDE4A | OAS3 | PABIR2 |  |
| UBR5 | PIK3AP1 | PPP4R2 |  |
| PLAC8 | DENND2A | FNDC3B |  |
| TFDP3 | STAT4 | CENPW |  |
| PCSK1 | IFNL2 | NAA10 |  |
| ING4 | OAS2 | UTP4 |  |
| ANGPTL4 | SIGLEC9 | PLAU |  |
| PCMT1 | JSRP1 | SNRPE |  |
| GMNN | CAVIN1 | PRR7 |  |
| PCDH9 | IDO2 | RAD18 |  |
| PBX3 | ZKSCAN1 | POLR2H |  |
| PARD6A | PLA2G2D | DPM2 |  |
| NEUROG3 | FGF7 | PTCD3 |  |
| IL22 | F5 | MFHAS1 |  |
| PRDX1 | OSCAR | CCDC137 |  |
| DUXAP9 | CREB1 | MRPL47 |  |
| OPRM1 | PARM1 | RBM12B |  |
| MIR424 | IL36G | SH2D2A |  |
| MIR382 | PDGFRA | FAM72B |  |
| MIR381 | ABCA6 | CHD7 |  |
| NUCB2 | COL4A1 | PYCR1 |  |
| ROR1 | ACVRL1 | CCT4 |  |
| NTRK3 | GCSAM | HSPH1 |  |
| NPPA | ST6GAL1 | TMEM167A |  |
| NOV | TUBA4A | NARS2 |  |
| NOTCH4 | SIGLEC1 | GLA |  |
| NOTCH3 | ABCB1 | MEN1 |  |
| NME1 | SCIMP | CDC25A |  |
| NKX6-1 | ANKRD36BP1 | SRM |  |
| NFIL3 | IFI27 | FPGS |  |
| NFIC | CALHM5 | REXO4 |  |
| MYL2 | ANXA6 | NLK |  |
| MYD88 | MYO9A | BROX |  |
| MYCN | EMILIN1 | SUV39H1 |  |
| MYBL2 | CD36 | SNRPB |  |
| MYB | MSRB3 | WDR36 |  |
| TRIM37 | SELPLG | SFT2D2 |  |
| MUC2 | MCEMP1 | MSI2 |  |
| MST1R | NCCRP1 | C12orf75 |  |
| MSN | CACNA2D2 | ERI1 |  |
| MIR345 | CSF1R | PTPN12 |  |
| MIR331 | ICOSLG | LRP8 |  |
| MIR326 | LILRA4 | PTK2 |  |
| MMP19 | PRG2 | SRSF6 |  |
| MMP13 | TMIGD2 | FMNL2 |  |
| MMP11 | KDR | PLEKHA8 |  |
| MMP8 | FGL2 | FKTN |  |
| MLLT3 | SRGN | WDR5 |  |
| ASCL2 | PLPP4 | EIF2S2 |  |
| MITF | INHBB | HPS3 |  |
| CXCL9 | CXCR6 | RHPN1 |  |
| MEF2D | CCL16 | LTV1 |  |
| MDM4 | VTCN1 | NUP58 |  |
| ART1 | PPFIA2 | ZBED4 |  |
| SMAD9 | IL22RA1 | NT5C3A |  |
| ARRB1 | THSD7A | NLE1 |  |
| MIR7-1 | RARRES2 | ABL2 |  |
| MIR30E | LPAR4 | SERINC5 |  |
| MIR30B | PDGFRB | MTHFD1 |  |
| MIR302A | APOBR | SRPRB |  |
| MIR29A | PNMA2 | POLE |  |
| MIR26A2 | PLAC9 | MNX1 |  |
| MIR25 | ICAM3 | OTULIN |  |
| MIR24-2 | IL11 | KIF24 |  |
| MIR23B | IDO1 | AGPS |  |
| MIR217 | COL6A6 | FOXD2 |  |
| MIR216A | PSMD2 | LLGL2 |  |
| MIR211 | ITGAX | ORC5 |  |
| MIR208A | APOBEC3D | YWHAB |  |
| MIR199A2 | RAET1G | TRAM1 |  |
| MIR190A | CCL20 | SLC39A1 |  |
| MIR181B1 | PDCD1LG2 | ZSWIM4 |  |
| MIR153-2 | LAX1 | RBM15 |  |
| MIR152 | CTTNBP2 | SFMBT1 |  |
| MIR149 | ZBP1 | STAT1 |  |
| MIR144 | ZEB1 | PRMT3 |  |
| MIR136 | KLHL23 | CCDC138 |  |
| MIR129-1 | C1orf162 | SGPL1 |  |
| MIR125B1 | IL10 | PPA1 |  |
| MIRLET7G | RAB8B | RRP15 |  |
| MIRLET7D | SPIB | CIAO2A |  |
| MIRLET7B | CFP | SMC1A |  |
| LYN | INPP5D | MTG1 |  |
| LTBP1 | KIAA1549 | ADNP |  |
| LRP6 | SYNE1 | TTC26 |  |
| LMNB1 | KLK9 | PUS7L |  |
| LIMS1 | TRIM21 | SMARCC1 |  |
| LIF | GMFG | CCSAP |  |
| LGALS9 | MIR155HG | ATP13A2 |  |
| LAMC2 | LUM | FANCB |  |
| USP17L9P | CHRD | MISP |  |
| RHOG | SNTB1 | SPATA13 |  |
| NANOGP8 | C1orf127 | TFAM |  |
| KRT19 | POMK | EXOC6 |  |
| RHOB | LATS1 | MAK16 |  |
| KMT5A | F2R | NUP205 |  |
| KRT18 | HSD11B1 | POLD1 |  |
| KRT17 | CCL23 | SKP2 |  |
| KRT8 | SLC29A3 | TRUB1 |  |
| KRT7 | SERINC5 | FANCC |  |
| KIF5B | BCL2L14 | ZNF587B |  |
| KDR | CYSLTR1 | LOXL2 |  |
| KCNN4 | CXorf65 | NOC4L |  |
| KCNH1 | ADAMTS14 | MAGOHB |  |
| JAK3 | COL5A3 | AP2B1 |  |
| JAG2 | ADGRA2 | TESMIN |  |
| ITGB6 | LIG3 | ANKEF1 |  |
| ITGB3 | UCP2 | IKBKE |  |
| IRS1 | EMCN | IGF2BP2 |  |
| IRF6 | BGN | MET |  |
| AQP9 | FOXP3 | TIMM17A |  |
| ITGA6 | CMPK2 | CDH24 |  |
| FOXK2 | ADAMTS16 | SUSD1 |  |
| IL18 | ABCA9 | GPAT4 |  |
| IL6R | SLC17A9 | ARHGEF1 |  |
| IL4 | MYO1F | IRAK1 |  |
| FAS | MMP3 | LRPPRC |  |
| IGFBP3 | KRT6C | DDX52 |  |
| MCRIP1 | IGDCC4 | LSM5 |  |
| IRGM | GMIP | MCCC2 |  |
| NANOS3 | EPCAM | NEIL3 |  |
| IDH2 | CACNA2D4 | FAM120A |  |
| IDH1 | HDC | TIMP1 |  |
| ID2 | CD72 | UBA2 |  |
| IRF8 | NCR3 | ODR4 |  |
| RAB43 | CEACAM21 | POLA1 |  |
| HTN1 | PECAM1 | TNS3 |  |
| HSP90AA1 | ISLR | NTAQ1 |  |
| HSPB2 | CRLF2 | CEP85 |  |
| BIRC2 | LRMP | TM9SF4 |  |
| HRG | CCDC141 | BARD1 |  |
| HPGD | TRAF1 | LCLAT1 |  |
| HOXD9 | CCL1 | RABGGTB |  |
| APBB1 | PLCL1 | COL10A1 |  |
| HOXA9 | IL2RB | HSPA9 |  |
| HNRNPAB | MED13 | GNL3L |  |
| NR4A1 | ZNF671 | NUP50 |  |
| HK2 | FAP | SMG5 |  |
| HIP1 | C4A | AATF |  |
| EHD2 | MS4A14 | CCDC34 |  |
| ANXA5 | HSPA1B | IARS2 |  |
| H2AFZ | GUCY1A2 | CDK16 |  |
| ANXA1 | HCK | C1GALT1C1 |  |
| REPIN1 | CTSL | NEDD1 |  |
| OLA1 | PAG1 | BCAP31 |  |
| CARD10 | PLVAP | RIOK1 |  |
| GSK3A | ABCA8 | TMEM68 |  |
| BRD7 | C3AR1 | THOC6 |  |
| TBK1 | HGF | RNFT2 |  |
| FHOD1 | PGM5 | MAP3K21 |  |
| GRIN1 | ATP8B4 | NEK8 |  |
| ANPEP | ROBO4 | GAR1 |  |
| TUSC7 | HFE | DBF4B |  |
| LAMA1 | SH2D5 | USP1 |  |
| CAVIN1 | SMPDL3B | KDELR2 |  |
| GPI | SLC24A4 | TBL1XR1 |  |
| GOLGA2 | ARSB | GATAD2A |  |
| GLRX | COLGALT2 | TMEM185B |  |
| GLS | ITGB7 | PABPC1L |  |
| EML4 | PSMC6 | BBC3 |  |
| GLO1 | PDE1B | FGFR4 |  |
| GLS2 | TMEM47 | YWHAG |  |
| AGO2 | DSG1 | TASP1 |  |
| DKK3 | PRSS27 | PPFIA1 |  |
| BBC3 | BHLHE41 | FAAP24 |  |
| LYPD3 | HPGDS | PYCR3 |  |
| GJB2 | FSTL3 | MED14 |  |
| AMHR2 | LEP | HAUS2 |  |
| GH1 | NECTIN2 | THEM6 |  |
| GATA6 | OVOL1 | GFM1 |  |
| GATA1 | CCRL2 | LEO1 |  |
| GAPDH | KIR2DL3 | CCDC14 |  |
| SIN3A | SIGLEC8 | TMC7 |  |
| LETMD1 | IFI35 | PFDN2 |  |
| BRMS1 | TMEM119 | ADAP1 |  |
| ARMC8 | PREX2 | LRR1 |  |
| TXN2 | RSAD2 | RPF2 |  |
| SPDEF | CMA1 | AP3M2 |  |
| FSCN2 | COL12A1 | TBC1D30 |  |
| CBY1 | HLA-DQB2 | EHMT2 |  |
| CERS6 | PCDH17 | CRNKL1 |  |
| NR5A2 | DLC1 | GNPNAT1 |  |
| FSHR | DOK1 | MFSD13A |  |
| IL27 | SLC10A2 | IARS1 |  |
| ZMYND8 | SPN | NOM1 |  |
| CCNDBP1 | TNFRSF10B | ACAN |  |
| PADI4 | BTN2A2 | CSTF2 |  |
| MMD | CILP | CEP83 |  |
| SCRIB | LPXN | BAZ1A |  |
| SUZ12 | OLFML3 | CMTM1 |  |
| NCSTN | CD14 | HDAC2 |  |
| ARHGEF12 | BLK | FRMD8 |  |
| NEDD4L | VGLL3 | HEXB |  |
| FMR1 | SERPINE1 | ZNF239 |  |
| EXOC7 | PSMC5 | FAM72A |  |
| FLT1 | ZNF215 | HMGA1 |  |
| FLNA | AKAP12 | KIF26B |  |
| KDM6B | FCER1A | NBN |  |
| PLXND1 | GIT2 | DROSHA |  |
| PEG10 | NLRP3 | TUG1 |  |
| SNW1 | PTPN7 | EFNA4 |  |
| ALDOB | EPS8L1 | IFI30 |  |
| CEP164 | CR1L | ZNF678 |  |
| FKBP5 | DHRS1 | BAG4 |  |
| PHLDA1 | EBF1 | E2F8 |  |
| XRN2 | CD79A | PRPF3 |  |
| FHL2 | ADAMTSL2 | PTCD1 |  |
| FHL1 | CLIC3 | SHTN1 |  |
| SCUBE3 | DAB2 | TMEM123 |  |
| FCN2 | PRRX1 | ANAPC1 |  |
| ZEB1-AS1 | CD3E | CENPQ |  |
| FBP1 | PDGFB | DCLRE1A |  |
| FASN | AIF1 | ATAT1 |  |
| FBLN1 | C9orf139 | SAAL1 |  |
| ALDH1A1 | ENPP4 | NCLN |  |
| F3 | SPNS3 | PIMREG |  |
| F2RL2 | GPR174 | P4HA1 |  |
| F2 | DENND6B | NUFIP1 |  |
| ETV1 | PAK5 | NDUFAF6 |  |
| ETS2 | CHRDL1 | PRICKLE3 |  |
| ETS1 | FAT4 | POLA2 |  |
| ESRRB | THEMIS | LAMC2 |  |
| ALAD | IGFLR1 | AHCY |  |
| ERN1 | AMH | ATAD3B |  |
| ERF | HHLA2 | DTX4 |  |
| EPHB3 | KLRD1 | PDE7A |  |
| EPHA8 | NCF1C | HDAC1 |  |
| EPHA4 | KCND2 | STAC3 |  |
| EP300 | ECM2 | SNAPC4 |  |
| ENO1 | ENPP3 | XRCC3 |  |
| ENG | FGF14 | MCM5 |  |
| EMP3 | PDE6G | AJUBA |  |
| FBXO45 | IL13 | DYNC2I2 |  |
| RPL22L1 | PAFAH1B2 | ZNF765 |  |
| ELK3 | TNFRSF6B | TBC1D24 |  |
| ELK1 | RBP5 | ZNF92 |  |
| CYP4Z1 | RNF180 | TMEM209 |  |
| ELAVL1 | DOK6 | C2CD3 |  |
| EIF5A | MFAP3 | ANGPT2 |  |
| EIF4G1 | TNFSF18 | TIGAR |  |
| EIF4E | TVP23A | CBX4 |  |
| EIF2S1 | MRPL27 | ESCO2 |  |
| RMST | TBC1D10C | RNF2 |  |
| EFNB2 | RFXAP | ALDH18A1 |  |
| EEF1D | SPOCK2 | DPM1 |  |
| EDNRA | FCAR | GOLT1B |  |
| ECT2 | HSPA4 | SYNCRIP |  |
| DVL2 | TCIRG1 | PRAG1 |  |
| AGTR1 | LEPR | PDIA4 |  |
| DNMT3B | SH2D1A | MIS18A |  |
| DDX5 | THBS1 | B3GALT6 |  |
| DAPK1 | GPIHBP1 | HMBS |  |
| CYP3A5 | TLR5 | STRIP2 |  |
| PEBP4 | IL5 | NEK6 |  |
| ZNF746 | GPSM3 | RFC2 |  |
| CYP1B1 | RFXANK | TCP1 |  |
| CMTM8 | FMNL1 | MYBBP1A |  |
| CSPG4 | ARHGAP9 | ESF1 |  |
| VCAN | DENND1C | NFYA |  |
| CSK | OAS1 | MARVELD3 |  |
| CSF2 | GPR55 | ITPA |  |
| CRP | C7 | RBBP8 |  |
| CRMP1 | EBF2 | NEMP1 |  |
| FOXR2 | RNF222 | NUP133 |  |
| CREBBP | NFYC | ITGAX |  |
| ADM | IL10RB | HMGN1 |  |
| KLF6 | TMEM233 | GNPDA1 |  |
| COL8A2 | LHFPL2 | MRM1 |  |
| COL8A1 | RASGRP3 | DNAJA1 |  |
| KLF17 | TIMP3 | RCN1 |  |
| CNTN1 | DPEP2 | G2E3 |  |
| MSI2 | KITLG | CDCA4 |  |
| CCR6 | AMPH | INCENP |  |
| CCR5 | IGSF6 | TPP2 |  |
| CLK2 | SLIRP | AMMECR1 |  |
| LRG1 | ITGB2 | ALG10 |  |
| CKS2 | LAMA2 | CDCA3 |  |
| BATF2 | SNAI3 | CCDC18 |  |
| CTHRC1 | CRTAM | FERMT1 |  |
| CIRBP | LTC4S | ADAM17 |  |
| MGLL | PTGFR | AGO2 |  |
| VSIG4 | CASP5 | PIK3CB |  |
| EGLN3 | UBXN11 | CEP78 |  |
| PKP3 | THY1 | NCBP1 |  |
| RASSF1 | FAM13C | RRP1 |  |
| FSTL1 | CYP1B1 | NANP |  |
| PTP4A3 | SLC8A1 | PGM3 |  |
| BVES | PLIN3 | HOXC9 |  |
| FOXN3 | LCP1 | OLR1 |  |
| PIM2 | MET | PA2G4 |  |
| EHD1 | COLEC12 | KCTD13 |  |
| PPARGC1A | FGD3 | TOMM40 |  |
| PGRMC1 | CD22 | DCP2 |  |
| HPSE | PARVG | CEMIP |  |
| CCR9 | BTK | NUP42 |  |
| MTHFD2 | CCR1 | HNF1A |  |
| KDM5B | DSC1 | PARP9 |  |
| GNA13 | PROM1 | ZNF131 |  |
| LEFTY1 | IL2 | BRAT1 |  |
| PDPN | FAM107A | AEN |  |
| CXCL13 | C1orf54 | CALU |  |
| FBLN5 | MAOB | ADRM1 |  |
| CEACAM5 | DERL3 | THY1 |  |
| FST | BATF2 | UHRF1BP1 |  |
| MAD2L2 | CRCT1 | PPAN |  |
| TAB1 | CTSZ | LARP4 |  |
| RACK1 | GNS | NUP98 |  |
| PAK4 | SVOPL | ESRP1 |  |
| CDKN1A | ZCCHC24 | RCCD1 |  |
| SPRY1 | ENPEP | ZW10 |  |
| MSLN | BEX5 | ZNF138 |  |
| PSME3 | PRF1 | NOP16 |  |
| LINC01186 | GNG2 | SPINT2 |  |
| CDK3 | PIK3R6 | DCAF1 |  |
| G3BP1 | RUNX1T1 | AZIN1 |  |
| TRAP1 | VENTX | EMC1 |  |
| CDH13 | IL1R1 | TET3 |  |
| CDH11 | FCRL4 | LAMP3 |  |
| NR1H3 | FAM155A | ARHGEF38 |  |
| MICA | RAET1E | PVR |  |
| OCLN | NTRK1 | NCEH1 |  |
| DNAJB6 | ESAM | ELK1 |  |
| MIR1181 | IL27 | PUM3 |  |
| CDH5 | C1QA | NOC2L |  |
| FRAT1 | UBXN1 | RPIA |  |
| PDCD6IP | FHL5 | STT3B |  |
| HDAC5 | PLCL2 | SRGAP2 |  |
| MIR124-2HG | LDB2 | UAP1L1 |  |
| MIR875 | SIRPB1 | DCUN1D5 |  |
| MIR888 | CLEC4E | STAT2 |  |
|  | CHRNA6 | RCN2 |  |
|  | ERP27 | EEF1E1 |  |
|  | CXCL11 | DNAJC9 |  |
|  | GREM1 | WDR77 |  |
|  | GZMK | CCDC77 |  |
|  | SEC24D | PDRG1 |  |
|  | NLRC5 | ZNF587 |  |
|  | ALOXE3 | KHDC4 |  |
|  | ANKRD36BP2 | AHCTF1 |  |
|  | RIMKLA | TMEM104 |  |
|  | RTN1 | SHMT2 |  |
|  | STARD13 | CEP72 |  |
|  | TLR1 | POLD3 |  |
|  | BANK1 | SLC44A1 |  |
|  | PSMB8 | CYP27B1 |  |
|  | IFNE | ZNF107 |  |
|  | SLIT2 | CPNE1 |  |
|  | DUSP4 | FCGR3A |  |
|  | S100B | GMNN |  |
|  | IRF4 | LPCAT1 |  |
|  | CLEC4A | SBNO2 |  |
|  | SLAMF7 | MTG2 |  |
|  | RAI2 | PMM2 |  |
|  | PTPRO | AP1S3 |  |
|  | ALOX5AP | PANX1 |  |
|  | CSF1 | CLCN5 |  |
|  | FLT1 | CDK5RAP3 |  |
|  | CDC42SE2 | SMC3 |  |
|  | HLA-A | CKLF |  |
|  | HTRA3 | BMP8A |  |
|  | PTAFR | CMTM8 |  |
|  | GJA5 | NUDT15 |  |
|  | HEYL | SLC1A5 |  |
|  | KLRC2 | CPOX |  |
|  | SGCD | OSGIN2 |  |
|  | RGS13 | FANCG |  |
|  | SECTM1 | CUL4A |  |
|  | S100A8 | AP1AR |  |
|  | GPC6 | SLC25A22 |  |
|  | TBCEL | TRAF4 |  |
|  | BMP2 | LSM8 |  |
|  | CCR2 | SPRING1 |  |
|  | CXCL1 | ELK4 |  |
|  | HSPA12B | BCL7A |  |
|  | EPS8 | CPXM1 |  |
|  | CD207 | TLCD1 |  |
|  | HLA-DRA | ATAD3A |  |
|  | TNFRSF13C | USP43 |  |
|  | JCHAIN | NME2 |  |
|  | CCL25 | HAUS6 |  |
|  | BEND5 | KDM1A |  |
|  | LIPA | OAS3 |  |
|  | SKAP1 | PLSCR1 |  |
|  | CXCL16 | SNRPF |  |
|  | CHIT1 | IQCB1 |  |
|  | CD70 | MRPL13 |  |
|  | BTN3A1 | MRPL50 |  |
|  | CCDC69 | POLR3A |  |
|  | MRC1 | DCK |  |
|  | C11orf21 | SLC25A13 |  |
|  | ZNF835 | ZNF670 |  |
|  | CCDC80 | SEC23B |  |
|  | RNASE6 | ZGRF1 |  |
|  | BST2 | FOXP3 |  |
|  | RIN1 | AGTRAP |  |
|  | STRN | AP1G2 |  |
|  | EIF2AK4 | E2F5 |  |
|  | IL21 | ADAM12 |  |
|  | SPON1 | FBXL6 |  |
|  | INHBE | MSH6 |  |
|  | SLC39A2 | SEL1L3 |  |
|  | ACKR3 | MPHOSPH9 |  |
|  | ULBP2 | NSF |  |
|  | UNC5C | GIT1 |  |
|  | CYFIP2 | ASCC3 |  |
|  | IL18R1 | NCOA6 |  |
|  | CYSLTR2 | ZNF792 |  |
|  | GIMAP2 | ST14 |  |
|  | FCGR3B | ZNF280C |  |
|  | TGFB2 | INTS13 |  |
|  | PAM16 | MLH3 |  |
|  | RAD23B | LRRC45 |  |
|  | ANKRD55 | SFXN1 |  |
|  | TNFSF9 | EXOSC8 |  |
|  | HLA-E | PMS1 |  |
|  | IL23R | RNFT1 |  |
|  | RIN3 | TUBGCP3 |  |
|  | PDIA2 | CD46 |  |
|  | VASH1 | XPNPEP3 |  |
|  | AP3B1 | TSPAN13 |  |
|  | KIR2DS4 | LY75 |  |
|  | ACE | CNPY3 |  |
|  | TSLP | HELB |  |
|  | ARRB2 | RNF6 |  |
|  | CHN1 | THOC1 |  |
|  | ANGPTL2 | BCL2L12 |  |
|  | RFX5 | CHML |  |
|  | TNF | RRS1 |  |
|  | SLC7A7 | RHEBL1 |  |
|  | CLEC3B | ARRDC1 |  |
|  | SULF1 | XPR1 |  |
|  | CLEC6A | TPR |  |
|  | IL5RA | CDH3 |  |
|  | GPR183 | GMCL1 |  |
|  | RAB39A | CTSC |  |
|  | ASXL2 | ADAM8 |  |
|  | ARHGDIB | SH2B3 |  |
|  | TCEAL7 | DEK |  |
|  | FNDC1 | SPRY4 |  |
|  | ADAMTS4 | CEP131 |  |
|  | COL1A2 | NSUN5 |  |
|  | C6orf132 | RLIM |  |
|  | COL5A1 | C1GALT1 |  |
|  | TNFRSF8 | RAB22A |  |
|  | HLA-DQA1 | USP31 |  |
|  | CD300C | CBX8 |  |
|  | RNASE1 | GPRC5A |  |
|  | CLTB | RHPN2 |  |
|  | SARDH | CEP152 |  |
|  | CD3G | CXCL1 |  |
|  | CCL2 | STAMBPL1 |  |
|  | APOBEC3A | MRPS17 |  |
|  | ABI3 | PBX4 |  |
|  | ITM2A | RUVBL2 |  |
|  | LYVE1 | ECE2 |  |
|  | IRF7 | DNAJC22 |  |
|  | ITGA2B | GINS2 |  |
|  | ALDH3B1 | ABRACL |  |
|  | LIME1 | CCNE2 |  |
|  | CORIN | TEDC1 |  |
|  | PDGFC | MFSD10 |  |
|  | ROR1 | XPO4 |  |
|  | MYO1G | ZNF12 |  |
|  | RC3H2 | CD47 |  |
|  | GJA4 | NUP153 |  |
|  | TLR10 | THAP12 |  |
|  | SPRY1 | EXOSC5 |  |
|  | CTSO | UNC93B1 |  |
|  | DPT | DCTPP1 |  |
|  | ALPK2 | RP9 |  |
|  | PLXDC1 | HACD3 |  |
|  | LTBP2 | DOCK6 |  |
|  | GNG7 | TSFM |  |
|  | GPR141 | POMP |  |
|  | MAP7D1 | TXLNG |  |
|  | PARP15 | VRK1 |  |
|  | CXCL3 | CLMN |  |
|  | CHST13 | WASHC5 |  |
|  | SAMSN1 | GPR180 |  |
|  | RASAL3 | SLC25A40 |  |
|  | CD27 | WDR90 |  |
|  | ADPRH | ADGRG1 |  |
|  | ITGB1 | NXT2 |  |
|  | IFNAR2 | WNT2 |  |
|  | CD300A | MTFR1 |  |
|  | TNFAIP8L2 | COL3A1 |  |
|  | CXCL10 | POC1B |  |
|  | IFI44L | NLRC5 |  |
|  | TYROBP | TNFRSF12A |  |
|  | SEC24A | SFXN4 |  |
|  | TGFBR2 | ZNF623 |  |
|  | CCL13 | MRPL15 |  |
|  | CYTIP | PKP4 |  |
|  | FBXL7 | SP140L |  |
|  | ZNF620 | RTKN2 |  |
|  | IL1RL1 | HOXA10 |  |
|  | TRANK1 | UBE2S |  |
|  | NCF1 | PRXL2B |  |
|  | LAPTM5 | ZFYVE27 |  |
|  | SCEL | THNSL1 |  |
|  | OGFRL1 | PIF1 |  |
|  | ANK2 | CDK12 |  |
|  | GPR137B | SLC39A6 |  |
|  | FLI1 | SEZ6L2 |  |
|  | PCDH18 | VOPP1 |  |
|  | VSIG4 | ZNF789 |  |
|  | HTR2A | MAP7 |  |
|  | INHBA | WDR76 |  |
|  | KIR3DL3 | HNF1B |  |
|  | CYP27A1 | NUDT1 |  |
|  | CLEC4G | IER5L |  |
|  | SLC12A3 | SLC17A9 |  |
|  | KCNT2 | ITPR3 |  |
|  | IL19 | GAS2L3 |  |
|  | NOTCH4 | HAPLN3 |  |
|  | PDZRN3 | UBXN11 |  |
|  | KLRC1 | NDOR1 |  |
|  | IL1A | CARNMT1 |  |
|  | PCDHGA12 | ZNFX1 |  |
|  | AQP10 | TMEM132A |  |
|  | MAN1C1 | ANKRD10 |  |
|  | APOBEC3G | HNF4A |  |
|  | VAMP5 | PSRC1 |  |
|  | IL7R | KIAA1841 |  |
|  | LCK | MACC1 |  |
|  | BST1 | DCAF12 |  |
|  | SLURP1 | RNF213 |  |
|  | JAML | NEU3 |  |
|  | GDF5 | DPY19L4 |  |
|  | PRKCB | USP24 |  |
|  | MVP | FCGR1A |  |
|  | AMHR2 | ESCO1 |  |
|  | RIPOR2 | FAM199X |  |
|  | PKIB | RAB15 |  |
|  | NEGR1 | LRP11 |  |
|  | VEGFD | FAP |  |
|  | FAM30A | ACYP1 |  |
|  | LRRC25 | OSBPL10 |  |
|  | DIXDC1 | MICB |  |
|  | TLR4 | HOXC11 |  |
|  | SFN | MLKL |  |
|  | TNFSF11 | RPA3 |  |
|  | PKHD1L1 | LPAR2 |  |
|  | ITGAL | RAI14 |  |
|  | S100A7A | POM121C |  |
|  | CD4 | CMSS1 |  |
|  | MFRP | PTPN6 |  |
|  | VCAN | CREBZF |  |
|  | CCR7 | DLL4 |  |
|  | SECISBP2L | PSMG3 |  |
|  | PRKG1 | HELZ2 |  |
|  | IL16 | PNPO |  |
|  | PLA2G4E | C2 |  |
|  | SOX17 | ZMYM1 |  |
|  | CXCL6 | DIP2B |  |
|  | PMP22 | DDAH1 |  |
|  | TMEM176B | RRBP1 |  |
|  | HIST1H3H | DCLRE1C |  |
|  | BTN3A3 | PMEPA1 |  |
|  | A2M | TATDN1 |  |
|  | FPR1 | ZNF443 |  |
|  | RPS6KA4 | PREP |  |
|  | OLFML2B | ZNF695 |  |
|  | TNFRSF25 | LYPLA1 |  |
|  | FSCN1 | SEC61A2 |  |
|  | NCKAP1L | ASXL1 |  |
|  | SHISAL1 | ADCY3 |  |
|  | TENM3 | TSACC |  |
|  | CCL3 | NFKB2 |  |
|  | KCNK13 | ZNF692 |  |
|  | KL | SMARCA4 |  |
|  | HCLS1 | ABCC10 |  |
|  | ITGA9 | EPHB2 |  |
|  | LY9 | HOXB9 |  |
|  | RASSF4 | RBM39 |  |
|  | GBGT1 | SLC26A6 |  |
|  | LTBR | ANXA4 |  |
|  | PDE3B | RASSF7 |  |
|  | RAMP3 | C18orf54 |  |
|  | AMPD1 | MCTS2P |  |
|  | DHRS9 | AP5Z1 |  |
|  | AKNA | NF1 |  |
|  | HVCN1 | ZNF165 |  |
|  | COL10A1 | SELENOI |  |
|  | KCNN3 | ADGRE5 |  |
|  | TCL1A | ZNF469 |  |
|  | ZBTB32 | VANGL1 |  |
|  | HLX | OTUD6B |  |
|  | XAF1 | TNFSF11 |  |
|  | SIGLEC10 | MARVELD2 |  |
|  | HSP90AA1 | KMT5C |  |
|  | AGTR1 | PTPRJ |  |
|  | RGS5 | IL4I1 |  |
|  | VSIR | CLPB |  |
|  | AFF3 | SBNO1 |  |
|  | XCR1 | RPP40 |  |
|  | TRPV2 | PXYLP1 |  |
|  | GLIS3 | FASTKD1 |  |
|  | SLCO2B1 | MSH5 |  |
|  | ANGPTL1 | GTF3A |  |
|  | KIR2DL1 | SPARC |  |
|  | ENOX1 | SULF1 |  |
|  | GGTA1P | SF3B1 |  |
|  | DOK5 | TK1 |  |
|  | APOE | ZC3H11A |  |
|  | PEG3 | ZNF468 |  |
|  | ENG | ZNF697 |  |
|  | RAPGEF6 | ARFGEF3 |  |
|  | WARS | PLAUR |  |
|  | CXCL5 | C8orf33 |  |
|  | E2F5 | PRELID3B |  |
|  | PIK3R5 | ZFP69B |  |
|  | ZNF423 | BICD1 |  |
|  | CLNK | GRINA |  |
|  | SLAMF6 | CEP95 |  |
|  | IL12B | BCL2L1 |  |
|  | ITGA5 | PDP1 |  |
|  | DCN | CPSF1 |  |
|  | UQCC2 | RBAK |  |
|  | FASLG | MIPEP |  |
|  | PTGDS | PDZD8 |  |
|  | FCER1G | C5orf22 |  |
|  | UHMK1 | GUSB |  |
|  | APLNR | ZNF600 |  |
|  | ZNF660 | ZBTB41 |  |
|  | LIFR | ADAT2 |  |
|  | TNFRSF4 | MRE11 |  |
|  | ETS1 | UGGT2 |  |
|  | JAK3 | TENT4A |  |
|  | CD40 | ABHD17C |  |
|  | CCR10 | PPT1 |  |
|  | PODN | AGPAT5 |  |
|  | HLA-DOB | HOOK1 |  |
|  | CORO1A | TTPAL |  |
|  | S100A9 | AGMAT |  |
|  | MGP | TCF20 |  |
|  | NCOA2 | BEND3 |  |
|  | CAV1 | CELSR3 |  |
|  | DKK2 | SPP1 |  |
|  | PSMD8 | YEATS4 |  |
|  | PPP1R13L | COL1A2 |  |
|  | CREBL2 | TAPBP |  |
|  | ALOX5 | SNRNP200 |  |
|  | SCN7A | MELTF |  |
|  | MPP1 | FAM72D |  |
|  | FZD4 | TRRAP |  |
|  | MAP1LC3C | PLXNA3 |  |
|  | GIMAP6 | INTS2 |  |
|  | ERCC6L2 | PIGM |  |
|  | B2M | PLEKHG4 |  |
|  | KCNK6 | FAM102B |  |
|  | SGIP1 | FNBP1L |  |
|  | SON | HSH2D |  |
|  | ACVR1 | FAM81A |  |
|  | LILRB3 | EFNA1 |  |
|  | PLEKHO1 | POGLUT2 |  |
|  | IL12RB1 | HPDL |  |
|  | RTKN2 | SLC39A7 |  |
|  | RASGRP2 | ITGA2 |  |
|  | APOBEC3H | MDC1 |  |
|  | RAET1L | TCF19 |  |
|  | DDX60 | TMEM41A |  |
|  | ZNF366 | PLOD1 |  |
|  | CTF1 | PKM |  |
|  | TPSD1 | GPR35 |  |
|  | ACKR1 | SNRNP48 |  |
|  | IFNLR1 | KIF3B |  |
|  | MFAP4 | GRHL2 |  |
|  | LSP1 | LCOR |  |
|  | SDCBP2 | ARHGAP18 |  |
|  | PDZK1IP1 | UNG |  |
|  | SNED1 | DBR1 |  |
|  | CCDC102B | LY6E |  |
|  | GAS7 | PARP12 |  |
|  | TGFB3 | CTSA |  |
|  | ICAM1 | TRIM28 |  |
|  | MYCT1 | CMTM7 |  |
|  | HERC6 | TCIRG1 |  |
|  | BHLHE22 | H2AX |  |
|  | SPRR2G | MYO1E |  |
|  | GPR4 | MORC4 |  |
|  | UNC93B1 | RPUSD1 |  |
|  | MAGEL2 | BAX |  |
|  | HS3ST1 | SNRPB2 |  |
|  | IGSF21 | PSMG1 |  |
|  | SLAMF8 | APAF1 |  |
|  | EDNRA | NOL4L |  |
|  | APOL6 | CAND1 |  |
|  | LYL1 | HAGHL |  |
|  | ASGR2 | STK26 |  |
|  | TSPAN4 | IFT80 |  |
|  | AIM2 | TAP2 |  |
|  | HSD17B14 | EIF2AK2 |  |
|  | PSMC1 | BIK |  |
|  | ST3GAL2 | ZFP62 |  |
|  | PLA2G7 | LEF1 |  |
|  | BCL2A1 | RASAL2 |  |
|  | PYHIN1 | KRTCAP3 |  |
|  | STK33 | GTF3C4 |  |
|  | EVI2A | PRKX |  |
|  | GJB3 | MDK |  |
|  | PATZ1 | BAIAP2L1 |  |
|  | ANKRD17 | TIA1 |  |
|  | CLCF1 | ZNF700 |  |
|  | FGD5 | CENPS |  |
|  | TMEM204 | LAGE3 |  |
|  | XCL2 | BLACAT1 |  |
|  | MEF2C | RBM25 |  |
|  | IQGAP2 | KDM1B |  |
|  | SIGLEC6 | SAMD12 |  |
|  | NID2 | PGGHG |  |
|  | HLA-DPB1 | DYRK2 |  |
|  | CD53 | SS18L1 |  |
|  | SH2B3 | MYB |  |
|  | HLA-C | TRIM15 |  |
|  | PNOC | MINPP1 |  |
|  | ITGA8 | SAC3D1 |  |
|  | IL17RB | P3H4 |  |
|  | ASXL3 | SMARCAD1 |  |
|  | MFNG | CYB5B |  |
|  | IFNA13 | PGF |  |
|  | AHCYL2 | LRATD2 |  |
|  | CD84 | DNMT3B |  |
|  | HNMT | PDSS1 |  |
|  | RTP4 | MDFI |  |
|  | TIMM50 | ANKS6 |  |
|  | TGFBI | FNBP4 |  |
|  | MX1 | MMP14 |  |
|  | DDI2 | RAB11FIP4 |  |
|  | ATE1 | STK35 |  |
|  | EPOR | MSR1 |  |
|  | DNAJC5B | TFRC |  |
|  | GAPT | NOTCH3 |  |
|  | CALD1 | PDGFRB |  |
|  | TNFRSF18 | FUT4 |  |
|  | GRIN3A | PCNX3 |  |
|  | CCL5 | PNKD |  |
|  | PPBP | RCBTB1 |  |
|  | ADAMTS2 | ITGA6 |  |
|  | DLL4 | ZRANB2 |  |
|  | ADGRE5 | TUBA1C |  |
|  | SLC6A12 | SERPINE1 |  |
|  | APBB1IP | GPR4 |  |
|  | NAP1L3 | IL18BP |  |
|  | DAAM2 | CTSB |  |
|  | STK17B | ACLY |  |
|  | ABCC9 | MYO6 |  |
|  | RASL12 | HSD3B7 |  |
|  | MICAL2 | ZNF618 |  |
|  | TNFSF15 | AHR |  |
|  | MPEG1 | HENMT1 |  |
|  | C1QC | ZDHHC13 |  |
|  | MRPS12 | OLFML2B |  |
|  | FKBP7 | P4HA3 |  |
|  | BMPR1A | LIPG |  |
|  | CLEC11A | CCNI2 |  |
|  | CX3CR1 | PARD6B |  |
|  | EPSTI1 | GMIP |  |
|  | MEDAG | RRM2B |  |
|  | BMPR2 | TMC5 |  |
|  | CCR5 | FRMD5 |  |
|  | ARHGAP4 | GFPT1 |  |
|  | CR2 | RNASET2 |  |
|  | SOWAHD | C2CD4D |  |
|  | CMKLR1 | APOE |  |
|  | FCRL6 | STX16 |  |
|  | EGF | UBD |  |
|  | ADGRE2 | RDM1 |  |
|  | CCR3 | PRKAA1 |  |
|  | NCR1 | KBTBD6 |  |
|  | MXRA8 | HOXC8 |  |
|  | CSF2 | ENO1 |  |
|  | RASGRP4 | SIPA1L3 |  |
|  | PIM2 | SCML1 |  |
|  | REL | ZBED6CL |  |
|  | FOLR2 | QSER1 |  |
|  | NCF2 | TMEM97 |  |
|  | EHD2 | KIAA1217 |  |
|  | SEM1 | SLC45A4 |  |
|  | PTPRCAP | UBA6 |  |
|  | FERMT2 | ZMYND15 |  |
|  | JAM3 | MMP9 |  |
|  | ITGA4 | MYO7A |  |
|  | COL1A1 | HAUS5 |  |
|  | PLCB2 | HYOU1 |  |
|  | SNRPF | SLC7A6 |  |
|  | FCN1 | LASP1 |  |
|  | SIGLEC14 | MAPK15 |  |
|  | AP1S2 | ARL5B |  |
|  | NR1H3 | TP53I11 |  |
|  | OMD | FGD6 |  |
|  | PKD2L1 | RALGAPA2 |  |
|  | IL1R2 | NPL |  |
|  | CSMD2 | OGT |  |
|  | CACNA1C | HAVCR2 |  |
|  | BMPR1B | P2RY6 |  |
|  | GNAI2 | LRRC61 |  |
|  | TMEM156 | PAN3 |  |
|  | LILRB4 | NRM |  |
|  | TPSB2 | ARAP3 |  |
|  | S1PR4 | SLC35B2 |  |
|  | ADAMDEC1 | GRB7 |  |
|  | TOX | PDGFB |  |
|  | CNFN | VCAN |  |
|  | TIGIT | IQCE |  |
|  | NUGGC | LAMB1 |  |
|  | PDGFA | FNDC1 |  |
|  | TM4SF18 | LACTB2 |  |
|  | UNC13D | SKIL |  |
|  | ADGRF5 | CHST1 |  |
|  | CPA3 | TMSB10 |  |
|  | CD274 | REPIN1 |  |
|  | RCSD1 | HOXA13 |  |
|  | SIGLEC5 | ANKRD52 |  |
|  | CCL26 | MRPS35 |  |
|  | CCR8 | ANKRD13B |  |
|  | SLC25A53 | TMEM241 |  |
|  | MARCO | MXRA5 |  |
|  | IKZF1 | CDK4 |  |
|  | PHACTR1 | EPSTI1 |  |
|  | ITGA1 | ELF3 |  |
|  | ADAM6 | CEP295 |  |
|  | TAP1 | DHFR |  |
|  | ARHGAP30 | GGH |  |
|  | PRKAR2A | IGFBP3 |  |
|  | ARHGEF37 | DRAM1 |  |
|  | CLEC4D | CHI3L1 |  |
|  | TEK | VASH1 |  |
|  | LGMN | MYO5C |  |
|  | SULT1C4 | MROH1 |  |
|  | HLA-DRB5 | FAT1 |  |
|  | DOCK11 | OR2I1P |  |
|  | LILRP2 | FXYD5 |  |
|  | CD37 | JAG2 |  |
|  | CR1 | CHSY3 |  |
|  | HIST1H2AM | CLDN12 |  |
|  | ZNF683 | URB1 |  |
|  | ANKRD44 | HCFC1 |  |
|  | GIMAP1 | THBS2 |  |
|  | IL2RA | MAP3K1 |  |
|  | APOL3 | MFAP2 |  |
|  | IPCEF1 | PRR5L |  |
|  | CMAHP | EPS8L3 |  |
|  | VEGFC | APLN |  |
|  | BNC2 | PPP1R35 |  |
|  | SIRPB2 | RTEL1-TNFRSF6B |  |
|  | SP140 | RSKR |  |
|  | GATA3 | FBXO41 |  |
|  | CCL14 | COL12A1 |  |
|  | NFKBID | WRN |  |
|  | CTSK | ZNF251 |  |
|  | KRT6B | RB1 |  |
|  | LILRA2 | NPHP4 |  |
|  | IKZF3 | LRP6 |  |
|  | TLR8 | FAM117B |  |
|  | CLEC10A | SPATA2 |  |
|  | SELL | RELB |  |
|  | MRO | PABPC1 |  |
|  | LATS2 | CAPN15 |  |
|  | COL8A1 | SLAMF8 |  |
|  | GPR157 | ASAP1 |  |
|  | CPZ | LBX2 |  |
|  | RTRAF | CXCL8 |  |
|  | IL6R | ZNF318 |  |
|  | USP51 | FJX1 |  |
|  | CARD9 | CEP192 |  |
|  | IL1RAP | PIGO |  |
|  | TNFRSF10D | CDCP1 |  |
|  | TTC16 | KDELR3 |  |
|  | CXCL12 | MAL2 |  |
|  | C10orf99 | SLC52A3 |  |
|  | GVINP1 | LAMB3 |  |
|  | GFRA3 | SCRIB |  |
|  | MAN1A2 | CHRNA5 |  |
|  | CD8B | SPINDOC |  |
|  | IL6ST | CEP170 |  |
|  | NRXN3 | MDN1 |  |
|  | IL17A | TDRKH |  |
|  | IL15 | GPR160 |  |
|  | NDNF | TYMP |  |
|  | EPO | RIN2 |  |
|  | CCL4 | SCARB1 |  |
|  | EDA | GNS |  |
|  | LCE3D | ZC3HAV1L |  |
|  | CD5 | GDPD5 |  |
|  | SH2D3C | ICA1 |  |
|  | GZMB | POFUT1 |  |
|  | GRAP2 | SLC9A8 |  |
|  | GPRIN3 | DSG2 |  |
|  | GATA2 | MRPL12 |  |
|  | ADGRD1 | SMG1 |  |
|  | SH2D2A | SLC4A11 |  |
|  | GHR | RNF207 |  |
|  | CXCR5 | ANO9 |  |
|  | SLC2A5 | CARD10 |  |
|  | PARP12 | HPSE |  |
|  | PIP4K2A | CLCN2 |  |
|  | TNFSF13B | JPT1 |  |
|  | PTGIR | PAXBP1 |  |
|  | IRF9 | ARFGEF2 |  |
|  | C2 | TLCD3A |  |
|  | IL2RG | KLF16 |  |
|  | GNG11 | SLC25A10 |  |
|  | TNS3 | CERKL |  |
|  | TMEM176A | MCTP2 |  |
|  | EIF2A | LPGAT1 |  |
|  | PPL | KIF2A |  |
|  | KIRREL1 | C10orf95 |  |
|  | S100A16 | CXCL9 |  |
|  | COL4A2 | SLC30A1 |  |
|  | NFYA | ZNF888 |  |
|  | IFI6 | HIF1A |  |
|  | PSME3 | HKDC1 |  |
|  | S100A2 | STK36 |  |
|  | C19orf38 | ARHGEF39 |  |
|  | ULBP3 | FCGR2A |  |
|  | LY96 | NUP62CL |  |
|  | GJB5 | REEP4 |  |
|  | DUSP16 | PLCH1 |  |
|  | RAB42 | UPF3B |  |
|  | CD86 | RAB19 |  |
|  | C5AR1 | METTL7B |  |
|  | ADAMTS5 | RTN4R |  |
|  | ANTXR1 | HOXC6 |  |
|  | LIF | KIF12 |  |
|  | ART4 | XYLB |  |
|  | P2RY2 | ANKIB1 |  |
|  | GNA15 | SEM1 |  |
|  | CDSN | SRC |  |
|  | P2RX5 | PRR15L |  |
|  | CNRIP1 | ARHGAP12 |  |
|  | IFITM3 | FAM167B |  |
|  | PDGFRL | PTK7 |  |
|  | FILIP1L | CSGALNACT2 |  |
|  | CCL17 | STX3 |  |
|  | TNIP3 | ZFP41 |  |
|  | SLA2 | COL5A1 |  |
|  | ADGRE4P | NECTIN2 |  |
|  | DOK2 | MED1 |  |
|  | ULBP1 | IL32 |  |
|  | CXCR4 | UBR5 |  |
|  | PSMD7 | CD2AP |  |
|  | TBXA2R | SLC19A1 |  |
|  | ST6GALNAC3 | PMAIP1 |  |
|  | AEBP1 | ZNF816 |  |
|  | CTSE | ADCK5 |  |
|  | FLVCR2 | CBX2 |  |
|  | ASPN | DENND2D |  |
|  | PCED1B | PCMTD2 |  |
|  | IFNL1 | PLPP2 |  |
|  | GALNT15 | HMGB2 |  |
|  | HAVCR1 | TLR2 |  |
|  | EFEMP2 | PHLDA2 |  |
|  | FAM177B | ZNF28 |  |
|  | HLA-DMB | HK2 |  |
|  | ITGA11 | MUC13 |  |
|  | ZC3H12D | KLHL17 |  |
|  | C1orf116 | PTPRE |  |
|  | GPR15 | SMAD6 |  |
|  | COL6A5 | HOXC10 |  |
|  | CCL18 | TNFRSF4 |  |
|  | HRH2 | ZNF860 |  |
|  | HSPA8 | SPR |  |
|  | RNF166 | WWC1 |  |
|  | ASAH1 | SLC39A14 |  |
|  | OSM | ENGASE |  |
|  | SLIT3 | PSMD3 |  |
|  | MS4A6A | KRT8 |  |
|  | PEAK3 | CENPJ |  |
|  | PSMB9 | TNFRSF9 |  |
|  | SLC11A1 | PRSS22 |  |
|  | PTGDR | TNFSF15 |  |
|  | HLA-DOA | THEM4 |  |
|  | PSME1 | DOCK5 |  |
|  | LRRC17 | ERBB3 |  |
|  | ZNF469 | ADAMTS7 |  |
|  | CTSB | SH2D3A |  |
|  | RASSF6 | TRIB3 |  |
|  | MS4A1 | CBLC |  |
|  | ZNF521 | NR6A1 |  |
|  | TLR9 | LOX |  |
|  | CD1D | CCNE1 |  |
|  | ZNF827 | NBEAL2 |  |
|  | DMXL2 | MEX3A |  |
|  | OGN | GPR176 |  |
|  | GTF2A1 | HOXA11 |  |
|  | MMP12 | MOCOS |  |
|  | ACHE | HIP1 |  |
|  | KLHL11 | LAD1 |  |
|  | MMP16 | CCL3 |  |
|  | ADCYAP1 | PLEK2 |  |
|  | OLR1 | PLXND1 |  |
|  | RAB39B | SLC35F2 |  |
|  | APBB2 | NCOA7 |  |
|  | ENTPD1 | MYRF |  |
|  | ADGRG5 | SH3BP4 |  |
|  | MMP9 | BAIAP2L2 |  |
|  | RGPD1 | ACBD5 |  |
|  | HPGD | MSX2 |  |
|  | TTC24 | TRIM31 |  |
|  | TCN2 | RNF24 |  |
|  | SASH3 | TAP1 |  |
|  | POSTN | SAMD10 |  |
|  | HSH2D | RNF32 |  |
|  | CD19 | NCR3LG1 |  |
|  | ZBTB10 | DDR1 |  |
|  | ARHGAP15 | CCNL2 |  |
|  | NIPAL4 | KLF5 |  |
|  | RUNX3 | CD300LF |  |
|  | PIEZO2 | CLDN3 |  |
|  | KCNJ10 | TRAF5 |  |
|  | GPBAR1 | OSCAR |  |
|  | GPR34 | F2RL2 |  |
|  | ARRDC5 | TMEM38B |  |
|  | MMP1 | FAR2 |  |
|  | IFNAR1 | CEP250 |  |
|  | MICB | KIAA1549 |  |
|  | IFI30 | HNF4G |  |
|  | FAM83A-AS1 | CCR8 |  |
|  | GIMAP4 | ONECUT2 |  |
|  | CCL22 | TNFRSF25 |  |
|  | CARD11 | OSMR |  |
|  | DCANP1 | SLC39A8 |  |
|  | CDKL5 | GCC2 |  |
|  | AOAH | EPS8 |  |
|  | JAKMIP1 | TRIM24 |  |
|  | SUCNR1 | LILRB4 |  |
|  | MRPL55 | OCLN |  |
|  | HLA-DQA2 | RCN3 |  |
|  | KLRC4 | PCNX2 |  |
|  | CANX | FAAH2 |  |
|  | TRAF3IP3 | PRKCI |  |
|  | IL6 | SLC18B1 |  |
|  | CETP | NPM3 |  |
|  | TNFSF10 | KIF21B |  |
|  | C1QB | CST2 |  |
|  | IRF1 | SLC11A1 |  |
|  | NAIP | FASN |  |
|  | P2RY14 | CETP |  |
|  | MMRN1 | CDK6 |  |
|  | SAMD9 | PFDN4 |  |
|  | CCL24 | BTN3A2 |  |
|  | CD6 | PCDH12 |  |
|  | ACP5 | TMEM168 |  |
|  | TNFRSF1A | NPC1 |  |
|  | GPR78 | IRF8 |  |
|  | AREG | CXCL10 |  |
|  | GATA1 | IFITM3 |  |
|  | CAMK4 | SPIN4 |  |
|  | CD52 | PLEKHG1 |  |
|  | IFNB1 | LFNG |  |
|  | ZNF804A | VEGFA |  |
|  | SFTPB | CPD |  |
|  | SNX20 | CHRNA1 |  |
|  | ROCK2 | EMILIN2 |  |
|  | HK3 | C2CD4A |  |
|  | SAMD14 | TRIM10 |  |
|  | MR1 | SLC5A3 |  |
|  | ITGB3 | CLEC5A |  |
|  | HIPK3 | LRRCC1 |  |
|  | LMOD1 | MMP1 |  |
|  | B3GAT1 | LMTK2 |  |
|  | POU2AF1 | ODC1 |  |
|  | AOC3 | ESPL1 |  |
|  | PLEKHN1 | IGSF6 |  |
|  | PUS10 | FOXP4 |  |
|  | KIR3DL2 | MDM2 |  |
|  | TIE1 | ERMP1 |  |
|  | TREM2 | H2AC17 |  |
|  | FPR2 | COTL1 |  |
|  | THBS2 | CTSS |  |
|  | SOD3 | USH1C |  |
|  | CLEC1A | TEX30 |  |
|  | TBX21 | C4orf48 |  |
|  | CXCL13 | BATF |  |
|  | STARD8 | DDX39B |  |
|  | IFFO1 | PABPC3 |  |
|  | CST7 | IFI6 |  |
|  | CERKL | PTPRG |  |
|  | TAPBP | APOBEC3B |  |
|  | RRN3P2 | KIFC2 |  |
|  | CYSRT1 | OAS2 |  |
|  | LMTK2 | PILRB |  |
|  | CD226 | PIEZO1 |  |
|  | LGI2 | STEAP1 |  |
|  | PSMD4 | CGN |  |
|  | CSF2RB | FAM110A |  |
|  | TNFRSF9 | ZNF69 |  |
|  | CPED1 | CD80 |  |
|  | TPSAB1 | COL5A3 |  |
|  | CCL15 | RAB42 |  |
|  | SELP | MYO10 |  |
|  | NOX4 | MAPK13 |  |
|  | DUOXA1 | CHPF |  |
|  | ITK | COL11A1 |  |
|  | RGS18 | PCGF2 |  |
|  | TNFRSF11A | COL7A1 |  |
|  | CYTH4 | SEMA4G |  |
|  | TNN | ZNF518A |  |
|  | SLC45A3 | NUAK1 |  |
|  | FICD | C1orf198 |  |
|  | MXD1 | TMEM164 |  |
|  | IL15RA | COL18A1 |  |
|  | SDS | SEC61G |  |
|  | BATF3 | ABCA7 |  |
|  |  | MGAT4B |  |
|  |  | RNF43 |  |
|  |  | CXCL3 |  |
|  |  | ARNTL2 |  |
|  |  | AGAP6 |  |
|  |  | CNKSR1 |  |
|  |  | PIWIL4 |  |
|  |  | GALNT3 |  |
|  |  | TNRC18 |  |
|  |  | EVA1A |  |
|  |  | BCL11B |  |
|  |  | INAVA |  |
|  |  | PSD4 |  |
|  |  | POMK |  |
|  |  | FOXRED2 |  |
|  |  | CCNO |  |
|  |  | NOX4 |  |
|  |  | CELSR1 |  |
|  |  | ITPRID2 |  |
|  |  | ADAP2 |  |
|  |  | CDH11 |  |
|  |  | ABCC3 |  |
|  |  | LZTS1 |  |
|  |  | FZD2 |  |
|  |  | AP1M2 |  |
|  |  | KCNAB2 |  |
|  |  | PLEKHS1 |  |
|  |  | FOXL1 |  |
|  |  | DAGLA |  |
|  |  | GTF2IRD1 |  |
|  |  | WDR72 |  |
|  |  | CXCL11 |  |
|  |  | MIF |  |
|  |  | PXDN |  |
|  |  | ICAM1 |  |
|  |  | STC2 |  |
|  |  | FRK |  |
|  |  | CACNA1D |  |
|  |  | FAM89A |  |
|  |  | PODNL1 |  |
|  |  | TRIO |  |
|  |  | CCND1 |  |
|  |  | STK32C |  |
|  |  | KCNE3 |  |
|  |  | HOXB7 |  |
|  |  | NOTCH4 |  |
|  |  | ARHGAP26 |  |
|  |  | IL27RA |  |
|  |  | KRT80 |  |
|  |  | SOD2 |  |
|  |  | BATF2 |  |
|  |  | ZNF724 |  |
|  |  | SLC7A1 |  |
|  |  | SALL4 |  |
|  |  | IRF7 |  |
|  |  | SNAI1 |  |
|  |  | PKP2 |  |
|  |  | SLC43A3 |  |
|  |  | C3orf52 |  |
|  |  | CYBA |  |
|  |  | GRAMD1A |  |
|  |  | VIL1 |  |
|  |  | IL23A |  |
|  |  | STRA6 |  |
|  |  | E2F2 |  |
|  |  | AP1S1 |  |
|  |  | CLSTN3 |  |
|  |  | ETS1 |  |
|  |  | ITGA11 |  |
|  |  | FKBP10 |  |
|  |  | PRR19 |  |
|  |  | HRH1 |  |
|  |  | KDF1 |  |
|  |  | RSRP1 |  |
|  |  | SLC35E4 |  |
|  |  | TNFAIP2 |  |
|  |  | C2orf72 |  |
|  |  | NETO2 |  |
|  |  | TINAGL1 |  |
|  |  | IL2RA |  |
|  |  | CRACR2B |  |
|  |  | MMP12 |  |
|  |  | ADGRF5 |  |
|  |  | EFNA3 |  |
|  |  | RESF1 |  |
|  |  | MICALL2 |  |
|  |  | MEX3D |  |
|  |  | PRR15 |  |
|  |  | ZKSCAN1 |  |
|  |  | EGFL6 |  |
|  |  | EPHA10 |  |
|  |  | KRTAP5-1 |  |
|  |  | GPR84 |  |
|  |  | LUC7L3 |  |
|  |  | OCIAD2 |  |
|  |  | C1orf226 |  |
|  |  | RGS19 |  |
|  |  | PKN3 |  |
|  |  | SMKR1 |  |
|  |  | COL6A3 |  |
|  |  | MATN3 |  |
|  |  | STC1 |  |
|  |  | ZNF841 |  |
|  |  | SATB2 |  |
|  |  | TRIB2 |  |
|  |  | ACBD7 |  |
|  |  | IGF2BP3 |  |
|  |  | EPOP |  |
|  |  | SPTBN1 |  |
|  |  | CLCN4 |  |
|  |  | PFKFB2 |  |
|  |  | BCL2A1 |  |
|  |  | CABLES2 |  |
|  |  | MCIDAS |  |
|  |  | PON2 |  |
|  |  | HIP1R |  |
|  |  | FSCN1 |  |
|  |  | TC2N |  |
|  |  | ADGRE2 |  |
|  |  | TNFSF4 |  |
|  |  | SRMS |  |
|  |  | JAK3 |  |
|  |  | ERFE |  |
|  |  | CCN4 |  |
|  |  | NEK3 |  |
|  |  | SULF2 |  |
|  |  | PTPRK |  |
|  |  | ARHGAP4 |  |
|  |  | PLXDC1 |  |
|  |  | PLEKHG2 |  |
|  |  | ZNF525 |  |
|  |  | GLS |  |
|  |  | ERO1A |  |
|  |  | SPON2 |  |
|  |  | CHMP4C |  |
|  |  | ELOVL7 |  |
|  |  | FCER1G |  |
|  |  | ISG15 |  |
|  |  | KCNC3 |  |
|  |  | SPHK1 |  |
|  |  | HEYL |  |
|  |  | MYH14 |  |
|  |  | ZNF711 |  |
|  |  | UBASH3B |  |
|  |  | FAM221A |  |
|  |  | COL27A1 |  |
|  |  | IFIH1 |  |
|  |  | C1orf53 |  |
|  |  | MMP7 |  |
|  |  | DHCR7 |  |
|  |  | FMNL3 |  |
|  |  | PAX8 |  |
|  |  | ASPHD1 |  |
|  |  | ARSL |  |
|  |  | SLC2A6 |  |
|  |  | NID2 |  |
|  |  | KRIT1 |  |
|  |  | GPX8 |  |
|  |  | IFITM10 |  |
|  |  | SNORC |  |
|  |  | ZBED6 |  |
|  |  | NHS |  |
|  |  | CTLA4 |  |
|  |  | SEPHS2 |  |
|  |  | BTBD19 |  |
|  |  | GLB1L |  |
|  |  | RCOR2 |  |
|  |  | SFRP4 |  |
|  |  | CNNM4 |  |
|  |  | MROH6 |  |
|  |  | WARS1 |  |
|  |  | ADAMDEC1 |  |
|  |  | ETV5 |  |
|  |  | CPNE7 |  |
|  |  | SYNE2 |  |
|  |  | ITGB8 |  |
|  |  | ITGB1 |  |
|  |  | ACP5 |  |
|  |  | PLAGL2 |  |
|  |  | PKDCC |  |
|  |  | TNFRSF11B |  |
|  |  | ANOS1 |  |
|  |  | ARHGEF16 |  |
|  |  | FLVCR1 |  |
|  |  | GSTO2 |  |
|  |  | LBH |  |
|  |  | MMP3 |  |
|  |  | LCP2 |  |
|  |  | DIO2 |  |
|  |  | PSAT1 |  |
|  |  | RPS6KA1 |  |
|  |  | ALKAL1 |  |
|  |  | HMGA2 |  |
|  |  | CTSL |  |
|  |  | PCDH17 |  |
|  |  | FEZF1 |  |
|  |  | TMPRSS4 |  |
|  |  | LINGO1 |  |
|  |  | STK31 |  |
|  |  | MSC |  |
|  |  | PPM1N |  |
|  |  | BAMBI |  |
|  |  | LEMD1 |  |
|  |  | RIN1 |  |
|  |  | IRAK2 |  |
|  |  | SH2D4A |  |
|  |  | SLC44A3 |  |
|  |  | KLHL35 |  |
|  |  | SLC27A2 |  |
|  |  | RTN4RL2 |  |
|  |  | NRP1 |  |
|  |  | TRABD2A |  |
|  |  | CLRN3 |  |
|  |  | PLS1 |  |
|  |  | LAPTM4B |  |
|  |  | LPIN3 |  |
|  |  | MSLN |  |
|  |  | MCF2L |  |
|  |  | PDX1 |  |
|  |  | NEIL2 |  |
|  |  | COL8A1 |  |
|  |  | SLC7A7 |  |
|  |  | CPLX1 |  |
|  |  | MMP10 |  |
|  |  | ENG |  |
|  |  | ZBTB12 |  |
|  |  | F12 |  |
|  |  | TFAP2A |  |
|  |  | FAM24B |  |
|  |  | GPRIN1 |  |
|  |  | PPFIA3 |  |
|  |  | IL11 |  |
|  |  | GALM |  |
|  |  | SLCO4A1 |  |
|  |  | ZNF738 |  |
|  |  | NRARP |  |
|  |  | EFNB1 |  |
|  |  | BNIP5 |  |
|  |  | HK3 |  |
|  |  | SLC16A3 |  |
|  |  | PKP3 |  |
|  |  | ESRP2 |  |
|  |  | LARGE2 |  |
|  |  | XAF1 |  |
|  |  | APOBR |  |
|  |  | SMAGP |  |
|  |  | CD83 |  |
|  |  | UGT8 |  |
|  |  | IRF6 |  |
|  |  | ZNF320 |  |
|  |  | CATSPERB |  |
|  |  | NMB |  |
|  |  | SLC28A3 |  |
|  |  | BTBD16 |  |
|  |  | SKAP1 |  |
|  |  | RAB25 |  |
|  |  | PLEKHG5 |  |
|  |  | SIX4 |  |
|  |  | CD86 |  |
|  |  | URI1 |  |
|  |  | PLXNB1 |  |
|  |  | H2BC17 |  |
|  |  | POU5F1 |  |
|  |  | FOXC1 |  |
|  |  | PCSK9 |  |
|  |  | S100A10 |  |
|  |  | IFITM1 |  |
|  |  | ADAM9 |  |
|  |  | CREB3L1 |  |
|  |  | EIF4EBP1 |  |
|  |  | CXADR |  |
|  |  | EHF |  |
|  |  | OXTR |  |
|  |  | ODF3B |  |
|  |  | HES6 |  |
|  |  | DMBX1 |  |
|  |  | KLHL11 |  |
|  |  | TMEM158 |  |
|  |  | TMEM74B |  |
|  |  | COL4A2 |  |
|  |  | GBP5 |  |
|  |  | TAFA5 |  |
|  |  | HLA-B |  |
|  |  | ADGRG5 |  |
|  |  | METTL27 |  |
|  |  | MST1R |  |
|  |  | AMH |  |
|  |  | GPRIN3 |  |
|  |  | PLA1A |  |
|  |  | NOS3 |  |
|  |  | MANEAL |  |
|  |  | CDH1 |  |
|  |  | CTSV |  |
|  |  | TNFSF13B |  |
|  |  | ELMO3 |  |
|  |  | LGALS9 |  |
|  |  | FPR3 |  |
|  |  | MGAM2 |  |
|  |  | CCL15 |  |
|  |  | GPR39 |  |
|  |  | KREMEN2 |  |
|  |  | RPL22L1 |  |
|  |  | ADAMTSL2 |  |
|  |  | CILP2 |  |
|  |  | NOTCH1 |  |
|  |  | MSX1 |  |
|  |  | WNT5A |  |
|  |  | CTSH |  |
|  |  | SPIRE2 |  |
|  |  | CD72 |  |
|  |  | MTMR11 |  |
|  |  | ASCL2 |  |
|  |  | STEAP2 |  |
|  |  | TCF7 |  |
|  |  | CCL3L1 |  |
|  |  | S100A11 |  |
|  |  | CD84 |  |
|  |  | IDO1 |  |
|  |  | PTPRU |  |
|  |  | SDC4 |  |
|  |  | PPP1R13L |  |
|  |  | TMPRSS3 |  |
|  |  | KRT17 |  |
|  |  | TSPAN8 |  |
|  |  | POF1B |  |
|  |  | CXCL6 |  |
|  |  | IBSP |  |
|  |  | CST4 |  |
|  |  | S100A3 |  |
|  |  | LGR4 |  |
|  |  | CLDN2 |  |
|  |  | SERINC2 |  |
|  |  | APOL4 |  |
|  |  | JUP |  |
|  |  | C4BPA |  |
|  |  | GJB1 |  |
|  |  | FZD6 |  |
|  |  | PILRA |  |
|  |  | APLNR |  |
|  |  | KLHL23 |  |
|  |  | RNASE2 |  |
|  |  | PLBD1 |  |
|  |  | SOX12 |  |
|  |  | TMEM176B |  |
|  |  | LGR5 |  |
|  |  | SERPINB9 |  |
|  |  | RAB11FIP1 |  |
|  |  | ULBP2 |  |
|  |  | GAD1 |  |
|  |  | TOR4A |  |
|  |  | SPINT1 |  |
|  |  | SLC2A1 |  |
|  |  | LAPTM5 |  |
|  |  | CARD11 |  |
|  |  | SQLE |  |
|  |  | CCL4L2 |  |
|  |  | CALML4 |  |
|  |  | PRKCG |  |
|  |  | TNFRSF21 |  |
|  |  | TDRD5 |  |
|  |  | RIBC2 |  |
|  |  | MAMDC4 |  |
|  |  | RNF128 |  |
|  |  | CCR1 |  |
|  |  | RGS16 |  |
|  |  | KRT7 |  |
|  |  | CRACD |  |
|  |  | AGT |  |
|  |  | CCNP |  |
|  |  | ETV7 |  |
|  |  | CLEC7A |  |
|  |  | GOLGA8A |  |
|  |  | ABCC4 |  |
|  |  | KCNN4 |  |
|  |  | MUC3A |  |
|  |  | SUCNR1 |  |
|  |  | PLXNC1 |  |
|  |  | IFI44 |  |
|  |  | DUSP4 |  |
|  |  | ZP3 |  |
|  |  | ABHD2 |  |
|  |  | GJB3 |  |
|  |  | HOXB13 |  |
|  |  | RARRES1 |  |
|  |  | NECTIN1 |  |
|  |  | TOX3 |  |
|  |  | GJB4 |  |
|  |  | CFB |  |
|  |  | NREP |  |
|  |  | CSF2 |  |
|  |  | GAL3ST2 |  |
|  |  | PSMB9 |  |
|  |  | TMC4 |  |
|  |  | ANKS4B |  |
|  |  | FYB1 |  |
|  |  | STX1A |  |
|  |  | NUAK2 |  |
|  |  | RUNX3 |  |
|  |  | ULBP3 |  |
|  |  | TMEM200A |  |
|  |  | RASEF |  |
|  |  | LAIR1 |  |
|  |  | GASK1B |  |
|  |  | ASRGL1 |  |
|  |  | TNNC1 |  |
|  |  | GSDMB |  |
|  |  | BCAT1 |  |
|  |  | TLX1 |  |
|  |  | HLA-F |  |
|  |  | KCNK1 |  |
|  |  | CT83 |  |
|  |  | CD55 |  |
|  |  | HOXB6 |  |
|  |  | BSPRY |  |
|  |  | ARHGEF19 |  |
|  |  | GPX2 |  |
|  |  | IFIT3 |  |
|  |  | COMP |  |
|  |  | ASPN |  |
|  |  | FAM83B |  |
|  |  | TMEM176A |  |
|  |  | SYNDIG1 |  |
|  |  | MMP15 |  |
|  |  | RASGEF1A |  |
|  |  | CDX2 |  |
|  |  | BIRC7 |  |
|  |  | CXCR4 |  |
|  |  | F5 |  |
|  |  | CCL20 |  |
|  |  | PRSS51 |  |
|  |  | CDKN2A |  |
|  |  | GATA3 |  |
|  |  | TM4SF1 |  |
|  |  | VMO1 |  |
|  |  | M1AP |  |
|  |  | F2RL1 |  |
|  |  | CAPN12 |  |
|  |  | B3GNT3 |  |
|  |  | FN1 |  |
|  |  | PLK2 |  |
|  |  | OSM |  |
|  |  | SERPINA1 |  |
|  |  | MYEOV |  |
|  |  | ITGB4 |  |
|  |  | ARL9 |  |
|  |  | TNNI3 |  |
|  |  | SLC6A14 |  |
|  |  | SLC44A4 |  |
|  |  | HHEX |  |
|  |  | C4BPB |  |
|  |  | TMEM139 |  |
|  |  | NEB |  |
|  |  | GLIS3 |  |
|  |  | HES4 |  |
|  |  | LRRC32 |  |
|  |  | NEURL3 |  |
|  |  | COL22A1 |  |
|  |  | SPACA4 |  |
|  |  | TMEM125 |  |
|  |  | IL2RG |  |
|  |  | H2BC9 |  |
|  |  | NOX1 |  |
|  |  | ISYNA1 |  |
|  |  | IYD |  |
|  |  | GATA6 |  |
|  |  | IFI44L |  |
|  |  | SIX1 |  |
|  |  | TMC8 |  |
|  |  | LCN12 |  |
|  |  | BST2 |  |
|  |  | EOMES |  |
|  |  | SEMA7A |  |
|  |  | PRAME |  |
|  |  | SERPINB5 |  |
|  |  | FGFRL1 |  |
|  |  | PIP5K1B |  |
|  |  | SPDYC |  |
|  |  | SLC43A1 |  |
|  |  | TMEM238 |  |
|  |  | FOXA2 |  |
|  |  | ZIC2 |  |
|  |  | IL24 |  |
|  |  | S100A6 |  |
|  |  | PRAC2 |  |
|  |  | LAMA5 |  |
|  |  | OPLAH |  |
|  |  | VDR |  |
|  |  | DKK2 |  |
|  |  | LRRC15 |  |
|  |  | SIGLEC10 |  |
|  |  | DUSP2 |  |
|  |  | CCL7 |  |
|  |  | CAMSAP3 |  |
|  |  | COCH |  |
|  |  | PROCR |  |
|  |  | CCL18 |  |
|  |  | IFIT2 |  |
|  |  | SRCIN1 |  |
|  |  | CYBB |  |
|  |  | LCK |  |
|  |  | KLHDC7B |  |
|  |  | SLC7A11 |  |
|  |  | IGFBP7 |  |
|  |  | LUM |  |
|  |  | MYOM3 |  |
|  |  | VCAM1 |  |
|  |  | EPPK1 |  |
|  |  | MYOF |  |
|  |  | FABP6 |  |
|  |  | LAIR2 |  |
|  |  | ST6GAL1 |  |
|  |  | FOXH1 |  |
|  |  | EDN1 |  |
|  |  | ADM2 |  |
|  |  | PRRX1 |  |
|  |  | NXPH4 |  |
|  |  | KRT19 |  |
|  |  | TNFSF9 |  |
|  |  | SCD |  |
|  |  | NKD2 |  |
|  |  | TGFBI |  |
|  |  | NR5A2 |  |
|  |  | HOXA9 |  |
|  |  | CCL4 |  |
|  |  | EPHA1 |  |
|  |  | C3AR1 |  |
|  |  | MEGF6 |  |
|  |  | CD24 |  |
|  |  | H2BC11 |  |
|  |  | TMEM45B |  |
|  |  | OVOL1 |  |
|  |  | SPTBN2 |  |
|  |  | H4C9 |  |
|  |  | MMP13 |  |
|  |  | ZNF117 |  |
|  |  | EPHA2 |  |
|  |  | PROC |  |
|  |  | SYT13 |  |
|  |  | HS3ST2 |  |
|  |  | GBP4 |  |
|  |  | IL13RA2 |  |
|  |  | SLC12A2 |  |
|  |  | TJP3 |  |
|  |  | SMIM22 |  |
|  |  | LAMA3 |  |
|  |  | TREM1 |  |
|  |  | GGT5 |  |
|  |  | C4B |  |
|  |  | FOXA3 |  |
|  |  | SLFN13 |  |
|  |  | SLC1A3 |  |
|  |  | ARID3A |  |
|  |  | FSTL3 |  |
|  |  | HID1 |  |
|  |  | LZTS3 |  |
|  |  | ITGB2 |  |
|  |  | ZNF703 |  |
|  |  | BAAT |  |
|  |  | PLAAT3 |  |
|  |  | ZIC5 |  |
|  |  | MLXIPL |  |
|  |  | EFNA2 |  |
|  |  | TLDC2 |  |
|  |  | ATP6V1C2 |  |
|  |  | ASS1 |  |
|  |  | PTPRO |  |
|  |  | ATP7B |  |
|  |  | PLEK |  |
|  |  | CDH17 |  |
|  |  | ERN2 |  |
|  |  | KLK6 |  |
|  |  | AOC1 |  |
|  |  | CHST13 |  |
|  |  | FOXJ1 |  |
|  |  | EPHX4 |  |
|  |  | GBP1 |  |
|  |  | SDC1 |  |
|  |  | LTBP2 |  |
|  |  | LGALS4 |  |
|  |  | C5AR1 |  |
|  |  | PODXL2 |  |
|  |  | ANTXR1 |  |
|  |  | DCDC2 |  |
|  |  | SMPDL3B |  |
|  |  | NPSR1 |  |
|  |  | CCDC198 |  |
|  |  | TNS4 |  |
|  |  | CLDN9 |  |
|  |  | H2AW |  |
|  |  | GJB2 |  |
|  |  | IGFL2 |  |
|  |  | PTPRH |  |
|  |  | JPH1 |  |
|  |  | C4A |  |
|  |  | CYP2B6 |  |
|  |  | LCP1 |  |
|  |  | APOL1 |  |
|  |  | CGREF1 |  |
|  |  | OTULINL |  |
|  |  | BICC1 |  |
|  |  | AGR2 |  |
|  |  | CEACAM1 |  |
|  |  | FAM166C |  |
|  |  | ALPP |  |
|  |  | DSP |  |
|  |  | ALPG |  |
|  |  | GOLT1A |  |
|  |  | AQP9 |  |
|  |  | COL15A1 |  |
|  |  | SCARF2 |  |
|  |  | NRCAM |  |
|  |  | RETREG1 |  |
|  |  | KRT23 |  |
|  |  | SAMD9L |  |
|  |  | HOXC13 |  |
|  |  | PADI2 |  |
|  |  | OLFM2 |  |
|  |  | FOSL1 |  |
|  |  | ATP2C2 |  |
|  |  | CYP2W1 |  |
|  |  | TGM2 |  |
|  |  | SGPP2 |  |
|  |  | CRISPLD1 |  |
|  |  | PIWIL1 |  |
|  |  | RAC3 |  |
|  |  | ERVMER34-1 |  |
|  |  | CFTR |  |
|  |  | MAGEA3 |  |
|  |  | EMX1 |  |
|  |  | SHISA2 |  |
|  |  | ECEL1 |  |
|  |  | GZMB |  |
|  |  | SBK1 |  |
|  |  | EVPL |  |
|  |  | CHST4 |  |
|  |  | SP6 |  |
|  |  | HAVCR1 |  |
|  |  | PRSS21 |  |
|  |  | AMIGO2 |  |
|  |  | SAMD5 |  |
|  |  | ERBB2 |  |
|  |  | FOXA1 |  |
|  |  | YBX2 |  |
|  |  | NMU |  |
|  |  | CEACAM6 |  |
|  |  | NUP210 |  |
|  |  | PPP1R1B |  |
|  |  | TNFRSF11A |  |
|  |  | ITGB6 |  |
|  |  | IGFBP1 |  |
|  |  | TNFAIP6 |  |
|  |  | LTB |  |
|  |  | CHIT1 |  |
|  |  | CNTNAP2 |  |
|  |  | CALHM6 |  |
|  |  | SELL |  |
|  |  | FAM83E |  |
|  |  | UNC5CL |  |
|  |  | PDZK1IP1 |  |
|  |  | MAGEA6 |  |
|  |  | CSAG1 |  |
|  |  | PRSS33 |  |
|  |  | C9orf152 |  |
|  |  | GDA |  |
|  |  | CSAG3 |  |
|  |  | TNNT1 |  |
|  |  | CXCL5 |  |
|  |  | MUC1 |  |
|  |  | ATP10B |  |
|  |  | THBS1 |  |
|  |  | IL22RA1 |  |
|  |  | HTR1D |  |
|  |  | ANXA13 |  |
|  |  | C11orf53 |  |
|  |  | POSTN |  |
|  |  | SAA2 |  |
|  |  | FIBCD1 |  |
|  |  | SLC19A3 |  |
|  |  | MAGEA12 |  |
|  |  | CXCL2 |  |
|  |  | PROM1 |  |
|  |  | TMEM150B |  |
|  |  | WNT7B |  |
|  |  | AMN |  |
|  |  | LGALS2 |  |
|  |  | DDC |  |
|  |  | AIM2 |  |
|  |  | MUC12 |  |
|  |  | MYO7B |  |
|  |  | SLC39A5 |  |
|  |  | MUC5B |  |
|  |  | TRIM29 |  |
|  |  | PRSS3 |  |
|  |  | IGF2BP1 |  |
|  |  | CXCL13 |  |
|  |  | LYZ |  |
|  |  | MLPH |  |
|  |  | LEFTY1 |  |
|  |  | AGR3 |  |
|  |  | HOXC12 |  |
|  |  | ACTL8 |  |
|  |  | PROX1 |  |
|  |  | DHRS2 |  |
|  |  | SPINK1 |  |
|  |  | UPK3A |  |
|  |  | GALNT5 |  |
|  |  | MUC20 |  |
|  |  | FOXQ1 |  |
|  |  | DKK1 |  |
|  |  | TESC |  |
|  |  | CAPN8 |  |
|  |  | FOXD1 |  |
|  |  | KLK10 |  |
|  |  | ONECUT3 |  |
|  |  | SYT8 |  |
|  |  | GUCY2C |  |
|  |  | NKX6-3 |  |
|  |  | KLK1 |  |
|  |  | CD177 |  |
|  |  | VGLL1 |  |
|  |  | OLFM4 |  |
|  |  | CLDN6 |  |
|  |  | CEACAM5 |  |
|  |  | GCNT3 |  |
|  |  | IHH |  |
|  |  | TM4SF5 |  |
|  |  | CEACAM7 |  |
|  |  | REG4 |  |
|  |  | CDX1 |  |
|  |  | RBP4 |  |
|  |  | VNN1 |  |
|  |  | MUC17 |  |
|  |  | GREM1 |  |
|  |  | APOA2 |  |
|  |  | GABRP |  |
|  |  | MAGEA4 |  |
|  |  | LCN2 |  |
|  |  | HABP2 |  |
|  |  | NOTUM |  |
|  |  | SAA1 |  |
|  |  | TACSTD2 |  |
|  |  | DMBT1 |  |
|  |  | TFF3 |  |
|  |  | MYOC |  |
|  |  | VEGFD |  |
|  |  | CLEC3B |  |
|  |  | MAL |  |
|  |  | SCNN1B |  |
|  |  | CCN5 |  |
|  |  | AQP4 |  |
|  |  | ADH7 |  |
|  |  | DPT |  |
|  |  | FAM180B |  |
|  |  | SCARA5 |  |
|  |  | CWH43 |  |
|  |  | PEBP4 |  |
|  |  | RXRG |  |
|  |  | PLP1 |  |
|  |  | ESRRG |  |
|  |  | C16orf89 |  |
|  |  | CKMT2 |  |
|  |  | CFD |  |
|  |  | CADM3 |  |
|  |  | GPX3 |  |
|  |  | P2RX2 |  |
|  |  | PI16 |  |
|  |  | ATP4A |  |
|  |  | LYVE1 |  |
|  |  | RDH12 |  |
|  |  | FXYD1 |  |
|  |  | MAMDC2 |  |
|  |  | LGI3 |  |
|  |  | GNG7 |  |
|  |  | ATP4B |  |
|  |  | PRRT4 |  |
|  |  | GSTM5 |  |
|  |  | VIT |  |
|  |  | SCNN1G |  |
|  |  | ASPA |  |
|  |  | ADRB2 |  |
|  |  | SH3GL2 |  |
|  |  | CIDEA |  |
|  |  | AQP10 |  |
|  |  | GIP |  |
|  |  | DAB1 |  |
|  |  | TMEM100 |  |
|  |  | OTOP3 |  |
|  |  | ADCYAP1R1 |  |
|  |  | RPRM |  |
|  |  | KRT24 |  |
|  |  | GFRA2 |  |
|  |  | SLC6A4 |  |
|  |  | PGA5 |  |
|  |  | MT1M |  |
|  |  | SOSTDC1 |  |
|  |  | PCSK2 |  |
|  |  | ECRG4 |  |
|  |  | MS4A10 |  |
|  |  | ENPP7 |  |
|  |  | KCNA5 |  |
|  |  | SLC5A7 |  |
|  |  | PPP1R1A |  |
|  |  | GCNT4 |  |
|  |  | NKX6-2 |  |
|  |  | CCKBR |  |
|  |  | C1QL1 |  |
|  |  | NPPC |  |
|  |  | PRIMA1 |  |
|  |  | DPP6 |  |
|  |  | ADHFE1 |  |
|  |  | AKR1C2 |  |
|  |  | FAM107A |  |
|  |  | EMILIN3 |  |
|  |  | ADH1B |  |
|  |  | MYOT |  |
|  |  | ATP1A2 |  |
|  |  | HPSE2 |  |
|  |  | NCAM1 |  |
|  |  | COL4A6 |  |
|  |  | CHODL |  |
|  |  | SFRP1 |  |
|  |  | ENPP3 |  |
|  |  | GKN2 |  |
|  |  | ZBTB16 |  |
|  |  | CMA1 |  |
|  |  | MYZAP |  |
|  |  | ASAH2 |  |
|  |  | CITED2 |  |
|  |  | SLC28A1 |  |
|  |  | RSPO2 |  |
|  |  | HBA1 |  |
|  |  | TMEM35A |  |
|  |  | TAFA4 |  |
|  |  | AKR1C1 |  |
|  |  | NEFL |  |
|  |  | TMEM132C |  |
|  |  | PYGM |  |
|  |  | MPZ |  |
|  |  | FLG |  |
|  |  | GKN1 |  |
|  |  | AKR1B15 |  |
|  |  | GPM6B |  |
|  |  | PGA3 |  |
|  |  | HPGD |  |
|  |  | CBR1 |  |
|  |  | S100B |  |
|  |  | TRIM50 |  |
|  |  | PDZRN4 |  |
|  |  | ANKRD35 |  |
|  |  | PCOLCE2 |  |
|  |  | SPINK2 |  |
|  |  | HLF |  |
|  |  | GPM6A |  |
|  |  | IGSF11 |  |
|  |  | FHL1 |  |
|  |  | GSN |  |
|  |  | CBLIF |  |
|  |  | IGFBP6 |  |
|  |  | CRYAB |  |
|  |  | HSPB8 |  |
|  |  | CASQ2 |  |
|  |  | UBE2QL1 |  |
|  |  | GRIK3 |  |
|  |  | KRT1 |  |
|  |  | HBB |  |
|  |  | USP2 |  |
|  |  | HSPB3 |  |
|  |  | P2RY14 |  |
|  |  | PGM5 |  |
|  |  | CALY |  |
|  |  | ZG16 |  |
|  |  | GUCA2B |  |
|  |  | KLF15 |  |
|  |  | SCN7A |  |
|  |  | DNASE1L3 |  |
|  |  | SLC25A4 |  |
|  |  | FABP4 |  |
|  |  | PTN |  |
|  |  | SST |  |
|  |  | SGCA |  |
|  |  | ADAMTSL1 |  |
|  |  | LGALS9C |  |
|  |  | GSTM2 |  |
|  |  | AMPD1 |  |
|  |  | KCNB1 |  |
|  |  | SPINK7 |  |
|  |  | SLC5A5 |  |
|  |  | CDH19 |  |
|  |  | CPA2 |  |
|  |  | PNCK |  |
|  |  | CKB |  |
|  |  | MMRN1 |  |
|  |  | SLC2A4 |  |
|  |  | ANGPTL1 |  |
|  |  | LGI4 |  |
|  |  | PPP1R3C |  |
|  |  | TTC36 |  |
|  |  | ARC |  |
|  |  | NAP1L2 |  |
|  |  | SLC18A3 |  |
|  |  | GHR |  |
|  |  | GFRA1 |  |
|  |  | LGALS9B |  |
|  |  | PLIN4 |  |
|  |  | CCDC69 |  |
|  |  | APOBEC2 |  |
|  |  | PTCHD1 |  |
|  |  | PDK4 |  |
|  |  | ENDOU |  |
|  |  | PTGS1 |  |
|  |  | SMIM5 |  |
|  |  | INSYN1 |  |
|  |  | ANGPTL7 |  |
|  |  | PLAC9 |  |
|  |  | CHGA |  |
|  |  | GPER1 |  |
|  |  | MT1A |  |
|  |  | MT1X |  |
|  |  | MEP1B |  |
|  |  | TMEM252 |  |
|  |  | CYP4B1 |  |
|  |  | CAB39L |  |
|  |  | PLCXD3 |  |
|  |  | KCNE2 |  |
|  |  | FNDC5 |  |
|  |  | BTG2 |  |
|  |  | PCP4L1 |  |
|  |  | CTSG |  |
|  |  | INA |  |
|  |  | LDB3 |  |
|  |  | CNTFR |  |
|  |  | TREH |  |
|  |  | APOA4 |  |
|  |  | KLF4 |  |
|  |  | TMPRSS15 |  |
|  |  | CHIA |  |
|  |  | AARD |  |
|  |  | SCUBE2 |  |
|  |  | BCHE |  |
|  |  | RBPMS2 |  |
|  |  | SCUBE1 |  |
|  |  | METTL7A |  |
|  |  | CARTPT |  |
|  |  | TCEAL2 |  |
|  |  | PDZD4 |  |
|  |  | RERGL |  |
|  |  | PLIN1 |  |
|  |  | PSAPL1 |  |
|  |  | ABCA8 |  |
|  |  | LMOD1 |  |
|  |  | SLC2A2 |  |
|  |  | CTNNA3 |  |
|  |  | MYH11 |  |
|  |  | PHYHD1 |  |
|  |  | APOC3 |  |
|  |  | TMOD1 |  |
|  |  | PTGR1 |  |
|  |  | DEFA5 |  |
|  |  | FCER1A |  |
|  |  | DUSP26 |  |
|  |  | HBA2 |  |
|  |  | SOX15 |  |
|  |  | ATOH8 |  |
|  |  | TNXB |  |
|  |  | FAXDC2 |  |
|  |  | CHRDL1 |  |
|  |  | NTN1 |  |
|  |  | SH3BGR |  |
|  |  | ACTN2 |  |
|  |  | S100G |  |
|  |  | KCNMB1 |  |
|  |  | CD36 |  |
|  |  | ASB5 |  |
|  |  | PTGDR2 |  |
|  |  | NIPAL4 |  |
|  |  | MAPK4 |  |
|  |  | ADIPOQ |  |
|  |  | WSCD2 |  |
|  |  | NEGR1 |  |
|  |  | ACKR1 |  |
|  |  | PLN |  |
|  |  | AFF3 |  |
|  |  | EFS |  |
|  |  | NPY |  |
|  |  | RGS2 |  |
|  |  | VIP |  |
|  |  | HSPB6 |  |
|  |  | CNN1 |  |
|  |  | MYRIP |  |
|  |  | GHRL |  |
|  |  | BEND5 |  |
|  |  | NPTX1 |  |
|  |  | GFRA3 |  |
|  |  | ASB2 |  |
|  |  | SRPX |  |
|  |  | CIDEC |  |
|  |  | NAALADL1 |  |
|  |  | SOX21 |  |
|  |  | DES |  |
|  |  | SLC22A17 |  |
|  |  | MYL9 |  |
|  |  | DIRAS1 |  |
|  |  | SNRPN |  |
|  |  | ARL4D |  |
|  |  | METTL24 |  |
|  |  | KCNK3 |  |
|  |  | TNFAIP8L3 |  |
|  |  | PNOC |  |
|  |  | CCKAR |  |
|  |  | KCNJ16 |  |
|  |  | ID4 |  |
|  |  | PER1 |  |
|  |  | JAM2 |  |
|  |  | PENK |  |
|  |  | ACTG2 |  |
|  |  | FRRS1L |  |
|  |  | DUSP1 |  |
|  |  | COX7A1 |  |
|  |  | DNER |  |
|  |  | HSPA2 |  |
|  |  | TSC22D3 |  |
|  |  | KIT |  |
|  |  | MORN5 |  |
|  |  | PSD |  |
|  |  | SYNM |  |
|  |  | BHMT2 |  |
|  |  | GCG |  |
|  |  | SULT2A1 |  |
|  |  | ABI3BP |  |
|  |  | MTTP |  |
|  |  | GPR155 |  |
|  |  | C8orf88 |  |
|  |  | PDE2A |  |
|  |  | PPP1R14A |  |
|  |  | RCAN2 |  |
|  |  | SLC7A8 |  |
|  |  | MLNR |  |
|  |  | C7 |  |
|  |  | ACOX2 |  |
|  |  | TMEM25 |  |
|  |  | DNAJB5 |  |
|  |  | REP15 |  |
|  |  | CLDN5 |  |
|  |  | TPSG1 |  |
|  |  | ENTPD3 |  |
|  |  | NECAB1 |  |
|  |  | MAOB |  |
|  |  | CYP3A4 |  |
|  |  | MFAP4 |  |
|  |  | CHAD |  |
|  |  | CNTN1 |  |
|  |  | ALDH3A1 |  |
|  |  | GPAT3 |  |
|  |  | LIMS2 |  |
|  |  | SNCG |  |
|  |  | SMYD1 |  |
|  |  | SFRP5 |  |
|  |  | LIPF |  |
|  |  | FXYD6 |  |
|  |  | C1QTNF7 |  |
|  |  | COL4A5 |  |
|  |  | SYNGR1 |  |
|  |  | RBP2 |  |
|  |  | PSCA |  |
|  |  | CHRM2 |  |
|  |  | TMPRSS11B |  |
|  |  | CPE |  |
|  |  | ARHGEF26 |  |
|  |  | MYOCD |  |
|  |  | B3GNT6 |  |
|  |  | EPHA7 |  |
|  |  | CGNL1 |  |
|  |  | SYNPO2 |  |
|  |  | LRRN4CL |  |
|  |  | PRKAR2B |  |
|  |  | MAP6 |  |
|  |  | SLC2A12 |  |
|  |  | SLC9A4 |  |
|  |  | GYPC |  |
|  |  | SCGB2A1 |  |
|  |  | FBP2 |  |
|  |  | TENT5B |  |
|  |  | ADAMTS1 |  |
|  |  | SMPX |  |
|  |  | APOB |  |
|  |  | ANKRD65 |  |
|  |  | HOXA4 |  |
|  |  | LDHD |  |
|  |  | PITPNM3 |  |
|  |  | BEX4 |  |
|  |  | MGAM |  |
|  |  | KRT78 |  |
|  |  | MUC21 |  |
|  |  | MAOA |  |
|  |  | CCL21 |  |
|  |  | CSRP1 |  |
|  |  | AGTR1 |  |
|  |  | DACT3 |  |
|  |  | NSG1 |  |
|  |  | HSPB7 |  |
|  |  | CYBRD1 |  |
|  |  | JPH2 |  |
|  |  | DEFA6 |  |
|  |  | S100A12 |  |
|  |  | NIBAN1 |  |
|  |  | ATP1B2 |  |
|  |  | IGHV3OR16-17 |  |
|  |  | SORBS2 |  |
|  |  | CEACAM20 |  |
|  |  | WIF1 |  |
|  |  | CLCA1 |  |
|  |  | KCNJ15 |  |
|  |  | GAMT |  |
|  |  | FOXP2 |  |
|  |  | SORBS1 |  |
|  |  | RNASE1 |  |
|  |  | ITGA8 |  |
|  |  | CSTA |  |
|  |  | NNAT |  |
|  |  | GREM2 |  |
|  |  | FGL2 |  |
|  |  | LYNX1 |  |
|  |  | REEP1 |  |
|  |  | CAVIN2 |  |
|  |  | ARHGDIG |  |
|  |  | AKR1B10 |  |
|  |  | TACR2 |  |
|  |  | RGMA |  |
|  |  | GNAO1 |  |
|  |  | ADCY5 |  |
|  |  | F10 |  |
|  |  | SBSPON |  |
|  |  | TAGLN |  |
|  |  | NDN |  |
|  |  | FOXF2 |  |
|  |  | C6orf58 |  |
|  |  | ITLN2 |  |
|  |  | PDZRN3 |  |
|  |  | SERPINB13 |  |
|  |  | ZBTB7C |  |
|  |  | ADAM33 |  |
|  |  | ARHGEF25 |  |
|  |  | EEF1A2 |  |
|  |  | CXCL12 |  |
|  |  | AOC3 |  |
|  |  | C3orf85 |  |
|  |  | STMN2 |  |
|  |  | SPEG |  |
|  |  | PRKAA2 |  |
|  |  | FEV |  |
|  |  | NXPH3 |  |
|  |  | LDOC1 |  |
|  |  | DMD |  |
|  |  | CLCA4 |  |
|  |  | REEP2 |  |
|  |  | SELENOP |  |
|  |  | RNF150 |  |
|  |  | KCNMA1 |  |
|  |  | C14orf132 |  |
|  |  | DAAM2 |  |
|  |  | SUSD4 |  |
|  |  | CES1 |  |
|  |  | TSPAN7 |  |
|  |  | DKK4 |  |
|  |  | XPNPEP2 |  |
|  |  | DCN |  |
|  |  | F13A1 |  |
|  |  | SLC51B |  |
|  |  | FOXF1 |  |
|  |  | BMP3 |  |
|  |  | MT1E |  |
|  |  | FAM189A2 |  |
|  |  | FOSB |  |
|  |  | GSTA2 |  |
|  |  | C17orf78 |  |
|  |  | RGN |  |
|  |  | PCP4 |  |
|  |  | SHISA3 |  |
|  |  | MLN |  |
|  |  | TPM2 |  |
|  |  | CLU |  |
|  |  | APOA1 |  |
|  |  | SLURP1 |  |
|  |  | OGN |  |
|  |  | TPSD1 |  |
|  |  | JCHAIN |  |
|  |  | CHGB |  |
|  |  | HAND2 |  |
|  |  | PRUNE2 |  |
|  |  | IRX3 |  |
|  |  | MYLK |  |
|  |  | TPSAB1 |  |
|  |  | IVL |  |
|  |  | SCG2 |  |
|  |  | FBLN1 |  |
|  |  | MRGPRF |  |
|  |  | TNFRSF17 |  |
|  |  | HIF3A |  |
|  |  | GBP6 |  |
|  |  | C11orf86 |  |
|  |  | TMPRSS11E |  |
|  |  | SBSN |  |
|  |  | CRCT1 |  |
|  |  | CXCL17 |  |
|  |  | PDLIM3 |  |
|  |  | MFAP5 |  |
|  |  | GSTA1 |  |
|  |  | PRELP |  |
|  |  | CPED1 |  |
|  |  | GPT |  |
|  |  | PTGDS |  |
|  |  | EDN3 |  |
|  |  | S100A8 |  |
|  |  | FLNC |  |
|  |  | SMOC2 |  |
|  |  | ACTA2 |  |
|  |  | TGM3 |  |
|  |  | NCCRP1 |  |
|  |  | BEX2 |  |
|  |  | SLC28A2 |  |
|  |  | KRT4 |  |
|  |  | SCGN |  |
|  |  | FABP2 |  |
|  |  | REG3A |  |
|  |  | CAPN9 |  |
|  |  | KRT6C |  |
|  |  | A2ML1 |  |
|  |  | BARX1 |  |
|  |  | GATA5 |  |
|  |  | FAM3B |  |
|  |  | KRT20 |  |
|  |  | ADH4 |  |
|  |  | CYP2C18 |  |
|  |  | ALDOB |  |
|  |  | UGT2B17 |  |
|  |  | TFF2 |  |
|  |  | MUCL3 |  |
|  |  | MT1G |  |
|  |  | APOBEC1 |  |
|  |  | OTC |  |
|  |  | VSIG2 |  |
|  |  | LTF |  |
|  |  | AADAC |  |
|  |  | PGC |  |
|  |  | BEX1 |  |
|  |  | ADH1C |  |
|  |  | KRT13 |  |
|  |  | SOX2 |  |
|  |  | PCSK1N |  |
|  |  | GSTM1 |  |
|  |  | FCGBP |  |
|  |  | KRT5 |  |
|  |  | TFF1 |  |
|  |  | PHGR1 |  |
