# Supplementary material for: Identification of molecular patterns and prognostic models of epithelial–mesenchymal transition- and immune-combined index in the gastric cancer
Source: Front Pharmacol. 2022 Aug 9;13:958070. doi: 10.3389/fphar.2022.958070 (PMC9397546; doi:10.3389/fphar.2022.958070)
Supplement: Supplementary file 3 [file DataSheet1.PDF]

Identification of Molecular Patterns of Epithelial Mesenchymal Transition- and Immune-Combined Index in the Gastric Cancer

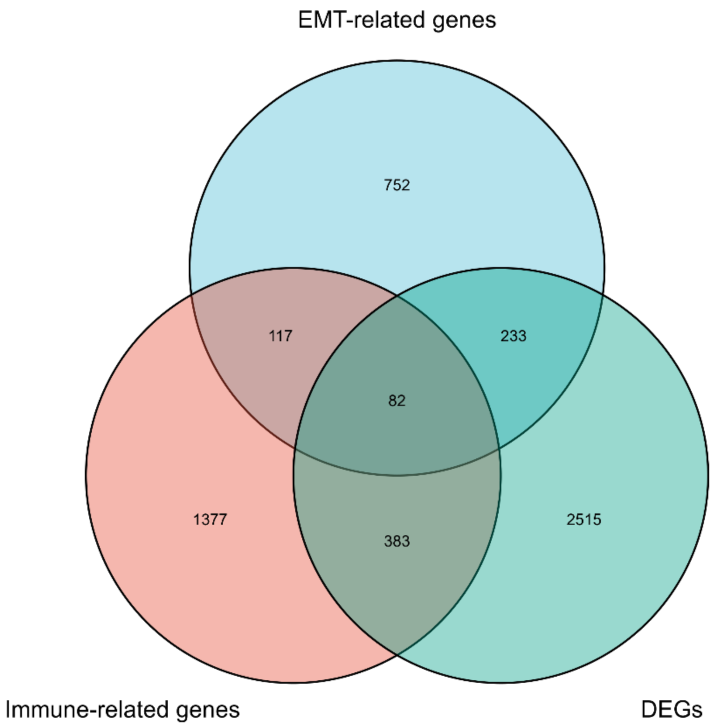

Figure S1: Overlapping Venn diagrams of EMT-related genes and immune-related genes.

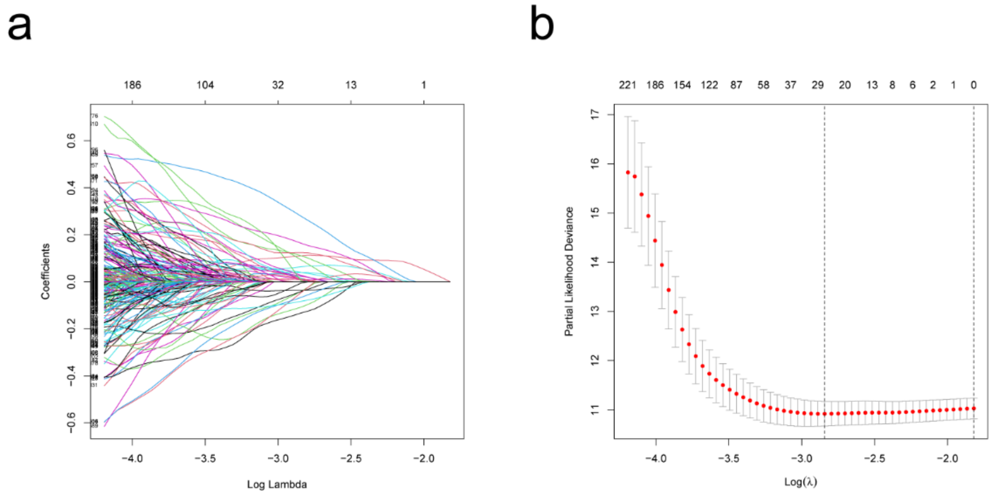

Figure S2: Construction of risk score model. (a) LASSO coefficient profiles. (b) Selection of the tuning parameter (lambda) in the LASSO model by 10-fold cross-validation based on minimum criteria for OS.

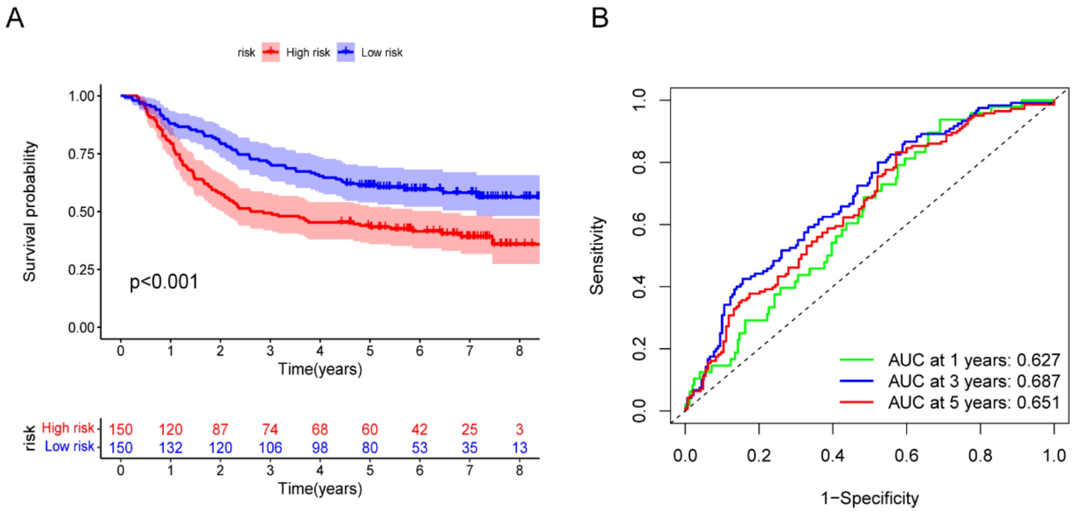

Figure S3: External validation of EIRG_score (GSE62254). (a) KM survival analysis of EIRG_score. (b) ROC curve analysis of EIRG_score.

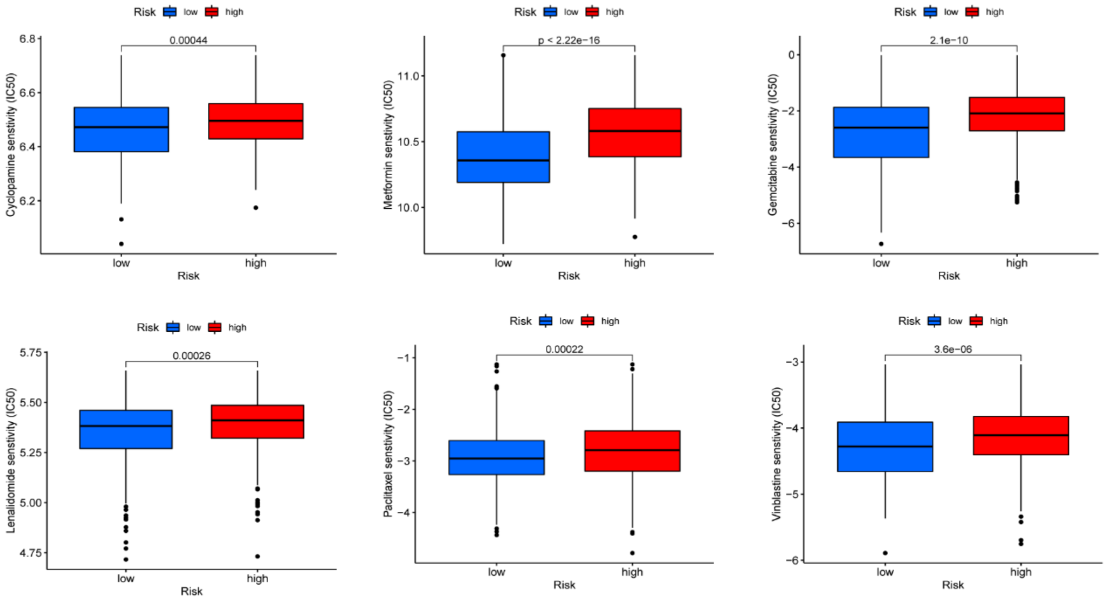

Figure S4: Drug sensitivity analysis of EIRG_score.

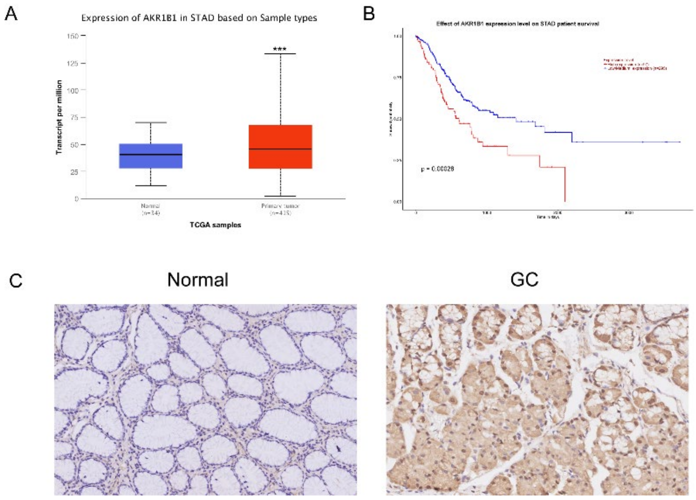

Figure S5: Expression and prognosis of AKR1B1 in STAD.
