# Supplementary material for: Identification of molecular patterns and prognostic models of epithelial–mesenchymal transition- and immune-combined index in the gastric cancer
Source: Front Pharmacol. 2022 Aug 9;13:958070. doi: 10.3389/fphar.2022.958070 (PMC9397546; doi:10.3389/fphar.2022.958070)
Supplement: Supplementary file 4 [file Table3.DOCX]

**Table S3: Quantitative results of EIRG_score of 804 gastric cancer patients in the combined cohort.**

| id | futime | fustat | EIRG_Score | Group |
| --- | --- | --- | --- | --- |
| TCGA-BR-8588 | 1.065753425 | 0 | 1.841530467 | high |
| TCGA-CD-A4MG | 0.547945205 | 1 | 0.338551423 | low |
| TCGA-HU-8602 | 1.860273973 | 0 | 0.454375232 | low |
| TCGA-HU-A4GY | 0.021917808 | 0 | 0.488268057 | low |
| TCGA-BR-8365 | 1.460273973 | 1 | 8.157685337 | high |
| TCGA-D7-5578 | 1.054794521 | 0 | 1.979212735 | high |
| TCGA-CG-4465 | 0.750684932 | 1 | 2.05965858 | high |
| TCGA-CG-5720 | 0.082191781 | 1 | 0.750299573 | low |
| TCGA-CD-A486 | 0.526027397 | 1 | 1.020437919 | high |
| TCGA-CG-5716 | 0.003 | 0 | 0.159315905 | low |
| TCGA-VQ-A91K | 5.101369863 | 0 | 0.155348703 | low |
| TCGA-IN-A6RL | 1.112328767 | 1 | 2.749106816 | high |
| TCGA-R5-A7ZF | 0.709589041 | 1 | 1.025144494 | high |
| TCGA-CG-4436 | 0.665753425 | 0 | 0.252909517 | low |
| TCGA-BR-8676 | 0.62739726 | 0 | 0.738437519 | low |
| TCGA-D7-A747 | 0.698630137 | 1 | 1.694374801 | high |
| TCGA-BR-A44U | 1.156164384 | 1 | 2.37531675 | high |
| TCGA-KB-A93G | 1.679452055 | 0 | 1.074678486 | high |
| TCGA-BR-8367 | 2.194520548 | 1 | 5.223149586 | high |
| TCGA-D7-6521 | 1.545205479 | 0 | 4.000052053 | high |
| TCGA-CG-4466 | 1.580821918 | 0 | 0.217259207 | low |
| TCGA-CD-8524 | 1.063013699 | 0 | 1.191170995 | high |
| TCGA-BR-7723 | 2.394520548 | 1 | 1.968860464 | high |
| TCGA-BR-6801 | 3.350684932 | 0 | 1.529802657 | high |
| TCGA-CG-4305 | 1.328767123 | 0 | 4.075046082 | high |
| TCGA-HU-A4GX | 1.687671233 | 0 | 0.178310273 | low |
| TCGA-BR-8382 | 2.087671233 | 1 | 0.383757234 | low |
| TCGA-VQ-A91D | 0.975342466 | 1 | 0.566878857 | low |
| TCGA-KB-A6F7 | 5.301369863 | 0 | 0.588490454 | low |
| TCGA-VQ-A91V | 3.553424658 | 0 | 0.351560381 | low |
| TCGA-CD-8529 | 1.024657534 | 0 | 2.287506274 | high |
| TCGA-CG-4460 | 1.832876712 | 1 | 3.043843975 | high |
| TCGA-BR-A4J9 | 0.038356164 | 0 | 1.536593798 | high |
| TCGA-D7-A6EZ | 1.693150685 | 1 | 0.606109683 | low |
| TCGA-CD-8527 | 0.597260274 | 1 | 2.022164218 | high |
| TCGA-BR-6452 | 2.890410959 | 0 | 0.594110517 | low |
| TCGA-D7-8573 | 1.624657534 | 0 | 1.82759811 | high |
| TCGA-D7-A6F0 | 1.857534247 | 0 | 0.606060544 | low |
| TCGA-FP-A8CX | 0.019178082 | 0 | 0.935759762 | low |
| TCGA-D7-8570 | 2.060273973 | 0 | 0.457560177 | low |
| TCGA-KB-A93H | 3.136986301 | 0 | 0.092927008 | low |
| TCGA-CG-4476 | 0.003 | 0 | 2.94662003 | high |
| TCGA-HU-A4GF | 2.150684932 | 0 | 0.076594747 | low |
| TCGA-HU-A4GT | 0.542465753 | 0 | 0.266889891 | low |
| TCGA-VQ-A91A | 3.287671233 | 0 | 1.088158892 | high |
| TCGA-VQ-A8PX | 5.380821918 | 0 | 0.09321287 | low |
| TCGA-FP-A4BF | 0.460273973 | 1 | 3.087568247 | high |
| TCGA-BR-8380 | 0.057534247 | 0 | 7.084055789 | high |
| TCGA-VQ-A91Q | 1.734246575 | 1 | 0.698047021 | low |
| TCGA-FP-7829 | 1.62739726 | 0 | 0.184857208 | low |
| TCGA-HF-7134 | 4.350684932 | 0 | 0.397417496 | low |
| TCGA-CG-4441 | 1.167123288 | 1 | 4.476787241 | high |
| TCGA-VQ-AA6J | 2.295890411 | 0 | 1.0000835 | low |
| TCGA-VQ-A8PQ | 1.304109589 | 1 | 1.275245123 | high |
| TCGA-B7-A5TN | 0.78630137 | 0 | 4.149785786 | high |
| TCGA-VQ-A8E0 | 1.539726027 | 1 | 1.686887938 | high |
| TCGA-BR-8284 | 0.671232877 | 1 | 3.157191636 | high |
| TCGA-VQ-A8PB | 2.857534247 | 1 | 2.152327704 | high |
| TCGA-HU-A4GJ | 1.780821918 | 0 | 1.174775547 | high |
| TCGA-BR-A4QL | 1.345205479 | 1 | 1.133726995 | high |
| TCGA-BR-7704 | 2.936986301 | 0 | 0.145578402 | low |
| TCGA-CD-A4MH | 1.016438356 | 0 | 0.091030265 | low |
| TCGA-VQ-A91Y | 0.810958904 | 1 | 3.766338791 | high |
| TCGA-RD-A8N9 | 2.967123288 | 0 | 1.335738938 | high |
| TCGA-HU-A4G2 | 2.024657534 | 0 | 0.374579941 | low |
| TCGA-B7-A5TK | 0.789041096 | 0 | 0.487817498 | low |
| TCGA-SW-A7EB | 0.482191781 | 0 | 0.528841643 | low |
| TCGA-BR-8060 | 0.953424658 | 1 | 4.457109766 | high |
| TCGA-BR-8484 | 2.098630137 | 1 | 1.851084139 | high |
| TCGA-BR-8059 | 1.202739726 | 1 | 9.487268452 | high |
| TCGA-F1-6177 | 0.003 | 0 | 0.265115903 | low |
| TCGA-BR-6710 | 0.747945205 | 0 | 1.175193269 | high |
| TCGA-KB-A93J | 3.079452055 | 0 | 0.177622802 | low |
| TCGA-BR-8080 | 0.8 | 1 | 8.554781028 | high |
| TCGA-IN-8462 | 1.567123288 | 0 | 1.547703341 | high |
| TCGA-CD-A487 | 1.024657534 | 0 | 1.115452796 | high |
| TCGA-BR-4369 | 0.003 | 0 | 1.287921387 | high |
| TCGA-D7-6519 | 1.712328767 | 0 | 4.841653779 | high |
| TCGA-BR-8372 | 2.605479452 | 0 | 0.350633376 | low |
| TCGA-FP-7916 | 1.17260274 | 1 | 1.337602487 | high |
| TCGA-VQ-AA6D | 1.42739726 | 0 | 0.29159279 | low |
| TCGA-CG-5734 | 0.665753425 | 1 | 0.249227732 | low |
| TCGA-BR-8683 | 0.821917808 | 1 | 4.481684308 | high |
| TCGA-BR-A4J4 | 0.043835616 | 0 | 0.364845526 | low |
| TCGA-D7-6522 | 1.550684932 | 0 | 0.92053176 | low |
| TCGA-BR-6803 | 2.6 | 0 | 2.779094392 | high |
| TCGA-BR-8483 | 0.449315068 | 0 | 0.544960106 | low |
| TCGA-BR-6705 | 2.134246575 | 1 | 2.568126708 | high |
| TCGA-BR-8678 | 2.065753425 | 0 | 0.352527934 | low |
| TCGA-CG-4462 | 0.003 | 1 | 5.722075348 | high |
| TCGA-BR-8295 | 0.183561644 | 1 | 2.080720927 | high |
| TCGA-D7-8578 | 1.761643836 | 0 | 1.542185986 | high |
| TCGA-R5-A7ZI | 6.210958904 | 0 | 0.093012127 | low |
| TCGA-HU-8610 | 0.063013699 | 0 | 0.605652922 | low |
| TCGA-VQ-AA69 | 2.367123288 | 0 | 0.072306249 | low |
| TCGA-CG-5723 | 6.838356164 | 0 | 0.814131033 | low |
| TCGA-RD-A8N2 | 9.698630137 | 0 | 1.286694144 | high |
| TCGA-D7-8576 | 1.221917808 | 1 | 3.545025989 | high |
| TCGA-BR-8058 | 3.104109589 | 0 | 2.552399381 | high |
| TCGA-VQ-A91E | 1.819178082 | 0 | 0.34059731 | low |
| TCGA-VQ-A8PO | 0.77260274 | 1 | 0.411375523 | low |
| TCGA-CG-4444 | 3.920547945 | 0 | 1.716322822 | high |
| TCGA-BR-7851 | 1.035616438 | 0 | 0.715355484 | low |
| TCGA-BR-8591 | 2.345205479 | 0 | 1.785144225 | high |
| TCGA-D7-A6EV | 0.936986301 | 0 | 1.06860239 | high |
| TCGA-R5-A805 | 0.769863014 | 1 | 0.498150558 | low |
| TCGA-MX-A5UG | 0.309589041 | 1 | 7.295532511 | high |
| TCGA-VQ-A94U | 2.243835616 | 0 | 1.377710123 | high |
| TCGA-BR-8590 | 0.778082192 | 1 | 12.1049543 | high |
| TCGA-BR-6565 | 0.764383562 | 1 | 2.31976787 | high |
| TCGA-VQ-AA6K | 1.035616438 | 1 | 1.705411633 | high |
| TCGA-F1-A448 | 1.77260274 | 0 | 0.839683558 | low |
| TCGA-CD-8535 | 1.068493151 | 0 | 0.1155944 | low |
| TCGA-D7-5577 | 2.142465753 | 1 | 1.566728383 | high |
| TCGA-CG-5722 | 0.082191781 | 0 | 0.926964768 | low |
| TCGA-CG-5726 | 2.41369863 | 1 | 0.824310426 | low |
| TCGA-BR-8373 | 1.232876712 | 0 | 0.62696946 | low |
| TCGA-HJ-7597 | 2.205479452 | 1 | 4.818036092 | high |
| TCGA-HU-A4GQ | 0.008219178 | 1 | 0.884477657 | low |
| TCGA-BR-4256 | 0.778082192 | 1 | 1.137641437 | high |
| TCGA-CG-4443 | 2.498630137 | 0 | 0.280533437 | low |
| TCGA-BR-A4PF | 0.095890411 | 0 | 0.148464432 | low |
| TCGA-VQ-A94R | 3.545205479 | 1 | 0.366494094 | low |
| TCGA-CG-5725 | 1.252054795 | 1 | 0.758977487 | low |
| TCGA-BR-6709 | 1.01369863 | 1 | 4.644690778 | high |
| TCGA-MX-A666 | 1.169863014 | 0 | 2.138518018 | high |
| TCGA-VQ-AA64 | 1.534246575 | 1 | 4.254490836 | high |
| TCGA-HF-7132 | 6.44109589 | 0 | 0.353174239 | low |
| TCGA-CG-5732 | 5.753424658 | 1 | 2.449753108 | high |
| TCGA-HU-A4G3 | 0.465753425 | 0 | 0.89650669 | low |
| TCGA-IN-AB1X | 1.126027397 | 0 | 0.680147368 | low |
| TCGA-CD-5804 | 0.003 | 0 | 1.391386463 | high |
| TCGA-CG-5721 | 0.501369863 | 0 | 0.06227634 | low |
| TCGA-BR-6566 | 2.731506849 | 0 | 0.783117803 | low |
| TCGA-HU-8244 | 2.032876712 | 0 | 0.131120619 | low |
| TCGA-FP-A9TM | 0.517808219 | 0 | 0.384039356 | low |
| TCGA-VQ-A8DT | 4.065753425 | 0 | 0.515291953 | low |
| TCGA-CD-8526 | 1.043835616 | 0 | 0.479816131 | low |
| TCGA-HU-A4H6 | 1.764383562 | 0 | 0.736493386 | low |
| TCGA-RD-A8N6 | 0.745205479 | 1 | 4.492644751 | high |
| TCGA-D7-A4YX | 3.035616438 | 0 | 0.906369484 | low |
| TCGA-BR-4191 | 1.528767123 | 1 | 5.277673265 | high |
| TCGA-B7-A5TI | 1.630136986 | 0 | 3.417523845 | high |
| TCGA-BR-4280 | 0.550684932 | 1 | 0.340899258 | low |
| TCGA-BR-8366 | 0.079452055 | 0 | 1.289594544 | high |
| TCGA-D7-6528 | 1.268493151 | 0 | 0.088067892 | low |
| TCGA-BR-6707 | 1.657534247 | 1 | 0.334763626 | low |
| TCGA-BR-8368 | 0.35890411 | 0 | 0.20632804 | low |
| TCGA-BR-4267 | 0.515068493 | 1 | 2.324652427 | high |
| TCGA-R5-A7O7 | 3.805479452 | 0 | 1.19405758 | high |
| TCGA-RD-A7BS | 0.920547945 | 1 | 5.116848942 | high |
| TCGA-VQ-AA6F | 4.509589041 | 0 | 0.429640456 | low |
| TCGA-D7-8575 | 1.517808219 | 1 | 2.04092785 | high |
| TCGA-BR-8077 | 0.057534247 | 0 | 0.959521216 | low |
| TCGA-CD-8531 | 1.049315068 | 0 | 0.71150028 | low |
| TCGA-BR-8485 | 0.767123288 | 0 | 2.473200739 | high |
| TCGA-HU-A4H5 | 1.983561644 | 0 | 0.669886046 | low |
| TCGA-D7-8579 | 1.742465753 | 0 | 1.484640133 | high |
| TCGA-BR-8677 | 2.22739726 | 0 | 1.045589035 | high |
| TCGA-HU-A4HB | 1.306849315 | 1 | 0.543072249 | low |
| TCGA-BR-7707 | 2.98630137 | 0 | 0.553433183 | low |
| TCGA-BR-8384 | 0.309589041 | 0 | 7.972646541 | high |
| TCGA-BR-8361 | 2.591780822 | 0 | 0.236741599 | low |
| TCGA-D7-6527 | 0.854794521 | 1 | 1.275241343 | high |
| TCGA-RD-A8MW | 3.15890411 | 1 | 2.489432482 | high |
| TCGA-CG-4475 | 1.915068493 | 0 | 3.86220645 | high |
| TCGA-CD-5801 | 1.098630137 | 1 | 0.417024744 | low |
| TCGA-VQ-A8PP | 1.950684932 | 1 | 1.069317094 | high |
| TCGA-HU-8604 | 1.901369863 | 0 | 0.486501584 | low |
| TCGA-BR-6456 | 1.44109589 | 1 | 3.957925696 | high |
| TCGA-BR-A4CR | 0.003 | 0 | 0.170981123 | low |
| TCGA-BR-4371 | 0.003 | 0 | 0.208646165 | low |
| TCGA-MX-A663 | 0.821917808 | 1 | 7.043986256 | high |
| TCGA-VQ-A8PF | 0.208219178 | 1 | 0.263988958 | low |
| TCGA-BR-4367 | 0.003 | 0 | 2.460475719 | high |
| TCGA-BR-8381 | 0.61369863 | 0 | 1.685896116 | high |
| TCGA-FP-7998 | 1.857534247 | 0 | 0.487521649 | low |
| TCGA-VQ-A8DZ | 1.084931507 | 1 | 8.439675672 | high |
| TCGA-CG-4306 | 0.002739726 | 1 | 0.314240978 | low |
| TCGA-HU-8238 | 0.126027397 | 0 | 0.312447995 | low |
| TCGA-BR-8081 | 2.687671233 | 0 | 0.548379066 | low |
| TCGA-VQ-AA6G | 2.169863014 | 1 | 0.28335911 | low |
| TCGA-VQ-A94O | 1.753424658 | 1 | 4.369819876 | high |
| TCGA-BR-7197 | 0.767123288 | 0 | 0.320118179 | low |
| TCGA-D7-A6EY | 0.953424658 | 1 | 1.309709993 | high |
| TCGA-D7-6815 | 1.331506849 | 0 | 1.633776946 | high |
| TCGA-CD-8525 | 1.049315068 | 0 | 0.760858099 | low |
| TCGA-CG-5719 | 0.084931507 | 0 | 4.782023974 | high |
| TCGA-RD-A8NB | 1.405479452 | 1 | 0.457924334 | low |
| TCGA-BR-A4IV | 2.380821918 | 1 | 1.036537577 | high |
| TCGA-HF-A5NB | 2.542465753 | 0 | 0.420443438 | low |
| TCGA-FP-8099 | 1.421917808 | 0 | 1.148927106 | high |
| TCGA-VQ-A94T | 0.936986301 | 1 | 2.522988021 | high |
| TCGA-CD-8533 | 1.282191781 | 0 | 0.757402828 | low |
| TCGA-CG-4469 | 0.589041096 | 1 | 1.669215952 | high |
| TCGA-VQ-A8PE | 1.849315068 | 1 | 1.153312915 | high |
| GSM2235563 | 1.150684932 | 1 | 5.49206109 | high |
| GSM2235566 | 0.904109589 | 1 | 0.796815337 | low |
| GSM2235567 | 3.534246575 | 1 | 2.432747296 | high |
| GSM2235569 | 6.739726027 | 0 | 0.417395651 | low |
| GSM2235573 | 6.739726027 | 0 | 0.654061294 | low |
| GSM2235574 | 0.904109589 | 1 | 4.107607932 | high |
| GSM2235575 | 7.068493151 | 0 | 0.875849562 | low |
| GSM2235576 | 0.328767123 | 1 | 0.451190427 | low |
| GSM2235577 | 1.561643836 | 1 | 4.078998135 | high |
| GSM2235579 | 1.397260274 | 1 | 7.928602775 | high |
| GSM2235582 | 1.068493151 | 1 | 7.788731809 | high |
| GSM2235586 | 6.328767123 | 0 | 0.177699329 | low |
| GSM2235587 | 5.260273973 | 1 | 2.220000995 | high |
| GSM2235593 | 6.328767123 | 0 | 0.235112092 | low |
| GSM2235595 | 1.232876712 | 1 | 0.696831174 | low |
| GSM2235596 | 6.164383562 | 0 | 0.862865115 | low |
| GSM2235597 | 6.904109589 | 0 | 0.290943291 | low |
| GSM2235598 | 1.726027397 | 1 | 4.68517061 | high |
| GSM2235599 | 6.575342466 | 0 | 0.582240736 | low |
| GSM2235600 | 6.082191781 | 0 | 0.249860646 | low |
| GSM2235601 | 6.082191781 | 0 | 0.718585311 | low |
| GSM2235602 | 6.739726027 | 0 | 0.494890104 | low |
| GSM2235604 | 6.082191781 | 0 | 0.607173019 | low |
| GSM2235607 | 0.493150685 | 1 | 2.274365984 | high |
| GSM2235610 | 1.890410959 | 1 | 1.686565134 | high |
| GSM2235611 | 0.657534247 | 1 | 0.683592472 | low |
| GSM2235617 | 5.835616438 | 0 | 0.217901223 | low |
| GSM2235619 | 5.753424658 | 0 | 0.997438371 | low |
| GSM2235620 | 5.835616438 | 0 | 0.617769538 | low |
| GSM2235621 | 2.712328767 | 1 | 1.114570288 | high |
| GSM2235623 | 1.726027397 | 1 | 0.951909027 | low |
| GSM2235625 | 5.753424658 | 0 | 0.39241795 | low |
| GSM2235630 | 5.753424658 | 0 | 0.455811139 | low |
| GSM2235632 | 6.246575342 | 0 | 0.255294924 | low |
| GSM2235633 | 1.397260274 | 1 | 0.846616558 | low |
| GSM2235634 | 6.082191781 | 0 | 1.185227373 | high |
| GSM2235635 | 5.917808219 | 0 | 1.689109588 | high |
| GSM2235636 | 5.671232877 | 0 | 1.745356863 | high |
| GSM2235696 | 8.876712329 | 0 | 0.088516756 | low |
| GSM2235697 | 3.205479452 | 1 | 1.917628047 | high |
| GSM2235698 | 1.890410959 | 1 | 3.79820988 | high |
| GSM2235704 | 8.95890411 | 0 | 2.668783312 | high |
| GSM2235705 | 9.369863014 | 0 | 1.722776513 | high |
| GSM2235707 | 9.534246575 | 0 | 0.652664096 | low |
| GSM2235708 | 8.95890411 | 0 | 0.250125966 | low |
| GSM2235709 | 8.876712329 | 0 | 0.388589309 | low |
| GSM2235715 | 9.698630137 | 0 | 1.113892455 | high |
| GSM2235716 | 8.547945205 | 1 | 0.877399884 | low |
| GSM2235719 | 0.082191781 | 1 | 13.67352257 | high |
| GSM2235721 | 9.780821918 | 0 | 1.806391513 | high |
| GSM2235722 | 2.95890411 | 1 | 0.780883789 | low |
| GSM2235724 | 0.821917808 | 1 | 5.459844321 | high |
| GSM2235726 | 0.082191781 | 1 | 0.700678849 | low |
| GSM2235728 | 2.876712329 | 1 | 0.836829666 | low |
| GSM2235729 | 0.410958904 | 1 | 2.035946784 | high |
| GSM2235730 | 4.02739726 | 1 | 1.816943737 | high |
| GSM2235732 | 9.698630137 | 0 | 0.533345943 | low |
| GSM2235733 | 11.83561644 | 0 | 0.812879891 | low |
| GSM2235735 | 11.91780822 | 0 | 0.687236857 | low |
| GSM2235738 | 12.57534247 | 0 | 0.116472517 | low |
| GSM2235739 | 1.150684932 | 1 | 7.410311842 | high |
| GSM2235742 | 0.246575342 | 1 | 12.7885611 | high |
| GSM2235747 | 0.98630137 | 1 | 1.023346879 | high |
| GSM2235751 | 8.301369863 | 0 | 0.400084411 | low |
| GSM2235753 | 2.547945205 | 1 | 3.08142877 | high |
| GSM2235757 | 3.287671233 | 1 | 1.954426783 | high |
| GSM2235759 | 0.98630137 | 1 | 6.604198369 | high |
| GSM2235763 | 8.95890411 | 1 | 0.425334776 | low |
| GSM2235765 | 11.34246575 | 0 | 2.601971388 | high |
| GSM2235766 | 1.232876712 | 1 | 5.432086179 | high |
| GSM2235767 | 1.479452055 | 1 | 4.228693654 | high |
| GSM2235768 | 11.83561644 | 0 | 0.75641796 | low |
| GSM2235770 | 0.575342466 | 1 | 7.909743533 | high |
| GSM2235772 | 4.931506849 | 1 | 1.496895291 | high |
| GSM2235773 | 1.397260274 | 1 | 2.408034078 | high |
| GSM2235774 | 0.98630137 | 1 | 4.658525693 | high |
| GSM2235775 | 9.863013699 | 0 | 4.061366018 | high |
| GSM2235776 | 1.643835616 | 1 | 2.167749947 | high |
| GSM2235779 | 4.02739726 | 1 | 0.404475003 | low |
| GSM2235783 | 6.164383562 | 1 | 2.373948143 | high |
| GSM2235786 | 8.876712329 | 0 | 0.936003271 | low |
| GSM2235787 | 2.054794521 | 1 | 2.446552674 | high |
| GSM2235790 | 9.780821918 | 0 | 1.232844179 | high |
| GSM2235791 | 2.054794521 | 1 | 1.569060123 | high |
| GSM2235794 | 9.945205479 | 0 | 0.477941331 | low |
| GSM2235795 | 9.945205479 | 0 | 0.751681414 | low |
| GSM2235797 | 9.452054795 | 0 | 0.393073322 | low |
| GSM2235799 | 0.98630137 | 1 | 6.964177763 | high |
| GSM2235803 | 8.794520548 | 0 | 0.066004433 | low |
| GSM2235805 | 0.410958904 | 1 | 9.931570916 | high |
| GSM2235807 | 2.712328767 | 1 | 1.345325398 | high |
| GSM2235810 | 0.575342466 | 1 | 0.360277474 | low |
| GSM2235813 | 2.136986301 | 1 | 1.732782104 | high |
| GSM2235814 | 9.287671233 | 0 | 0.535255594 | low |
| GSM2235815 | 9.287671233 | 0 | 0.132053289 | low |
| GSM2235816 | 0.328767123 | 1 | 3.895625198 | high |
| GSM2235819 | 1.726027397 | 1 | 1.810986527 | high |
| GSM2235821 | 9.205479452 | 0 | 0.664984897 | low |
| GSM2235822 | 9.205479452 | 0 | 0.293765047 | low |
| GSM2235823 | 0.739726027 | 1 | 2.210807417 | high |
| GSM2235824 | 6.493150685 | 1 | 4.86579283 | high |
| GSM2235831 | 3.205479452 | 1 | 3.018031797 | high |
| GSM2235838 | 0.328767123 | 1 | 13.31238344 | high |
| GSM2235839 | 8.301369863 | 1 | 1.404240737 | high |
| GSM2235841 | 1.315068493 | 1 | 2.591223794 | high |
| GSM2235843 | 1.068493151 | 1 | 7.039482696 | high |
| GSM2235846 | 7.561643836 | 1 | 1.737639835 | high |
| GSM2235850 | 9.534246575 | 0 | 0.434080165 | low |
| GSM2235851 | 7.643835616 | 1 | 0.498428348 | low |
| GSM2235852 | 3.945205479 | 1 | 1.074966136 | high |
| GSM2235853 | 9.123287671 | 0 | 0.250036424 | low |
| GSM2235854 | 9.123287671 | 0 | 0.224652863 | low |
| GSM2235863 | 10.93150685 | 0 | 1.036214399 | high |
| GSM2235864 | 0.98630137 | 1 | 2.447778984 | high |
| GSM2235865 | 2.712328767 | 1 | 2.783298283 | high |
| GSM2235866 | 10.60273973 | 0 | 3.618117414 | high |
| GSM2235867 | 10.2739726 | 0 | 0.69471921 | low |
| GSM2235878 | 10.2739726 | 0 | 1.081247585 | high |
| GSM2235879 | 0.493150685 | 1 | 18.34632175 | high |
| GSM2235880 | 1.397260274 | 1 | 2.123902559 | high |
| GSM2235881 | 10.2739726 | 0 | 1.448231093 | high |
| GSM2235883 | 1.561643836 | 1 | 3.690454683 | high |
| GSM2235902 | 2.794520548 | 1 | 3.692794294 | high |
| GSM2235906 | 9.863013699 | 1 | 1.428817633 | high |
| GSM2235907 | 10.10958904 | 0 | 0.324823341 | low |
| GSM2235909 | 1.479452055 | 1 | 1.569296502 | high |
| GSM2235910 | 11.42465753 | 0 | 0.5924372 | low |
| GSM2235922 | 11.34246575 | 0 | 0.990599062 | low |
| GSM2235923 | 1.150684932 | 1 | 2.569057382 | high |
| GSM2235925 | 11.01369863 | 0 | 0.482290851 | low |
| GSM2235927 | 0.003 | 1 | 2.642318047 | high |
| GSM2235929 | 4.931506849 | 1 | 1.66670709 | high |
| GSM2235934 | 3.863013699 | 0 | 0.843078972 | low |
| GSM2235935 | 1.561643836 | 1 | 1.179621242 | high |
| GSM2235938 | 7.561643836 | 0 | 1.067119284 | high |
| GSM2235939 | 3.698630137 | 0 | 0.851728766 | low |
| GSM2235940 | 2.712328767 | 1 | 0.526684285 | low |
| GSM2235945 | 3.04109589 | 1 | 4.396083006 | high |
| GSM2235948 | 10.84931507 | 0 | 0.543658645 | low |
| GSM2235949 | 10.84931507 | 0 | 0.295350886 | low |
| GSM2235950 | 10.52054795 | 0 | 0.714935877 | low |
| GSM2235952 | 0.082191781 | 1 | 4.850438024 | high |
| GSM2235954 | 12.24657534 | 0 | 0.410254458 | low |
| GSM2235957 | 12.16438356 | 0 | 0.59632056 | low |
| GSM2235961 | 11.5890411 | 0 | 0.365278969 | low |
| GSM2235962 | 2.219178082 | 1 | 1.344698159 | high |
| GSM2235972 | 4.684931507 | 0 | 0.325103443 | low |
| GSM2235974 | 0.98630137 | 1 | 6.797658826 | high |
| GSM2235975 | 5.01369863 | 0 | 0.956145728 | low |
| GSM2235976 | 4.520547945 | 0 | 0.18404339 | low |
| GSM2235977 | 0.575342466 | 1 | 10.1066153 | high |
| GSM2235978 | 2.794520548 | 0 | 0.249111739 | low |
| GSM2235979 | 3.452054795 | 0 | 0.315886174 | low |
| GSM2235984 | 0.657534247 | 1 | 2.472691095 | high |
| GSM2235988 | 5.753424658 | 0 | 0.308096462 | low |
| GSM2235993 | 10.35616438 | 0 | 0.400716002 | low |
| GSM2235994 | 10.10958904 | 0 | 1.00088676 | high |
| GSM2235995 | 0.821917808 | 1 | 0.424230273 | low |
| GSM2235996 | 0.493150685 | 1 | 9.327721681 | high |
| GSM2235997 | 10.52054795 | 0 | 0.686193618 | low |
| GSM2236001 | 10.19178082 | 0 | 1.124296118 | high |
| GSM2236004 | 3.780821918 | 1 | 0.197063451 | low |
| GSM2236006 | 1.97260274 | 1 | 4.232384321 | high |
| GSM2236015 | 9.04109589 | 0 | 0.522618238 | low |
| GSM2236018 | 9.534246575 | 0 | 0.708807596 | low |
| GSM2236019 | 7.561643836 | 1 | 1.594080613 | high |
| GSM2236021 | 4.191780822 | 1 | 2.923292587 | high |
| GSM2236022 | 8.547945205 | 0 | 1.443253779 | high |
| GSM2236024 | 8.465753425 | 0 | 0.897528934 | low |
| GSM2236025 | 3.205479452 | 1 | 1.621777672 | high |
| GSM2236026 | 5.260273973 | 1 | 4.538741894 | high |
| GSM2236027 | 10.19178082 | 0 | 1.950699309 | high |
| GSM2236028 | 7.808219178 | 1 | 0.641008598 | low |
| GSM2236032 | 11.01369863 | 0 | 0.037550602 | low |
| GSM2236034 | 10.02739726 | 0 | 0.37996961 | low |
| GSM2236038 | 3.287671233 | 0 | 4.636211352 | high |
| GSM2236039 | 3.452054795 | 0 | 1.25210386 | high |
| GSM2236040 | 3.04109589 | 0 | 0.327703151 | low |
| GSM2236041 | 3.369863014 | 0 | 0.511543063 | low |
| GSM2236046 | 2.876712329 | 1 | 3.125553882 | high |
| GSM2236048 | 8.383561644 | 0 | 0.060539419 | low |
| GSM2236050 | 8.301369863 | 0 | 0.159204441 | low |
| GSM2236052 | 9.534246575 | 0 | 0.856724567 | low |
| GSM2236053 | 1.808219178 | 1 | 3.606387999 | high |
| GSM2236054 | 6.493150685 | 1 | 0.706962629 | low |
| GSM2236056 | 10.52054795 | 0 | 0.218345502 | low |
| GSM2236057 | 3.04109589 | 1 | 1.139050638 | high |
| GSM2236059 | 11.26027397 | 0 | 1.715967434 | high |
| GSM2236060 | 3.04109589 | 1 | 0.986870074 | low |
| GSM2236062 | 10.60273973 | 0 | 0.135932733 | low |
| GSM2236063 | 10.76712329 | 0 | 1.178790821 | high |
| GSM2236064 | 6.328767123 | 1 | 0.63425009 | low |
| GSM2236067 | 1.397260274 | 1 | 2.866706258 | high |
| GSM2236070 | 10.19178082 | 0 | 0.314187078 | low |
| GSM2236071 | 9.369863014 | 0 | 0.250341445 | low |
| GSM2236072 | 2.712328767 | 1 | 1.497798016 | high |
| GSM2236077 | 3.123287671 | 0 | 0.394085719 | low |
| GSM2236080 | 1.150684932 | 1 | 0.801714806 | low |
| GSM2236082 | 10.76712329 | 0 | 0.305447288 | low |
| GSM2236083 | 0.575342466 | 1 | 1.436619444 | high |
| GSM2236086 | 12.24657534 | 0 | 0.219393459 | low |
| GSM2236089 | 11.83561644 | 0 | 0.068356556 | low |
| GSM2236091 | 10.10958904 | 1 | 0.513181362 | low |
| GSM2236092 | 12.24657534 | 0 | 0.6505073 | low |
| GSM2236093 | 12.4109589 | 0 | 0.197775399 | low |
| GSM2236095 | 12.32876712 | 0 | 0.270260439 | low |
| TCGA-VQ-A8DU | 0.454794521 | 1 | 0.298209565 | low |
| TCGA-HU-A4G9 | 2.016438356 | 0 | 0.790984937 | low |
| TCGA-VQ-A91S | 2.739726027 | 0 | 0.625059567 | low |
| TCGA-BR-8682 | 2.715068493 | 0 | 12.1045006 | high |
| TCGA-D7-6524 | 1.487671233 | 0 | 2.324874607 | high |
| TCGA-RD-A7BT | 0.717808219 | 1 | 0.36321981 | low |
| TCGA-IN-A7NT | 0.884931507 | 0 | 2.140261286 | high |
| TCGA-BR-8296 | 1.298630137 | 1 | 3.467697653 | high |
| TCGA-BR-4187 | 0.38630137 | 1 | 7.043347125 | high |
| TCGA-MX-A5UJ | 1.643835616 | 0 | 1.134373164 | high |
| TCGA-BR-8286 | 2.452054795 | 0 | 0.6554694 | low |
| TCGA-VQ-A8E7 | 3.117808219 | 0 | 1.560593808 | high |
| TCGA-D7-6520 | 1.569863014 | 0 | 1.119442933 | high |
| TCGA-BR-4368 | 0.003 | 0 | 2.639135829 | high |
| TCGA-BR-6457 | 1.139726027 | 0 | 4.878311083 | high |
| TCGA-D7-A6F2 | 1.304109589 | 0 | 0.973547448 | low |
| TCGA-BR-6454 | 0.003 | 0 | 1.087674061 | high |
| TCGA-VQ-A91X | 0.791780822 | 1 | 0.728596181 | low |
| TCGA-IN-A6RN | 1.62739726 | 0 | 0.802673734 | low |
| TCGA-VQ-A928 | 0.476712329 | 1 | 1.051647944 | high |
| TCGA-BR-7958 | 2.463013699 | 0 | 0.825738559 | low |
| TCGA-CD-A48C | 0.967123288 | 1 | 1.755499913 | high |
| TCGA-IN-AB1V | 1.312328767 | 0 | 0.667193521 | low |
| TCGA-CD-8532 | 0.969863014 | 1 | 1.852694148 | high |
| TCGA-IN-8663 | 0.282191781 | 1 | 11.01622666 | high |
| TCGA-VQ-A8PM | 0.156164384 | 1 | 1.456353227 | high |
| TCGA-B7-A5TJ | 0.917808219 | 0 | 1.654955575 | high |
| TCGA-IN-A6RS | 1.049315068 | 0 | 0.567167213 | low |
| TCGA-BR-7717 | 1.512328767 | 1 | 4.440019633 | high |
| TCGA-IN-A6RR | 0.561643836 | 1 | 2.044847645 | high |
| TCGA-BR-8592 | 0.523287671 | 1 | 7.357786701 | high |
| TCGA-BR-6453 | 1.328767123 | 0 | 1.122454577 | high |
| TCGA-HU-A4GP | 0.747945205 | 0 | 0.781890527 | low |
| TCGA-HU-A4H8 | 0.739726027 | 0 | 0.094773691 | low |
| TCGA-VQ-A8E3 | 1.810958904 | 1 | 0.23082065 | low |
| TCGA-BR-8297 | 0.616438356 | 0 | 6.286025884 | high |
| TCGA-IN-A7NR | 0.542465753 | 0 | 1.155266801 | high |
| TCGA-HU-A4G8 | 1.890410959 | 0 | 0.268019208 | low |
| TCGA-CG-5724 | 1.002739726 | 1 | 4.75061408 | high |
| TCGA-BR-4201 | 2.575342466 | 1 | 5.860387103 | high |
| TCGA-CD-5799 | 1.084931507 | 0 | 1.309652879 | high |
| TCGA-HU-A4H0 | 0.175342466 | 0 | 0.312629567 | low |
| TCGA-FP-8631 | 0.046575342 | 0 | 10.95124762 | high |
| TCGA-BR-4279 | 0.797260274 | 1 | 3.13384617 | high |
| TCGA-IN-A6RJ | 1.038356164 | 0 | 0.400492708 | low |
| TCGA-CD-5800 | 1.095890411 | 0 | 0.444383309 | low |
| TCGA-CG-4442 | 0.003 | 0 | 9.16888852 | high |
| TCGA-CD-5803 | 0.934246575 | 1 | 4.314001516 | high |
| TCGA-BR-8371 | 0.983561644 | 1 | 2.928353655 | high |
| TCGA-VQ-A8PU | 2.279452055 | 1 | 1.462038178 | high |
| TCGA-VQ-A8PD | 1.35890411 | 1 | 2.613344104 | high |
| TCGA-VQ-A922 | 0.753424658 | 1 | 3.577853354 | high |
| TCGA-FP-8210 | 0.419178082 | 1 | 2.225439383 | high |
| TCGA-RD-A8N0 | 3.38630137 | 0 | 0.625227879 | low |
| TCGA-BR-8289 | 0.221917808 | 1 | 2.129980767 | high |
| TCGA-HU-A4H3 | 2.416438356 | 0 | 1.942664586 | high |
| TCGA-BR-7716 | 3.315068493 | 0 | 3.358666721 | high |
| TCGA-HU-8608 | 1.756164384 | 0 | 0.042293506 | low |
| TCGA-F1-A72C | 0.947945205 | 0 | 1.743023205 | high |
| TCGA-VQ-A8P3 | 3.101369863 | 0 | 0.351680446 | low |
| TCGA-VQ-A91N | 1.561643836 | 1 | 0.380925407 | low |
| TCGA-EQ-8122 | 0.665753425 | 1 | 1.961978733 | high |
| TCGA-CG-4438 | 4.506849315 | 0 | 1.991459392 | high |
| TCGA-BR-4366 | 0.003 | 0 | 4.570608463 | high |
| TCGA-ZQ-A9CR | 0.065753425 | 1 | 1.243645179 | high |
| TCGA-BR-8690 | 0.890410959 | 0 | 3.141037987 | high |
| TCGA-BR-4357 | 0.003 | 0 | 0.45239624 | low |
| TCGA-CG-4440 | 0.334246575 | 1 | 3.987135985 | high |
| TCGA-BR-6802 | 2.575342466 | 0 | 0.156670687 | low |
| TCGA-BR-4370 | 0.003 | 0 | 0.925128493 | low |
| TCGA-VQ-A8E2 | 3.61369863 | 0 | 2.613360708 | high |
| TCGA-D7-6818 | 1.030136986 | 1 | 15.08994972 | high |
| TCGA-D7-6525 | 1.112328767 | 1 | 37.48460603 | high |
| TCGA-BR-7959 | 2.767123288 | 0 | 0.886416041 | low |
| TCGA-CG-4301 | 0.252054795 | 0 | 3.875530957 | high |
| TCGA-RD-A8MV | 10.19178082 | 0 | 0.836655234 | low |
| TCGA-HU-A4GC | 0.271232877 | 0 | 0.286737649 | low |
| TCGA-BR-6564 | 2.175342466 | 1 | 2.762097446 | high |
| TCGA-HU-A4GU | 0.547945205 | 0 | 0.128022667 | low |
| TCGA-CD-8528 | 1.02739726 | 0 | 0.869197832 | low |
| TCGA-BR-8291 | 1.663013699 | 1 | 12.19572555 | high |
| TCGA-D7-6526 | 1.432876712 | 0 | 0.534493092 | low |
| TCGA-BR-8364 | 1.849315068 | 0 | 8.183281845 | high |
| TCGA-F1-6874 | 1.205479452 | 0 | 0.719445472 | low |
| TCGA-VQ-A91U | 0.142465753 | 1 | 0.398885368 | low |
| TCGA-VQ-A925 | 0.378082192 | 1 | 0.83850885 | low |
| TCGA-F1-6875 | 6.019178082 | 1 | 0.342820031 | low |
| TCGA-BR-8589 | 2.260273973 | 0 | 0.116602666 | low |
| TCGA-3M-AB46 | 4.835616438 | 0 | 0.368165539 | low |
| TCGA-BR-4294 | 0.003 | 0 | 2.148032984 | high |
| TCGA-VQ-A924 | 4.619178082 | 1 | 0.12114722 | low |
| TCGA-VQ-A8PK | 1.487671233 | 1 | 1.54877869 | high |
| TCGA-R5-A7ZE | 1.517808219 | 1 | 0.565139683 | low |
| TCGA-D7-6822 | 1.02739726 | 0 | 0.370533114 | low |
| TCGA-BR-6563 | 3.260273973 | 0 | 4.658062428 | high |
| TCGA-SW-A7EA | 1.58630137 | 0 | 0.649437375 | low |
| TCGA-BR-8680 | 2.663013699 | 0 | 0.960451764 | low |
| TCGA-RD-A8N5 | 4.78630137 | 1 | 0.770736564 | low |
| TCGA-FP-8209 | 4.961643836 | 1 | 3.114474501 | high |
| TCGA-BR-7722 | 1.276712329 | 1 | 2.063441525 | high |
| TCGA-CD-5798 | 1.117808219 | 0 | 3.002883137 | high |
| TCGA-VQ-A8P2 | 3.178082192 | 0 | 0.235364066 | low |
| TCGA-BR-8687 | 0.684931507 | 1 | 1.262334968 | high |
| TCGA-D7-A4Z0 | 1.230136986 | 0 | 1.381488854 | high |
| TCGA-VQ-A923 | 0.003 | 1 | 0.21688502 | low |
| TCGA-D7-A4YU | 1.369863014 | 0 | 0.539197542 | low |
| TCGA-BR-8679 | 0.003 | 0 | 0.747353058 | low |
| TCGA-D7-8574 | 1.432876712 | 0 | 1.23154962 | high |
| TCGA-D7-A748 | 0.361643836 | 1 | 3.747211914 | high |
| TCGA-BR-A4J7 | 2.709589041 | 0 | 1.50175967 | high |
| TCGA-BR-6852 | 3.745205479 | 0 | 0.324747697 | low |
| TCGA-BR-6458 | 1.610958904 | 1 | 1.692661623 | high |
| TCGA-ZA-A8F6 | 1.438356164 | 0 | 1.102256122 | high |
| TCGA-BR-7196 | 1.824657534 | 0 | 3.385552786 | high |
| TCGA-IN-A6RI | 1.531506849 | 0 | 0.277581684 | low |
| TCGA-B7-5818 | 0.975342466 | 0 | 0.458884719 | low |
| TCGA-BR-A4J6 | 0.054794521 | 0 | 2.45555504 | high |
| TCGA-CG-5718 | 3 | 1 | 0.451928149 | low |
| TCGA-BR-6455 | 1.156164384 | 1 | 0.767861072 | low |
| TCGA-BR-4363 | 0.003 | 0 | 2.706596221 | high |
| TCGA-VQ-A8P5 | 0.643835616 | 1 | 2.992876901 | high |
| TCGA-HU-8249 | 2.41369863 | 0 | 0.236240042 | low |
| TCGA-HU-A4H4 | 1.98630137 | 0 | 0.088591838 | low |
| TCGA-VQ-A8PJ | 0.224657534 | 1 | 1.609019043 | high |
| TCGA-BR-A44T | 2.843835616 | 0 | 0.447976146 | low |
| TCGA-CD-5813 | 1.032876712 | 1 | 2.615165211 | high |
| TCGA-CG-5717 | 0.580821918 | 1 | 0.513497356 | low |
| TCGA-BR-A4J8 | 1.126027397 | 0 | 1.695648843 | high |
| TCGA-CG-4477 | 2.580821918 | 0 | 0.415543286 | low |
| TCGA-CD-A489 | 0.942465753 | 1 | 4.796604156 | high |
| TCGA-RD-A8N4 | 5.947945205 | 0 | 0.817912413 | low |
| TCGA-RD-A8N1 | 9.64109589 | 0 | 0.972154689 | low |
| TCGA-BR-8487 | 0.093150685 | 0 | 0.92814712 | low |
| TCGA-BR-7957 | 0.756164384 | 1 | 6.007693287 | high |
| TCGA-BR-A4CS | 0.123287671 | 1 | 0.948174898 | low |
| TCGA-IP-7968 | 0.210958904 | 0 | 3.519632859 | high |
| TCGA-IN-7808 | 0.287671233 | 1 | 0.452657729 | low |
| TCGA-D7-8572 | 1.4 | 0 | 3.534254756 | high |
| TCGA-CD-A48A | 1.035616438 | 0 | 0.395702297 | low |
| TCGA-VQ-A8PC | 3.854794521 | 1 | 0.998265532 | low |
| TCGA-HU-A4HD | 2.783561644 | 0 | 2.352946861 | high |
| TCGA-BR-8369 | 1.169863014 | 0 | 3.065604235 | high |
| TCGA-BR-A4J5 | 2.361643836 | 0 | 5.371294962 | high |
| TCGA-HF-7133 | 5.254794521 | 0 | 0.711803841 | low |
| TCGA-IN-7806 | 3.030136986 | 0 | 1.464922465 | high |
| TCGA-BR-4361 | 0.003 | 0 | 1.223098567 | high |
| TCGA-IN-A7NU | 0.975342466 | 0 | 3.027118976 | high |
| TCGA-RD-A7BW | 0.42739726 | 1 | 7.232506079 | high |
| TCGA-BR-8486 | 0.003 | 0 | 0.503643891 | low |
| TCGA-VQ-AA68 | 3.638356164 | 0 | 1.302484514 | high |
| TCGA-VQ-A8P8 | 2.580821918 | 0 | 0.395493586 | low |
| TCGA-VQ-A92D | 5.567123288 | 0 | 0.4020956 | low |
| TCGA-HU-A4H2 | 1.079452055 | 0 | 0.234152183 | low |
| TCGA-VQ-A91Z | 4.630136986 | 0 | 0.681026954 | low |
| TCGA-D7-A6EX | 0.942465753 | 0 | 0.098257725 | low |
| TCGA-VQ-A927 | 0.547945205 | 1 | 0.566170623 | low |
| TCGA-BR-4257 | 0.805479452 | 1 | 2.208819245 | high |
| TCGA-RD-A7C1 | 1.389041096 | 1 | 2.993070683 | high |
| TCGA-BR-7901 | 0.287671233 | 1 | 1.383069678 | high |
| TCGA-BR-7715 | 2.802739726 | 0 | 0.40611781 | low |
| TCGA-D7-A74A | 1.663013699 | 0 | 2.635508717 | high |
| TCGA-BR-4253 | 0.339726027 | 1 | 0.233470879 | low |
| TCGA-HU-A4GD | 1.895890411 | 0 | 2.550600504 | high |
| TCGA-VQ-A8DV | 1.104109589 | 1 | 4.63051068 | high |
| TCGA-VQ-A8PH | 1.065753425 | 1 | 6.543885886 | high |
| TCGA-R5-A7ZR | 0.506849315 | 1 | 0.358001956 | low |
| TCGA-VQ-AA6A | 3.243835616 | 0 | 0.196585674 | low |
| TCGA-BR-8686 | 1.739726027 | 1 | 1.538663551 | high |
| TCGA-FP-8211 | 1.131506849 | 0 | 0.149523504 | low |
| TCGA-FP-7735 | 0.290410959 | 1 | 0.890549348 | low |
| TCGA-HU-A4GH | 0.980821918 | 0 | 0.148544695 | low |
| TCGA-CG-4437 | 0.671232877 | 0 | 2.229670718 | high |
| TCGA-CD-8530 | 1.032876712 | 0 | 3.776048453 | high |
| TCGA-CD-8534 | 1.005479452 | 0 | 0.528992352 | low |
| TCGA-VQ-A94P | 0.221917808 | 1 | 1.404777347 | high |
| GSM2235556 | 5.753424658 | 1 | 6.617307136 | high |
| GSM2235557 | 1.890410959 | 1 | 2.358847668 | high |
| GSM2235558 | 1.97260274 | 1 | 0.313008767 | low |
| GSM2235559 | 4.849315068 | 1 | 2.062965 | high |
| GSM2235560 | 0.98630137 | 1 | 5.764251522 | high |
| GSM2235561 | 6.98630137 | 0 | 2.735073177 | high |
| GSM2235562 | 1.479452055 | 1 | 1.848585868 | high |
| GSM2235564 | 6.904109589 | 0 | 0.959087612 | low |
| GSM2235565 | 5.342465753 | 1 | 2.211038958 | high |
| GSM2235568 | 6.739726027 | 0 | 2.173032274 | high |
| GSM2235570 | 6.657534247 | 0 | 4.67912625 | high |
| GSM2235571 | 6.575342466 | 0 | 2.936521072 | high |
| GSM2235572 | 7.068493151 | 0 | 1.363684606 | high |
| GSM2235578 | 1.726027397 | 1 | 0.160618838 | low |
| GSM2235580 | 2.054794521 | 1 | 1.139049359 | high |
| GSM2235584 | 4.684931507 | 1 | 0.382576638 | low |
| GSM2235585 | 1.890410959 | 1 | 0.131303652 | low |
| GSM2235588 | 4.191780822 | 1 | 0.818586929 | low |
| GSM2235589 | 6.328767123 | 0 | 1.484196881 | high |
| GSM2235603 | 6.575342466 | 0 | 0.813997932 | low |
| GSM2235605 | 5.506849315 | 1 | 0.256842328 | low |
| GSM2235606 | 6 | 0 | 0.992221162 | low |
| GSM2235608 | 6.575342466 | 0 | 0.747395292 | low |
| GSM2235609 | 0.657534247 | 1 | 0.549988599 | low |
| GSM2235612 | 2.383561644 | 1 | 1.381469956 | high |
| GSM2235613 | 6.821917808 | 0 | 3.781676281 | high |
| GSM2235614 | 6.328767123 | 0 | 0.399629838 | low |
| GSM2235615 | 6.328767123 | 0 | 1.164570259 | high |
| GSM2235616 | 0.082191781 | 1 | 1.243132035 | high |
| GSM2235618 | 5.835616438 | 0 | 0.331915365 | low |
| GSM2235622 | 1.890410959 | 1 | 1.133159745 | high |
| GSM2235624 | 6.328767123 | 0 | 0.716868469 | low |
| GSM2235626 | 6.328767123 | 0 | 1.044590184 | high |
| GSM2235627 | 5.753424658 | 0 | 0.116521914 | low |
| GSM2235628 | 6.082191781 | 0 | 0.543254704 | low |
| GSM2235629 | 2.95890411 | 1 | 0.719542136 | low |
| GSM2235631 | 5.671232877 | 0 | 1.217361427 | high |
| GSM2235637 | 5.589041096 | 0 | 0.352221393 | low |
| GSM2235695 | 9.945205479 | 0 | 8.814300184 | high |
| GSM2235699 | 1.890410959 | 1 | 10.51307417 | high |
| GSM2235700 | 9.616438356 | 0 | 1.472033061 | high |
| GSM2235701 | 8.876712329 | 0 | 0.778607751 | low |
| GSM2235702 | 1.479452055 | 1 | 5.262992053 | high |
| GSM2235703 | 3.534246575 | 1 | 3.562847515 | high |
| GSM2235706 | 0.164383562 | 1 | 5.507377386 | high |
| GSM2235710 | 1.068493151 | 1 | 0.984454842 | low |
| GSM2235711 | 1.561643836 | 1 | 1.665821771 | high |
| GSM2235712 | 8.876712329 | 0 | 0.574107608 | low |
| GSM2235713 | 9.534246575 | 1 | 2.536944124 | high |
| GSM2235714 | 3.780821918 | 1 | 1.504187692 | high |
| GSM2235717 | 0.246575342 | 1 | 1.710704791 | high |
| GSM2235718 | 9.780821918 | 0 | 0.487023314 | low |
| GSM2235720 | 9.780821918 | 0 | 0.892242337 | low |
| GSM2235723 | 9.698630137 | 0 | 0.924964772 | low |
| GSM2235725 | 10.02739726 | 0 | 1.941517967 | high |
| GSM2235727 | 9.698630137 | 0 | 0.678819239 | low |
| GSM2235731 | 9.698630137 | 0 | 0.292844932 | low |
| GSM2235734 | 11.83561644 | 0 | 1.985388559 | high |
| GSM2235736 | 0.739726027 | 1 | 1.069132456 | high |
| GSM2235737 | 3.698630137 | 1 | 1.209725417 | high |
| GSM2235740 | 0.328767123 | 1 | 0.0599456 | low |
| GSM2235741 | 10.43835616 | 0 | 2.840577449 | high |
| GSM2235743 | 0.328767123 | 1 | 1.944640397 | high |
| GSM2235744 | 4.02739726 | 1 | 0.648990126 | low |
| GSM2235745 | 8.712328767 | 0 | 0.758511586 | low |
| GSM2235746 | 9.123287671 | 0 | 0.26166571 | low |
| GSM2235748 | 0.164383562 | 1 | 8.184097236 | high |
| GSM2235749 | 3.205479452 | 1 | 2.632485334 | high |
| GSM2235750 | 0.575342466 | 1 | 0.765734007 | low |
| GSM2235752 | 12.24657534 | 0 | 0.236705817 | low |
| GSM2235754 | 11.34246575 | 0 | 1.825501387 | high |
| GSM2235755 | 7.150684932 | 1 | 4.88978353 | high |
| GSM2235756 | 11.50684932 | 0 | 0.280830358 | low |
| GSM2235758 | 1.068493151 | 1 | 1.49979244 | high |
| GSM2235760 | 1.643835616 | 1 | 3.829528237 | high |
| GSM2235761 | 11.01369863 | 0 | 1.702740307 | high |
| GSM2235762 | 3.205479452 | 1 | 0.219949205 | low |
| GSM2235764 | 0.739726027 | 1 | 6.54446842 | high |
| GSM2235769 | 1.808219178 | 1 | 12.15489213 | high |
| GSM2235771 | 11.75342466 | 0 | 3.084302005 | high |
| GSM2235777 | 9.452054795 | 1 | 1.288504165 | high |
| GSM2235778 | 1.890410959 | 1 | 3.280205981 | high |
| GSM2235780 | 0.003 | 1 | 1.412394054 | high |
| GSM2235781 | 9.452054795 | 0 | 2.646846085 | high |
| GSM2235782 | 0.328767123 | 1 | 13.9346857 | high |
| GSM2235784 | 8.465753425 | 1 | 0.17960775 | low |
| GSM2235785 | 9.369863014 | 0 | 0.031242656 | low |
| GSM2235788 | 9.616438356 | 0 | 0.10725682 | low |
| GSM2235789 | 3.04109589 | 1 | 1.411801189 | high |
| GSM2235792 | 7.561643836 | 1 | 1.579087055 | high |
| GSM2235793 | 7.150684932 | 1 | 1.588095276 | high |
| GSM2235796 | 3.616438356 | 1 | 1.85500929 | high |
| GSM2235798 | 2.054794521 | 1 | 1.14541557 | high |
| GSM2235800 | 9.369863014 | 0 | 3.794219471 | high |
| GSM2235801 | 9.287671233 | 0 | 2.783548432 | high |
| GSM2235802 | 8.712328767 | 0 | 0.27249341 | low |
| GSM2235804 | 9.369863014 | 0 | 1.13528894 | high |
| GSM2235806 | 8.301369863 | 1 | 35.22115459 | high |
| GSM2235808 | 1.890410959 | 1 | 9.494766772 | high |
| GSM2235809 | 12 | 0 | 0.351116857 | low |
| GSM2235811 | 0.98630137 | 1 | 1.358561354 | high |
| GSM2235812 | 2.794520548 | 1 | 0.491125678 | low |
| GSM2235817 | 4.520547945 | 1 | 1.067148977 | high |
| GSM2235818 | 0.821917808 | 1 | 0.88589833 | low |
| GSM2235820 | 1.97260274 | 1 | 1.563478013 | high |
| GSM2235825 | 1.890410959 | 1 | 2.702066616 | high |
| GSM2235826 | 0.904109589 | 1 | 0.078876499 | low |
| GSM2235827 | 3.534246575 | 1 | 1.418531155 | high |
| GSM2235828 | 8.794520548 | 0 | 0.249416751 | low |
| GSM2235829 | 8.95890411 | 0 | 0.614143061 | low |
| GSM2235830 | 8.054794521 | 0 | 1.600100886 | high |
| GSM2235837 | 9.287671233 | 0 | 3.860036866 | high |
| GSM2235840 | 7.561643836 | 1 | 0.379919699 | low |
| GSM2235842 | 1.150684932 | 1 | 1.280075488 | high |
| GSM2235844 | 8.383561644 | 0 | 0.235698122 | low |
| GSM2235845 | 2.547945205 | 1 | 0.802893021 | low |
| GSM2235847 | 9.123287671 | 0 | 0.119839873 | low |
| GSM2235848 | 3.287671233 | 1 | 5.190222316 | high |
| GSM2235849 | 0.082191781 | 1 | 1.720871105 | high |
| GSM2235855 | 9.04109589 | 0 | 0.206562544 | low |
| GSM2235862 | 6.98630137 | 1 | 0.932332918 | low |
| GSM2235882 | 1.068493151 | 1 | 0.953456134 | low |
| GSM2235884 | 10.35616438 | 0 | 0.497171745 | low |
| GSM2235885 | 10.19178082 | 0 | 2.107926392 | high |
| GSM2235886 | 4.191780822 | 1 | 1.090814259 | high |
| GSM2235887 | 6.493150685 | 1 | 1.420404752 | high |
| GSM2235888 | 5.589041096 | 1 | 6.815471504 | high |
| GSM2235889 | 7.643835616 | 1 | 0.424576702 | low |
| GSM2235899 | 2.794520548 | 1 | 0.97633376 | low |
| GSM2235900 | 10.43835616 | 0 | 2.113286436 | high |
| GSM2235901 | 4.767123288 | 1 | 2.734264895 | high |
| GSM2235903 | 10.76712329 | 0 | 0.775041273 | low |
| GSM2235904 | 1.726027397 | 1 | 2.097235145 | high |
| GSM2235905 | 0.164383562 | 1 | 1.721260589 | high |
| GSM2235908 | 10.10958904 | 0 | 0.554015243 | low |
| GSM2235921 | 11.42465753 | 0 | 1.49548623 | high |
| GSM2235924 | 11.09589041 | 0 | 0.911382422 | low |
| GSM2235926 | 11.50684932 | 0 | 0.636335631 | low |
| GSM2235928 | 10.84931507 | 0 | 2.699262348 | high |
| GSM2235930 | 6.082191781 | 1 | 0.885297838 | low |
| GSM2235931 | 10.84931507 | 0 | 3.94801213 | high |
| GSM2235932 | 4.849315068 | 0 | 0.748019252 | low |
| GSM2235933 | 3.369863014 | 0 | 2.661012381 | high |
| GSM2235936 | 3.452054795 | 0 | 0.262854609 | low |
| GSM2235937 | 3.04109589 | 0 | 0.288301226 | low |
| GSM2235941 | 3.369863014 | 0 | 0.127279832 | low |
| GSM2235942 | 3.452054795 | 0 | 0.236467031 | low |
| GSM2235943 | 10.43835616 | 0 | 1.043608316 | high |
| GSM2235944 | 10.35616438 | 0 | 1.242116991 | high |
| GSM2235946 | 10.76712329 | 0 | 0.446025123 | low |
| GSM2235947 | 10.60273973 | 0 | 0.116370065 | low |
| GSM2235951 | 0.410958904 | 1 | 5.904633645 | high |
| GSM2235953 | 0.821917808 | 1 | 2.549523563 | high |
| GSM2235955 | 12.24657534 | 0 | 0.602160864 | low |
| GSM2235956 | 11.09589041 | 1 | 0.661682555 | low |
| GSM2235958 | 12.49315068 | 0 | 1.780755319 | high |
| GSM2235959 | 11.83561644 | 0 | 2.515738393 | high |
| GSM2235960 | 11.67123288 | 0 | 0.538442381 | low |
| GSM2235963 | 2.219178082 | 1 | 1.102811978 | high |
| GSM2235964 | 1.808219178 | 1 | 3.202191232 | high |
| GSM2235965 | 5.178082192 | 1 | 5.730221055 | high |
| GSM2235966 | 2.712328767 | 0 | 0.985456124 | low |
| GSM2235967 | 0.821917808 | 1 | 0.303183676 | low |
| GSM2235968 | 4.849315068 | 0 | 0.30348405 | low |
| GSM2235969 | 5.260273973 | 0 | 0.12493788 | low |
| GSM2235970 | 1.479452055 | 1 | 4.373997732 | high |
| GSM2235971 | 5.178082192 | 0 | 3.888344725 | high |
| GSM2235973 | 5.095890411 | 0 | 1.099211706 | high |
| GSM2235980 | 0.493150685 | 1 | 9.539101546 | high |
| GSM2235981 | 2.95890411 | 0 | 0.150746592 | low |
| GSM2235982 | 3.04109589 | 0 | 1.22054185 | high |
| GSM2235983 | 3.123287671 | 0 | 0.582779803 | low |
| GSM2235985 | 2.876712329 | 0 | 3.370970024 | high |
| GSM2235986 | 2.876712329 | 0 | 0.605602794 | low |
| GSM2235987 | 2.876712329 | 0 | 1.572488507 | high |
| GSM2235991 | 2.630136986 | 1 | 1.962543511 | high |
| GSM2235992 | 10.52054795 | 0 | 0.458829716 | low |
| GSM2235998 | 10.02739726 | 0 | 1.86649468 | high |
| GSM2235999 | 7.808219178 | 1 | 0.35563845 | low |
| GSM2236000 | 10.02739726 | 0 | 4.22916964 | high |
| GSM2236002 | 10.60273973 | 0 | 1.07294357 | high |
| GSM2236003 | 10.35616438 | 0 | 1.253673428 | high |
| GSM2236005 | 0.739726027 | 1 | 3.44594761 | high |
| GSM2236007 | 1.726027397 | 1 | 0.667543642 | low |
| GSM2236008 | 2.465753425 | 1 | 1.480649813 | high |
| GSM2236009 | 2.219178082 | 1 | 2.872138079 | high |
| GSM2236010 | 3.123287671 | 1 | 1.943254356 | high |
| GSM2236011 | 2.136986301 | 1 | 1.7115638 | high |
| GSM2236012 | 0.246575342 | 1 | 2.156184687 | high |
| GSM2236013 | 4.356164384 | 1 | 1.072537808 | high |
| GSM2236014 | 8.630136986 | 1 | 0.799242923 | low |
| GSM2236016 | 1.643835616 | 1 | 0.841227282 | low |
| GSM2236017 | 1.397260274 | 1 | 1.328034595 | high |
| GSM2236020 | 8.95890411 | 0 | 0.089151378 | low |
| GSM2236023 | 8.876712329 | 0 | 0.07220796 | low |
| GSM2236029 | 11.34246575 | 0 | 1.238738417 | high |
| GSM2236030 | 11.34246575 | 0 | 0.600738427 | low |
| GSM2236031 | 11.01369863 | 0 | 1.06385941 | high |
| GSM2236033 | 9.863013699 | 0 | 1.087349822 | high |
| GSM2236036 | 3.369863014 | 0 | 0.928980392 | low |
| GSM2236037 | 3.369863014 | 0 | 3.515489201 | high |
| GSM2236042 | 3.04109589 | 0 | 1.431855413 | high |
| GSM2236043 | 3.287671233 | 0 | 0.426309887 | low |
| GSM2236044 | 11.5890411 | 0 | 17.16968542 | high |
| GSM2236045 | 13.23287671 | 0 | 2.505279039 | high |
| GSM2236047 | 4.02739726 | 1 | 4.028811894 | high |
| GSM2236049 | 2.712328767 | 1 | 0.94084857 | low |
| GSM2236051 | 3.123287671 | 1 | 3.516668902 | high |
| GSM2236055 | 10.93150685 | 0 | 0.758262897 | low |
| GSM2236058 | 11.17808219 | 0 | 0.292815927 | low |
| GSM2236061 | 10.60273973 | 0 | 0.275482738 | low |
| GSM2236065 | 9.780821918 | 0 | 2.652611069 | high |
| GSM2236066 | 10.43835616 | 0 | 5.107415214 | high |
| GSM2236068 | 3.452054795 | 1 | 1.070940099 | high |
| GSM2236069 | 9.452054795 | 0 | 1.18254339 | high |
| GSM2236073 | 1.808219178 | 1 | 0.690478489 | low |
| GSM2236074 | 11.75342466 | 0 | 0.087745448 | low |
| GSM2236075 | 3.205479452 | 1 | 0.622871838 | low |
| GSM2236076 | 2.876712329 | 0 | 1.374222588 | high |
| GSM2236078 | 11.67123288 | 0 | 0.273321055 | low |
| GSM2236079 | 11.5890411 | 0 | 0.615853561 | low |
| GSM2236084 | 10.52054795 | 0 | 0.123305313 | low |
| GSM2236085 | 12.49315068 | 0 | 0.5716662 | low |
| GSM2236087 | 12.73972603 | 0 | 0.766896423 | low |
| GSM2236088 | 2.794520548 | 1 | 0.376873499 | low |
| GSM2236090 | 2.547945205 | 1 | 0.619515424 | low |
| GSM2236094 | 3.04109589 | 1 | 0.113467342 | low |
